# Genomic and transcriptomic evidence for scavenging of diverse organic compounds by widespread deep-sea archaea

**DOI:** 10.1038/ncomms9933

**Published:** 2015-11-17

**Authors:** Meng Li, Brett J. Baker, Karthik Anantharaman, Sunit Jain, John A. Breier, Gregory J. Dick

**Affiliations:** 1 Department of Earth and Environmental Sciences, University of Michigan, Ann Arbor, Michigan 48109, USA; 2 Institute for Advanced Study, Shenzhen University, Shenzhen 518060, China; 3 Department of Marine Science, University of Texas Austin, Marine Science Institute, 750 Channel View Drive, Port Aransas, Texas 78373, USA; 4 Woods Hole Oceanographic Institution, Woods Hole, Massachusetts 02543, USA; 5 University of Texas Rio Grande Valley, Brownsville, Texas 78520, USA; 6 Department of Ecology and Evolutionary Biology, University of Michigan, Ann Arbor, Michigan 48109, USA; 7 Center of Computational Medicine and Bioinformatics, University of Michigan, Ann Arbor, Michigan 48109, USA

## Abstract

Microbial activity is one of the most important processes to mediate the flux of organic carbon from the ocean surface to the seafloor. However, little is known about the microorganisms that underpin this key step of the global carbon cycle in the deep oceans. Here we present genomic and transcriptomic evidence that five ubiquitous archaeal groups actively use proteins, carbohydrates, fatty acids and lipids as sources of carbon and energy at depths ranging from 800 to 4,950 m in hydrothermal vent plumes and pelagic background seawater across three different ocean basins. Genome-enabled metabolic reconstructions and gene expression patterns show that these marine archaea are motile heterotrophs with extensive mechanisms for scavenging organic matter. Our results shed light on the ecological and physiological properties of ubiquitous marine archaea and highlight their versatile metabolic strategies in deep oceans that might play a critical role in global carbon cycling.

## Abstract (web summary)

The contribution of marine archaea to the ocean's carbon cycle is unclear. Here, Li *et al*. analyse the genomes and transcriptomes from five deep-sea archaeal groups to reveal their metabolic characteristics, suggesting a crucial role in modulating the carbon cycle in deep oceans.

## Graphical abstract

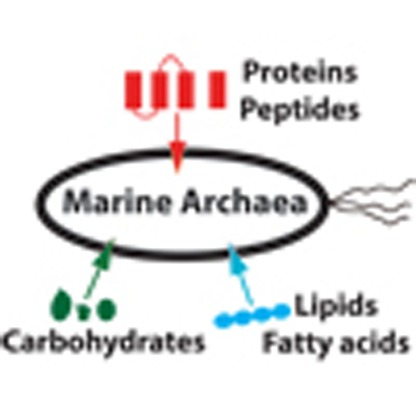

Archaea are ubiquitous members of marine microbial communities^1^,^2^,^3^. Four major groups of planktonic archaea have been reported in the global ocean, including Marine Group I Thaumarchaeota (MG-I)^2^,^3^, Marine Group II Euryarchaeota (MG-II)^4^, Marine Group III Euryarchaeota (MG-III)^4^ and Marine Group IV Euryarchaeota (MG-IV)^5^. While MG-III and MG-IV are predominately found in the deep oceans at relatively low abundance^4^,^5^, qualitative and quantitative studies suggest that MG-II are abundant in surface waters^4^,^6^,^7^, whereas MG-I dominates at greater depths, sometimes constituting up to nearly 40% of marine microbial plankton^8^.

Out of the four major groups of planktonic archaea, only representatives of MG-I have been cultured, which led to the discovery that they oxidize ammonia^9^,^10^. The MG-I are now generally recognized as the major drivers of nitrification in marine environments^11^,^12^,^13^. Up till now, all MG-I cultures oxidize ammonia and fix carbon, but there is also evidence for heterotrophy or mixotrophy by this group^10^,^14^,^15^,^16^. In contrast to the relatively well-studied MG-I, the physiology and energy metabolism of MG-II, MG-III and MG-IV remains poorly understood. Recent evidence indicates that MG-II can use organic carbon in the surface oceans^14^,^17^, suggesting that archaea may play an important role in the marine carbon cycle. However, little is known about the heterotrophic metabolism of archaea in the mesopelagic and bathypelagic realms of the ocean^18^, which comprise about 70% of ocean volume, account for the majority of marine microbial biomass and productivity^19^, and contain huge numbers of archaea^8^.

In this study, we reconstructed 59 partial to near-completed genomes and transcriptomes of several ubiquitous uncultured archaea groups from deep-sea hydrothermal plumes and surrounding background seawater at three distinct locations. Hydrothermal vent plumes are hotspots of biogeochemical activity in the deep oceans^20^ yet they are composed largely of background deep-sea microorganisms, including archaea^21^,^22^,^23^. Thus, plumes represent a valuable environment for studying deep-sea microorganisms. Our results reveal metabolic characteristics of these ubiquitous marine archaea and suggest that they play critical roles in modulating carbon cycle in deep oceans.

## Results

### Genomes and transcriptomes of deep-sea archaea

We conducted shotgun metagenomic and metatranscriptomic sequencing on samples from deep-sea hydrothermal vent plumes and surrounding background seawaters at Mid-Cayman Rise in the Caribbean Sea, Guaymas Basin in the Gulf of California and Eastern Lau Spreading Center in the Western Pacific Ocean (Supplementary Table 1). *De novo* assembly of metagenomic reads (Supplementary Table 2) and binning by tetranucleotide signatures revealed 32 archaeal genomic ‘bins' containing an estimated total of 59 archaeal genomes (Supplementary Fig. 1 and Supplementary Table 3)^24^,^25^. Estimates of genome completeness using an inventory of single-copy conserved genes^26^ indicate that 26 are more than 70% complete and 18 are 50–70% complete (Supplementary Tables 3 and 4). Phylogenetic analysis revealed the presence of five distinct groups, including 18 genomes from MG-I, 31 from MG-II, 5 from MG-III, 3 from Parvarchaeota, and 2 from putative Deep-sea Hydrothermal Vent Euryarchaeaotic Group-6 (DHVEG-6) (Table 1 and Supplementary Figs 2–4).

Comparative genomics showed that the five MG-I genomic bins had 53 to 59% average amino acid identity to the cultured *Nitrosopumilus maritimus* SCM1 (refs 27, 28). One genomic bin (Guaymas69) was the same MG-I population as previously reported^29^, while other four bins (Lau19, Guaymas96, Cayman117 and Cayman91) formed a separate deep-sea clade of MG-I (Supplementary Fig. 5). MG-II genomic bins clustered into three clades, congruent with the phylogeny of 16S rRNA genes (Supplementary Figs 2–5). Consistent with previous results^30^,^31^, the deep-sea MG-II did not contain genes for proteorhodopsin, a light-driven proton pump present in MG-II from the photic zone, indicating that the deep MG-II are not capable of photoheterotrophic metabolism proposed for surface MG-II (refs 17, 31, 32, 33). Three MG-III genomic bins (Guaymas31/92/93) formed a unique lineage with lower similarity to the fourth bin (Guaymas32, 48%) and two partial single cell genomes (Supplementary Fig. 5). Genomic bins related to Parvarchaeota were distantly related to *Parvarchaeota acidiphitum* ARMAN-4 from acid mine drainage^34^ and *Nanoarchaeota* DUSEL1 SCGC-AAA011D5 from the Homestake Mine in South Dakota^26^. We also obtained a genomic bin belonging DHVEG-6, for which there are no publicly available genomes (Supplementary Table 3).

### Extracellular peptidases in deep-sea archaea

**Figure 1:**
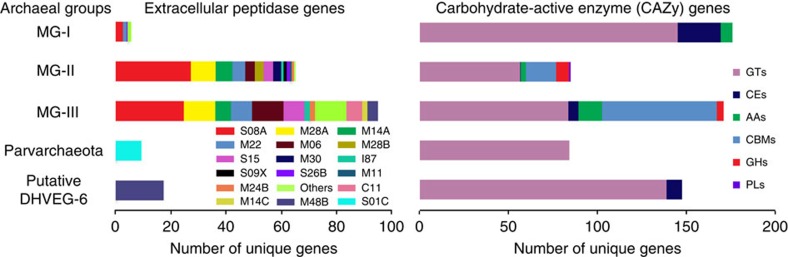
Genes for extracellular peptidases and carbohydrate-active enzymes. Carbohydrate-active enzymes (CAZYs) include glycoside hydrolases (GHs), glycosyl transferases (GTs), polysaccharide lyases (PLs), carbohydrate esterases (CEs), auxiliary activities (AAs) and carbohydrate-binding modules (CBMs). The two bar graphs indicate the unique genes (60% amino acids identity) for extracellular peptidases and CAZYs identified in five archaeal groups, and the general function description for extracellular peptidases and carbohydrate-active enzymes is listed in Supplementary Tables 5–7. The gene number has been normalized to the total number of genes recovered from each archaeal group.

Our data indicate that these deep-sea archaea are metabolically active as aerobic heterotrophs or, in the case of MG-I, mixotrophs. Genes encoding 28 different families of extracellular peptidases for protein degradation were identified. Four of these families were dominant (S08A, M28A, M14A and M22), comprising 70.5%, 72% and 52% of total extracellular peptidase genes in deep-sea MG-I, -II and -III, respectively (Fig. 1, Supplementary Fig. 6a and Supplementary Table 5). Transcripts of these extracellular peptidase genes were among the most abundant in the transcriptomes of MG-II and MG-III in both Guaymas and Cayman plumes (Supplementary Figs 6a and 7). For MG-I, in addition to the S08A and M22 peptidase families, transcripts for M01, S26A and S26B were also detected (Supplementary Table 5), though the most abundant transcripts in the MG-I transcriptome were related to the transport and oxidation of ammonia (Supplementary Fig. 8). Interestingly, the suite of specific extracellular peptidases for which transcripts were detected varied between archaeal groups (Supplementary Fig. 6a), suggesting diverse protein utilization capabilities by MG-I, -II and -III in the deep oceans^35^.

**Figure 2:**
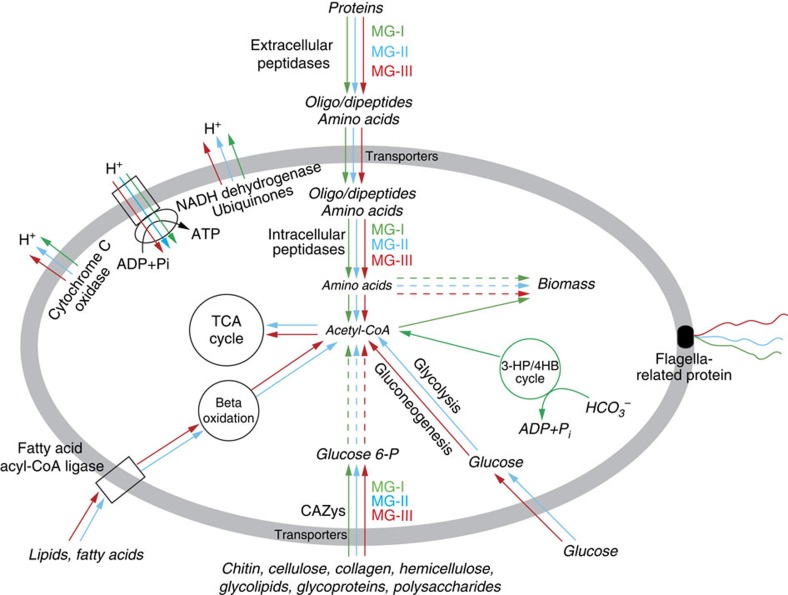
Proposed transcriptionally active heterotrophic metabolic pathways. Green, blue and red indicate heterotrophic pathways found in MG-I, MG-II and MG-III, respectively.

In contrast to MG-I, -II and -III, the genomic bins of Parvarchaeota and DHVEG-6 contained only one gene related to the S01C family (DegP peptidase) and two genes affiliated with M48B family (HtpX peptidase), and no transcripts of these genes were detected (Supplementary Table 5). These results indicate that (1) MG-I, -II, -III, Parvarchaeota and DHVEG-6 are all capable of degrading and utilizing extracellular proteins; (2) protein degradation is a major metabolic pathway for MG-II and -III while a minor metabolic route for MG-I; and (3) the different archaeal groups each have a different suite of protein degradation genes, suggesting niche differentiation on the basis of substrate (Table 1 and Fig. 2).

Although many of the peptidases identified here are divergent from experimentally studied proteins, family-level classifications offer clues to their broad functions. S08A peptidases, one of the largest groups of serine endopeptidases^36^, clustered into four major phylogenetic groups: alkaline proteases, oxidant-stable proteases, Bacillopeptidases F-like proteinases and unclassified archaea subtilisin (Supplementary Fig. 9). S08A are N-terminus peptidases containing a catalytic triad sequence of Asp, His and Ser. M28A peptidases are zinc-dependent exopeptidases that selectively cleave N-terminal amino acids from polypeptides^37^,^38^. M28A-related genes in MG-II and -III fell into six clusters (Supplementary Fig. 10). Clusters 1 and 6 had abundant transcripts but their specific functions are unknown. M14A peptidase is a group of zinc-containing proteolytic enzymes (including carboxypeptidase A), which catalyses the hydrolysis of C-terminal peptide bonds in protein and peptide substrates^39^. Genes encoding the M14A family in MG-II and -III were divided into three clusters that are each significantly different from known carboxypeptidase A (Supplementary Fig. 11). This extensive suite of extracellular enzymes that cleave peptides at different sites suggests comprehensive protein degradation by deep-sea MG-II/III and potential use of protein as a growth substrate.

### Carbohydrate-active enzymes (CAZYs) in deep-sea archaea

The archaeal genomes also contained diverse and transcriptionally active carbohydrate-active enzymes (Fig. 1, Supplementary Fig. 6b and Supplementary Table 6). In MG-I, Parvarchaeota and DHVEG-6 we identified glycosyl transferases, carbohydrate esterases and other auxiliary activities that may be involved in the modification or creation of glycosidic bonds in various form of organic matter within cells^40^. However, genes related to glycoside hydrolases and carbohydrate-binding modules were more diverse and common in MG-II and -III (Supplementary Table 7), suggesting they are also involved in the hydrolysis of glycosidic bonds in complex sugars^40^,^41^. Similar to our observations of differential expression of peptidases in MG –II and –III, we observed that α-mannosidase (GH38), amylopullulanase (GH57), 4-α-glucanotransferase (GH77) and chitinases (GH18/CBM5 and GH20/CBM5) were abundant in the MG-II transcriptome, while only chitinases (GH18/CBM5 and GH20/CBM5) were transcriptionally active in MG-III, suggesting differentiation of carbohydrate use between archaeal groups (Fig. 2 and Supplementary Table 7).

### Lipid metabolism in deep-sea archaea

Archaeal genes involved in beta-oxidation of fatty acids were present and abundantly expressed in deep-sea MG-II and -III, suggesting that straight chain lipids may also be organic substrates for these groups (Supplementary Table 8 and Fig. 2). A recent study concluded that MG-II can synthesize crenarchaeol^42^, a tetraether lipid widely used in paleoceanography and previously considered to be a unique biomarker for MG-I (refs 43, 44). Here, we found the key gene for tetraether lipid biosynthesis, geranylgeranylglyceryl phosphate (GGGP) synthase, in MG-I, -II and -III genomes (Supplementary Table 9). Phylogenetic analysis indicated that GGGP synthase genes from MG-II and MG-III were different from that of MG-I, forming two separate clusters (Supplementary Fig. 12). The genetic basis for tetraether lipid synthesis requires further study in order to better understand the lipid biosynthetic potential of these different groups, and how the pathways are regulated as a function of environmental conditions. In addition, we also identified MG-II genes involved in synthesis of archaeal ether-like lipids, polyunsaturated fatty acids and glycerophospholipids (Supplementary Table 10).

### Cell mobility and central metabolism in deep-sea archaea

Genes involved in cell mobility were identified in deep-sea MG-I, –II and –III genomes (Fig. 2 and Supplementary Table 11). Particularly in MG-II and -III, several flagellar genes with different transcriptional patterns were identified as operons in the same contig (Supplementary Table 11 and Supplementary Fig. 13), suggesting that these ubiquitous deep-sea archaea have active flagella, which might facilitate acquisition of organic substrates via chemotaxis^45^ or attachment to particles^46^.

Finally, genes related to central carbon metabolism were also identified in these genomes and transcriptomes, including the tricarboxylic acid cycle and glycolysis/gluconeogenesis pathway in MG-II and MG-III, and the modified 3-hydroxypropionate/4-hydroxybutyrate (3-HP/4-HB) cycle in deep-sea MG-I (Table 1, Fig. 2 and Supplementary Table 12). These results further highlight that these ubiquitous deep-sea archaea have versatile metabolic pathways that are important for carbon utilization in the deep oceans.

## Discussion

Overall, the genomic and transcriptomic results presented here indicate that widespread deep-sea archaea utilize diverse proteins, carbohydrates and straight chain lipids as substrates for heterotrophic growth (Table 1 and Fig. 2). Although our previous work on hydrothermal plumes identified the major groups responsible for production of organic matter through chemosynthesis^21^,^25^,^29^,^47^, the results presented here indicate that archaea play a role in consuming that fresh organic matter, which can be regionally significant^20^. Further, because these archaea are widespread throughout the deep ocean, they likely contribute to the broader marine carbon cycle.

Microbial degradation of organic matter in the pelagic ocean-water column strongly influences the export of carbon from the surface oceans, and converts labile carbon into refractory organic matter that can be preserved in deep-sea sediments or as dissolved organic carbon (DOC)^48^,^49^,^50^,^51^,^52^. The deep-sea DOC reservoir is massive, holding a quantity of carbon that is roughly equivalent to that in the atmosphere. Recent evidence suggests that 30% of the deep DOC reservoir is modern and actively cycled on short time scales^53^. Although the contributions of deep-sea archaea to such processes remain to be quantified, our findings demonstrate an active archaeal response to pulses of organic matter in the deep sea that may shunt carbon from proteins and carbohydrates to CO_2_ and lipids. Because archaea are ubiquitous across varying geochemical regimes and depths of the world's deep oceans^30^,^54^, these results implicate archaea as key players in heterotrophy and provide new mechanistic perspectives on carbon cycling in the deep oceans.

## Methods

### Sample collection

Samples from Mid-Cayman Rise in the Caribbean Sea were collected on the cruises abroad *R/V Atlantis* in January 2010. Samples at different depths of the rising plumes from Von Damm (∼2200, m) and Beebe (∼5000, m) hydrothermal vent fields as well as their background were collected using a Suspended Particle Rosette Samples (SUPR) mounted on remotely operated vehicle ROV Jason II, which can be used for sampling rising plumes^55^,^56^. In brief, water samples collected with SUPR (10–60 l) from different seawater depths were filtered on to 0.2-μm pore size 142-mm polycarbonate SUPOR membranes and preserved in RNAlater *in situ* (Supplementary Table 1). Samples from Guaymas Basin and Eastern Lau Spreading Center were collected aboard several cruises using Niskin bottles (Guaymas) and *in situ* filtration with the SUPR sampler (Lau)^25^,^56^,^57^,^58^. The details of samples and the sampling locations are provided in Supplementary Table 1.

### Extraction of nucleic acids and sequencing

DNA and RNA were extracted from ¼ filters following procedures^21^,^57^. For DNA extraction, the ¼ filters was cut into pieces and added to a 1.5 ml tube containing 200 mg each of 0.1, 0.5 and 2 mm zirconium beads. Then, 580 ml of lysis solution (300 mM EDTA, 300 mM NaCl, 300 mM Tris buffer, pH 7.5), 70 ml of 15% SDS and 35 ml of 1 M DTT in 0.01 M Na acetate were added. Tubes were vortexed, incubated at 70 °C for 30 min and cooled to <40 °C. 14 ml of 5% lysozyme (w/v in water) was added and the tubes were incubated at 37 °C for 20 min then beat on a FastPrep bead beater machine for 45 s at setting 6.5. After that, 150 ml of 1 M KCl was added, and the tubes were placed on ice for 2–5 min after vortex. The mixture was then centrifuged for 5 min at 14,000 *g*, clear supernatant was transferred to a Montage PCR purification filter unit and centrifuged at 1,000 *g* until all of the liquid passed through (∼15 min). The flow-through was discarded, and 400 ml of TE buffer was added to the filter unit and it was centrifuged for 15 min at 1,000 *g*. The DNA was then eluted in 100 ml of TE and quantified using RiboGreen (Invitrogen). RNA was extracted using the mirVana mRNA Isolation kit (Ambion), treated with DNase I to remove DNA and concentrated and repurified using the RNeasy MinElute Kit (Qiagen). Total RNA was quantified using RiboGreen (Invitrogen). RNA amplification by random priming and complementary DNA synthesis was performed using the MessageAmp II-Bacteria Kit (Ambion). Shotgun sequencing of DNA and complementary DNA were performed with Illumina HiSeq2000 at the University of Michigan DNA Sequencing Core, and results are shown in Supplementary Table 1.

### *De novo* metagenomic assembly and gene annotation

The raw shotgun sequencing metagenomic reads were deprelicated (100% identity over 100% lengths) and trimmed using Sickle (https://github.com/najoshi/sickle). Samples from the eight vent sites (Guaymas, Von Damm (Cayman Shallow), Beebe (Cayman Deep), Kilo Moana, Abe, Mariner, Tahi Moana, Tui Malila) were each assembled *de novo* to obtain eight separate assemblies (Supplementary Table 2). Whole genome *de novo* assemblies were performed using IDBA-UD^59^ with the following parameters: –mink 50, –maxk 92, –steps 4 or 6, –min_contig 500. Gene calling and annotations were done using the DOE Joint Genome Institutes (JGI) Integrated Microbial Genomes pipeline (http://img.jgi.doe.gov/cgi-bin/w/main.cgi).

### Metagenome binning and metatranscriptomic data analysis

Binning of assembled metagenomic sequences was performed using a combination of tetranucleotide frequencies, contig coverage and %GC content in emergent self-organizing maps^24^. A total of 32 genomic bins from eight assemblies were identified as archaeal bins (Supplementary Fig. 1). Based the inventory of archaeal 162 single-copy conserved genes, 32 archaeal genomic bins were estimated to contain 59 partial to complete genomes (Supplementary Tables 3 and 4). The archaeal 16S and 23S rRNA genes and two ribosomal protein-coding genes from each archaeal genomic bin were further analysed for identifying their taxonomic positions (Supplementary Figs 2–4). Abundance of transcripts for each archaeal genomic bins was determined by mapping all non-rRNA transcripts to the assembled fragments using BWA with default settings^60^,^61^ and normalizing to their sequence length and total number of non-rRNA transcripts in each metatranscriptomic library.

### Comparative genomics

Comparative genomics of the deep-sea archaea identified in this study was performed against the representative archaeal genomes within each group (Supplementary Fig. 5). Genomic similarity with known archaea (*Nitrosopumilus maritimus* (CP000866)^27^ in MG-I; surface seawater uncultured marine group II euryarchaeote (CM001443)^17^ in MG-II; two single cell genomes of MG-III (SCGC-AAA007-O11 and SCGC-AAA288-E19) in MG-III, *Parvarchaea acidiphilium* ARMAN-4 (PRJNA38567)^34^ and SCGC-AAA011-D5 (ref. 26) in Parvarchaeota) was determined based on reciprocal best BLASTP hits between known genomes and archaeal genomic bins. The average identity (amino acid level) among each archaeal genomic bins and representative archaea genome were generated as Supplementary Fig. 5.

### Identification of key functional genes

For genes encoding peptidases and carbohydrate-active enzymes, all annotated genes in deep-sea archaea genomic bins were searched against public databases of peptidases (MEROPS)^62^ and CAZYs^63^ with *E*-value <10^−10^ by BLASTP. All hits were compared against the non-redundant NCBI protein database. Only those that had top hits to peptidases and carbohydrate active enzymes were considered. The extracellular peptidases were further confirmed based on the identification of extracellular transport signals using SignaIP^64^, POSRTb^65^ or PRED-SIGNAL^66^ (Supplementary Table 5). Genes related to carbohydrate active enzymes were further classified into different groups according the prediction of CAZYs (Supplementary Tables 6 and 7). Genes involved in lipid metabolic pathways (Supplementary Tables 8 and 9) and the central metabolism (Supplementary Table 10) in MG-I/II/III were identified using similar procedures based on the published gene databases^17^,^67^.

### Sequence alignment and phylogeny

Alignment of amino acid sequences for extracellular peptidase of S08A, M28 and M14A families was performed by MUSCLE using default parameters followed by manual refinement^68^. Phylogenetic analysis of representative peptidase of three families and GGGP synthases were inferred by Maximum Likelihood implemented in Mega 6.0^69^ using the Tamura-Nei and passion model after testing by ProTest^70^ and bootstrapped 1000 times (Supplementary Figs 9–12).

## Additional information

**Accession codes:** The nucleotide sequences have been deposited in the DOE JGI-IMG/MER database under the following Taxon Object IDs: 3300001680 (Kilo Moana), 3300001681 (Abe), 3300001678 (Mariner), 3300001679 (Tahi Moana), 3300001676 (Tui Malila), 3300001683 (Guaymas), 3300001781 (Cayman Deep) and 3300001835 (Cayman Shallow).

**How to cite this article:** Li, M. *et al*. Genomic and transcriptomic evidence for scavenging of diverse organic compounds by widespread deep-sea archaea. *Nat. Commun.* 6:8933 doi: 10.1038/ncomms9933 (2015).

## Supplementary Material

Supplementary Information Supplementary Figures 1-13 and Supplementary Tables 1-12

## Figures and Tables

**Table 1:** Overview of genomes from five archaeal groups recovered in this study and their ecophysiological characteristics.

**Archaea group**	**Number of genome bins**	**Number of genomes**	**Lithoautotrophic metabolisms**	**Heterotrophic metabolisms**
MG-I	6	18	3-Hydroxypropionate/4-hydrobutyrate cycle, ammonia oxidation	Protein degradation, carbohydrate metabolism
MG-II	19	31	Not detected	Protein degradation, carbohydrate metabolism, β-oxidation
MG-III	4	5	Not detected	Protein degradation, carbohydrate metabolism, β-oxidation
Parvarchaeota	2	3	Not detected	Protein degradation, carbohydrate metabolism
Putative DHVEG-6	1	2	Not detected	Protein degradation, carbohydrate metabolism

DHVEG-6, Deep-sea Hydrothermal Vent Euryarchaeaotic Group-6; MG, Marine Group.
