# Supplementary material for: Genomic and transcriptomic evidence for scavenging of diverse organic compounds by widespread deep-sea archaea
Source: Nat Commun. 2015 Nov 17;6:8933. doi: 10.1038/ncomms9933 (PMC4660358; doi:10.1038/ncomms9933)
Supplement: Supplementary Information — Supplementary Figures 1-13 and Supplementary Tables 1-12 [file ncomms9933-s1.pdf]

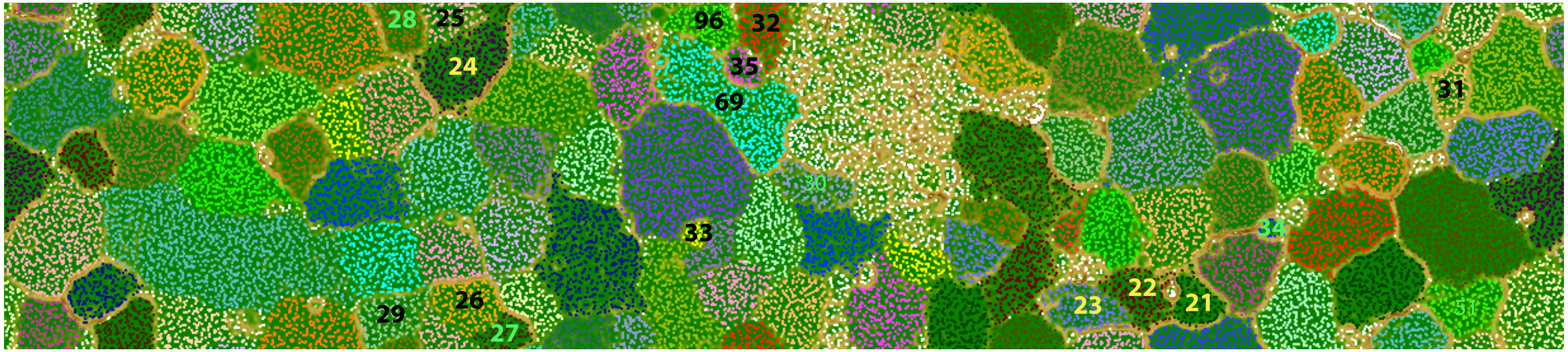

(a)

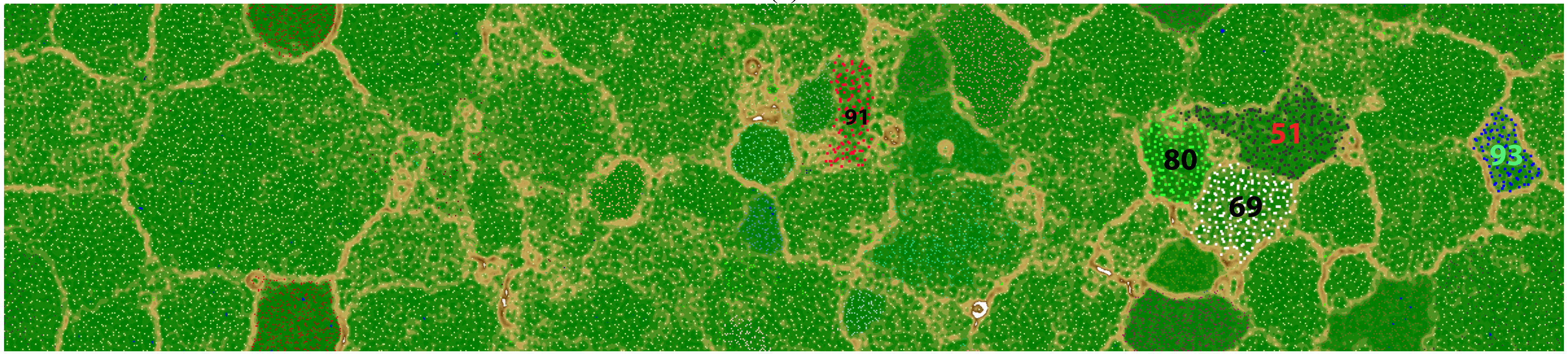

(b)

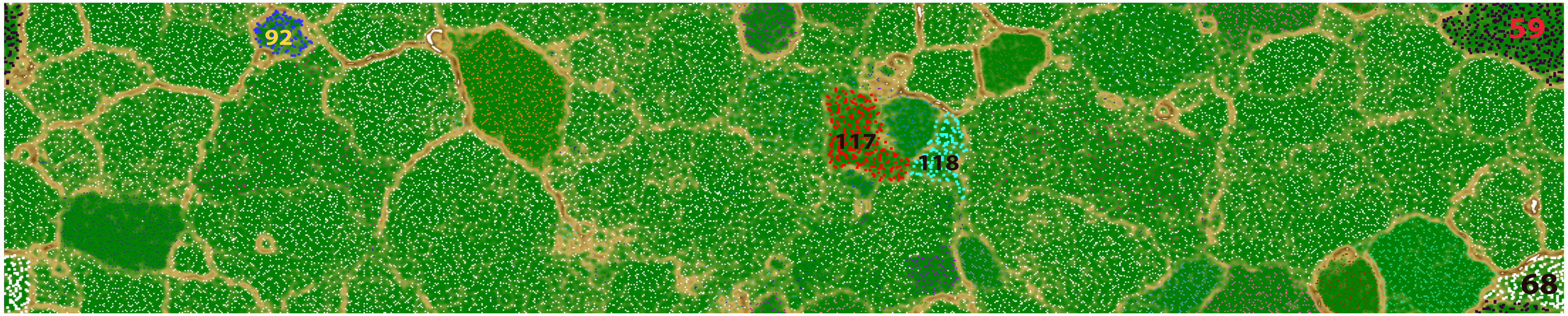

(c)

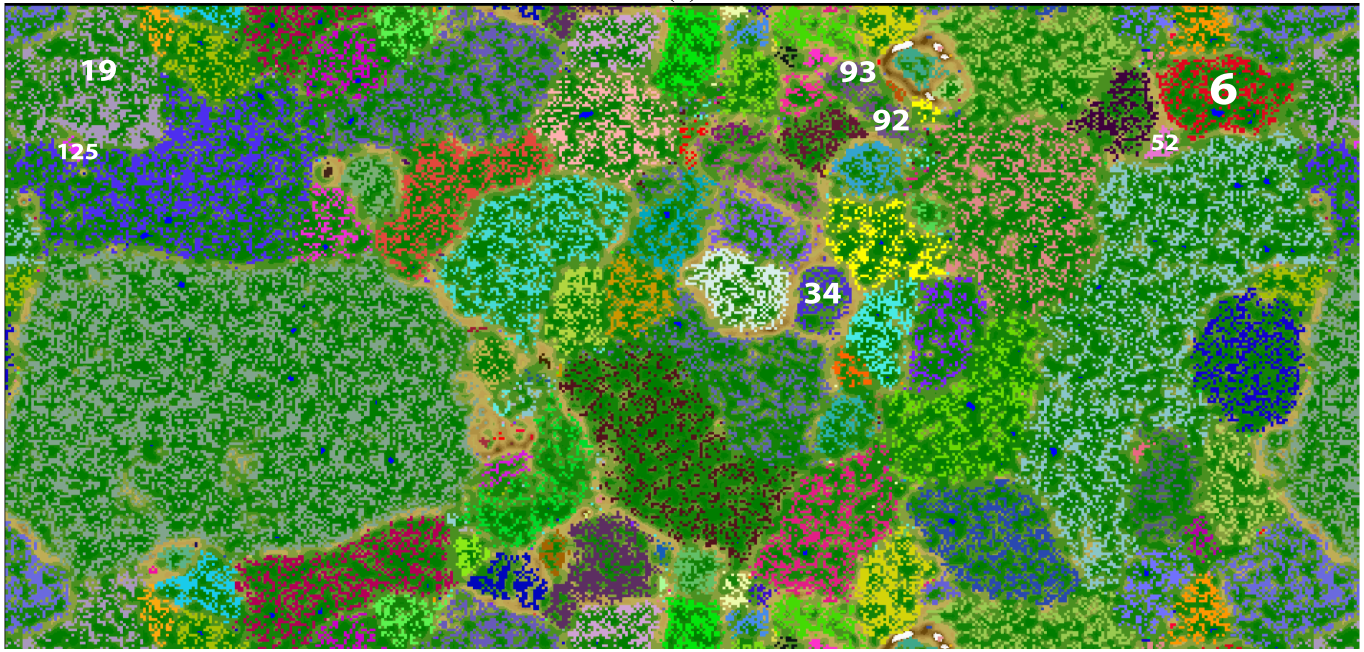

(d)

Supplementary Figure 1. ESOM maps of metagenomic assemblies for Guaymas (a), Cayman Deep (b), Cayman Shallow (c), and Lau (d). Each point on the map represents a contig (>4 kb) or contig fragment generated *in silico* (4-8 kb). All identified archaeal bins are uniquely color coded and marked with numbers as indicated in supplementary table 3.

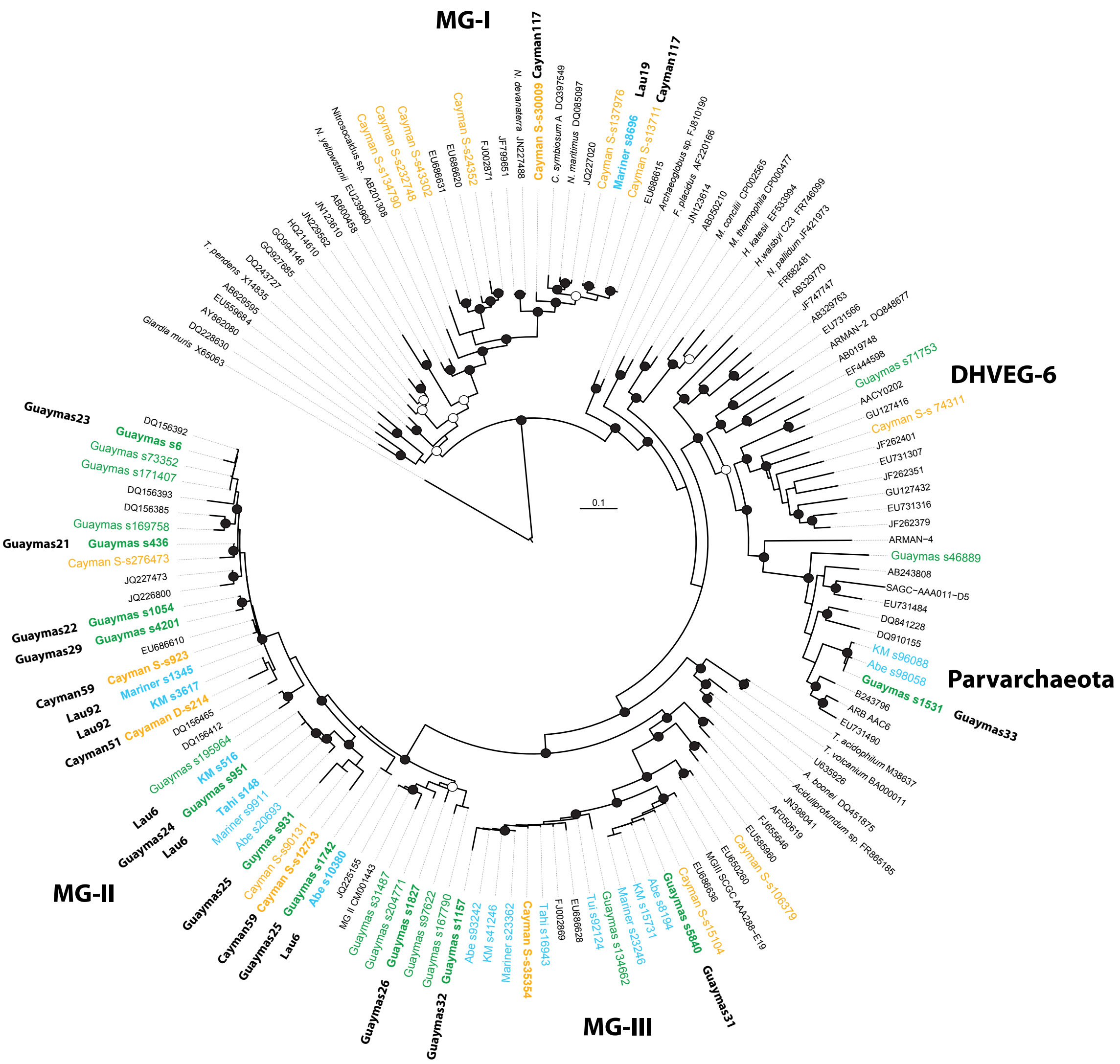

Supplementary Figure 2. The phylogeny of 16S rRNA gene assembled sequences from Cayman (yellow), Guaymas (green) and Lau Basin (blue). The bold sequences were identified from archaeal genomic bins as indicated. The dots on the tree indicate the bootstrap values that are more than 50%.

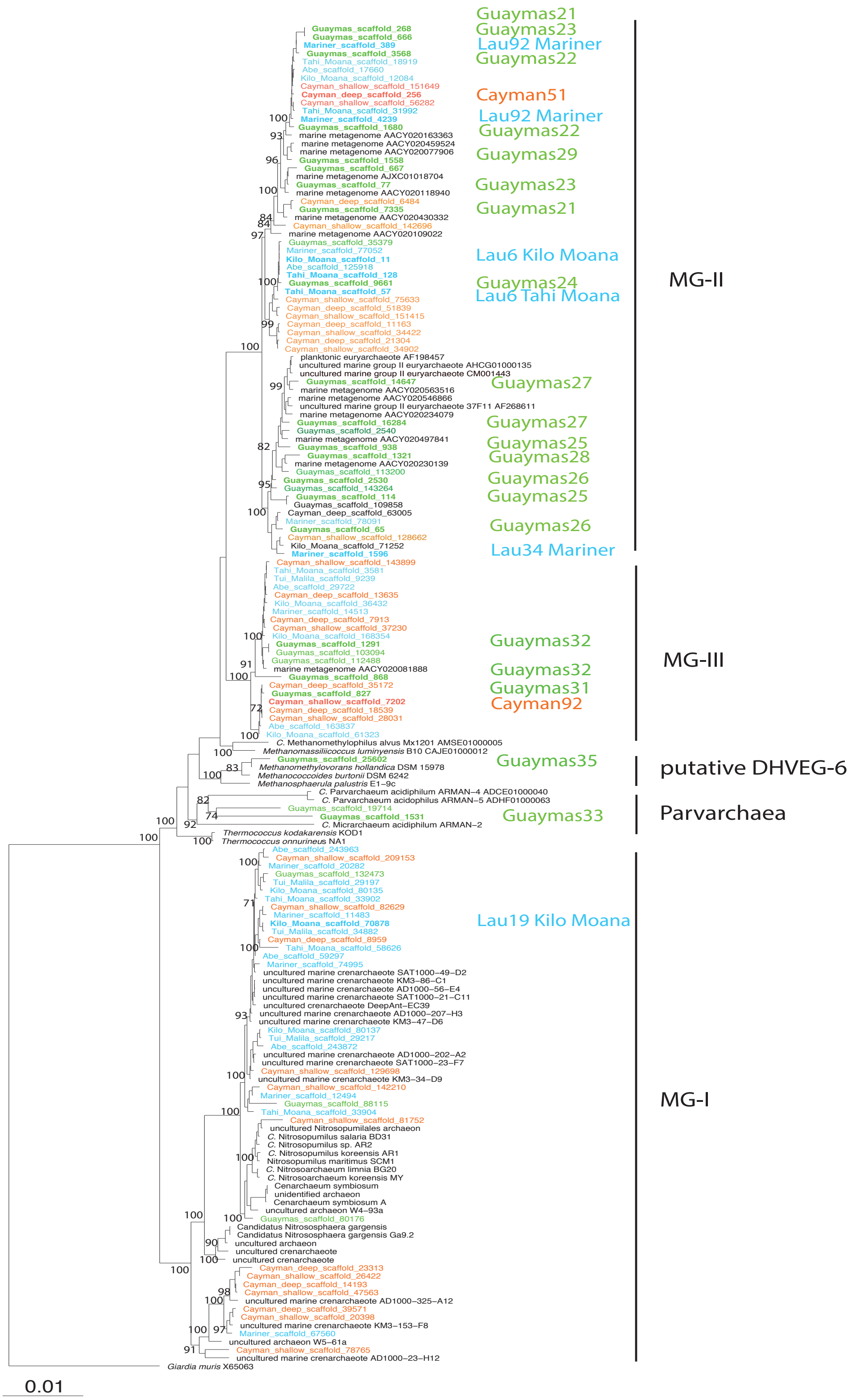

Supplementary Figure 3. The phylogeny of 23S rRNA gene assembled sequences from Cayman (yellow), Guaymas (green) and Lau Basin (blue). The bold sequences were identified from archaeal genomic bins as indicated.

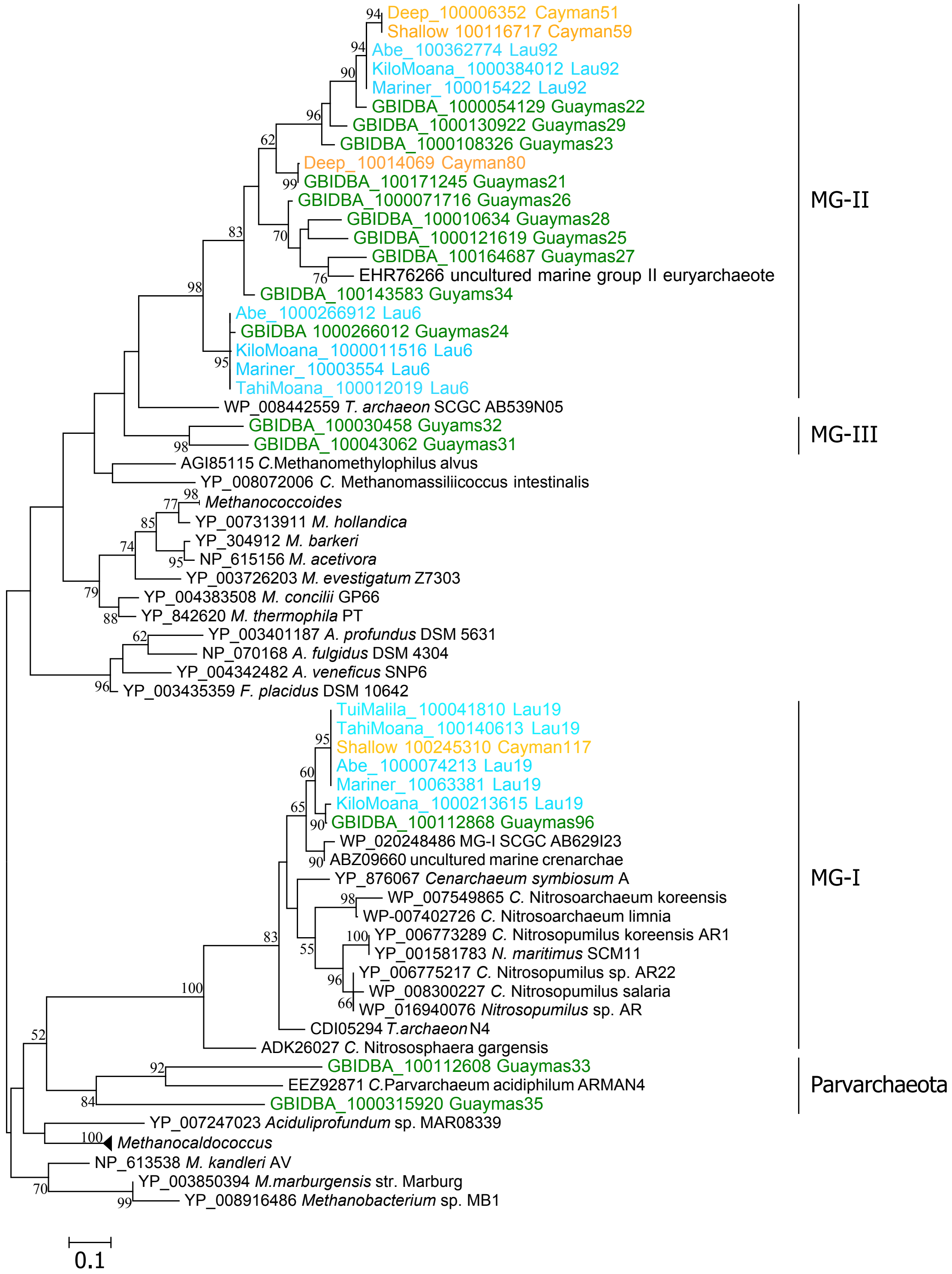

Supplementary Figure 4. Phylogenetic analysis of ribosomal protein (L10e/L16P) assembled sequences from Cayman (yellow), Guaymas (green) and Lau Basin (blue). The bold sequences were identified from archaeal genomic bins as indicated.

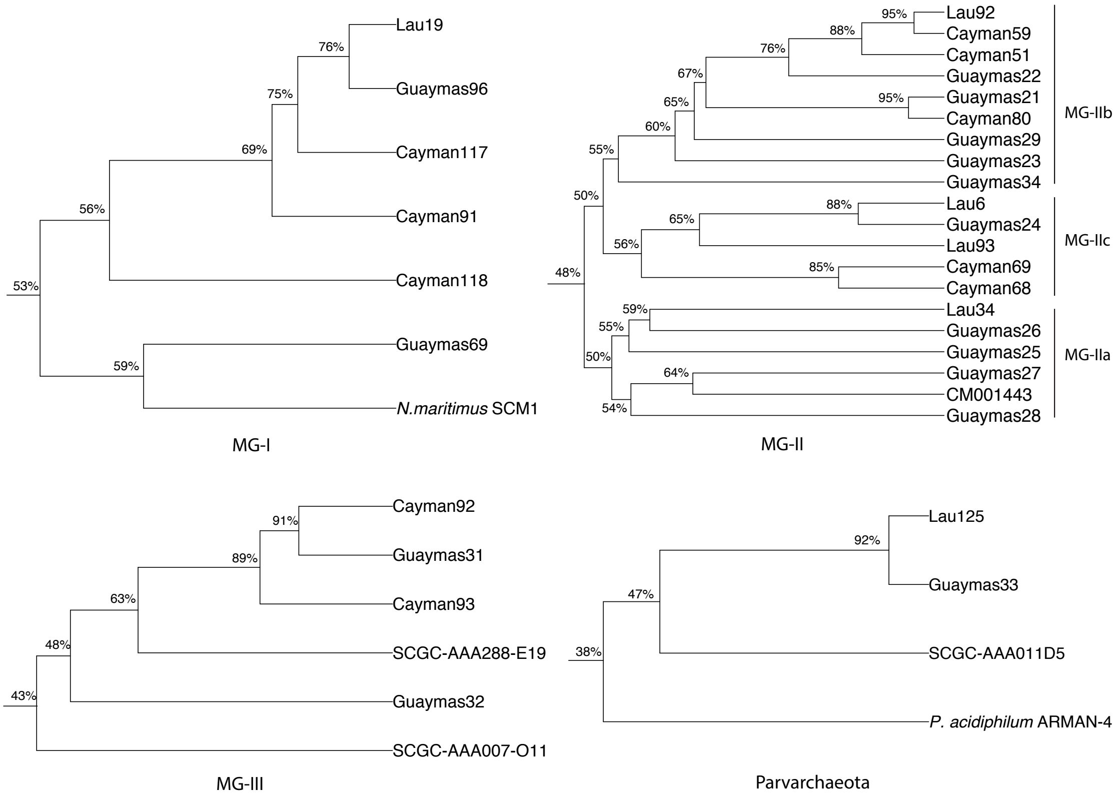

Supplementary Figure 5. Comparison between deep-sea archaea genomic bins and reference genomes within each group. The numbers on the tree branch indicate the average amino acids similarity of these archaea genomic bins within each archaea group through reciprocal blastP.

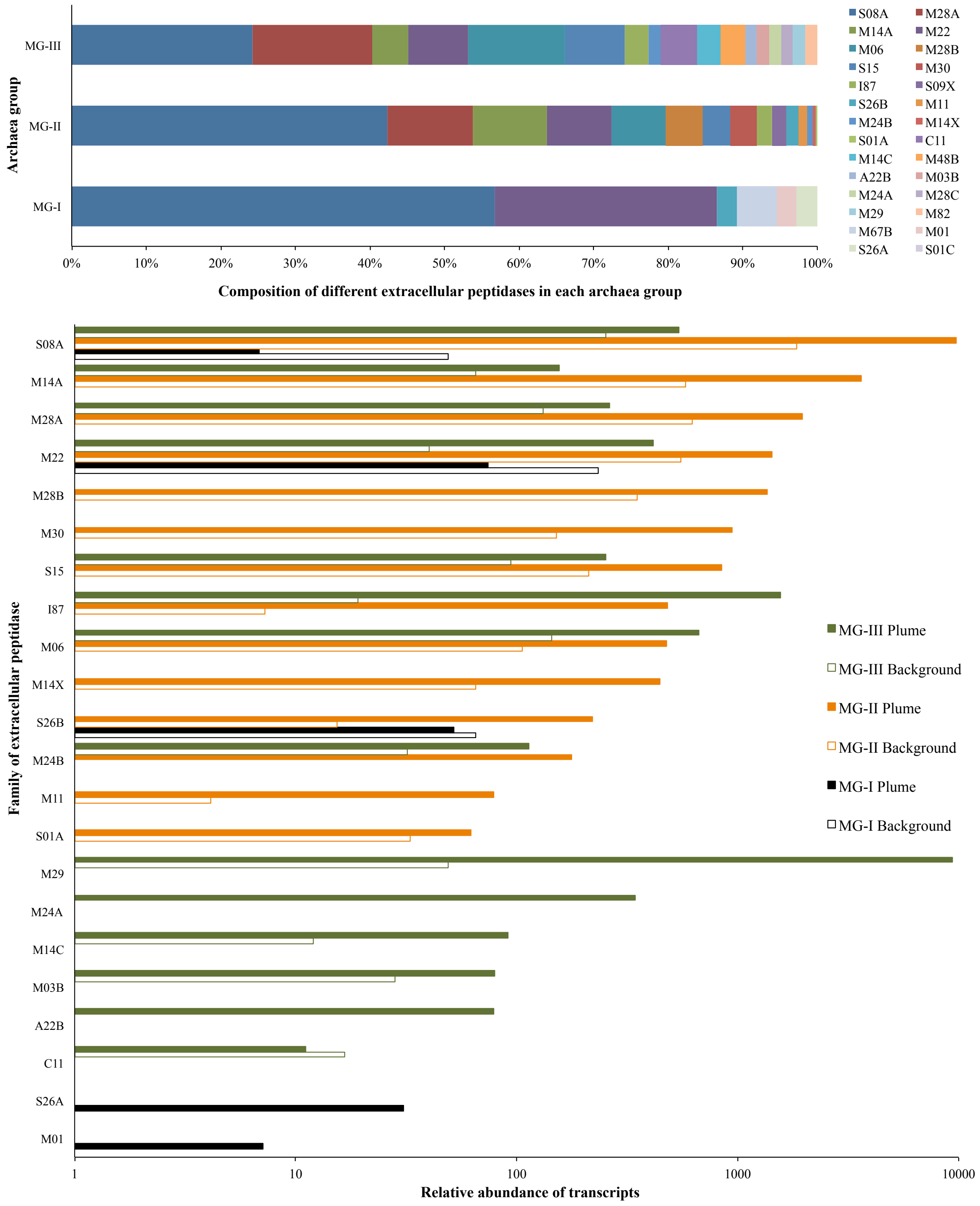
MG-III
MG-II
MG-I
Archaea group
0%
10%
20%
30%
40%
50%
60%
70%
80%
90%
100%
Composition of different extracellular peptidases in each archaea group
S08A
M28A
M14A
M22
M06
M28B
S15
M30
I87
S09X
S26B
M11
M24B
M14X
S01A
C11
M14C
M48B
A22B
M03B
M24A
M28C
M29
M82
M67B
M01
S26A
S01C
Family of extracellular peptidase
S08A
M14A
M28A
M22
M28B
M30
S15
I87
M06
M14X
S26B
M24B
M11
S01A
M29
M24A
M14C
M03B
A22B
C11
S26A
M01
MG-III Plume
MG-III Background
MG-II Plume
MG-II Background
MG-I Plume
MG-I Background
1
10
100
1000
10000
Relative abundance of transcripts

(a)

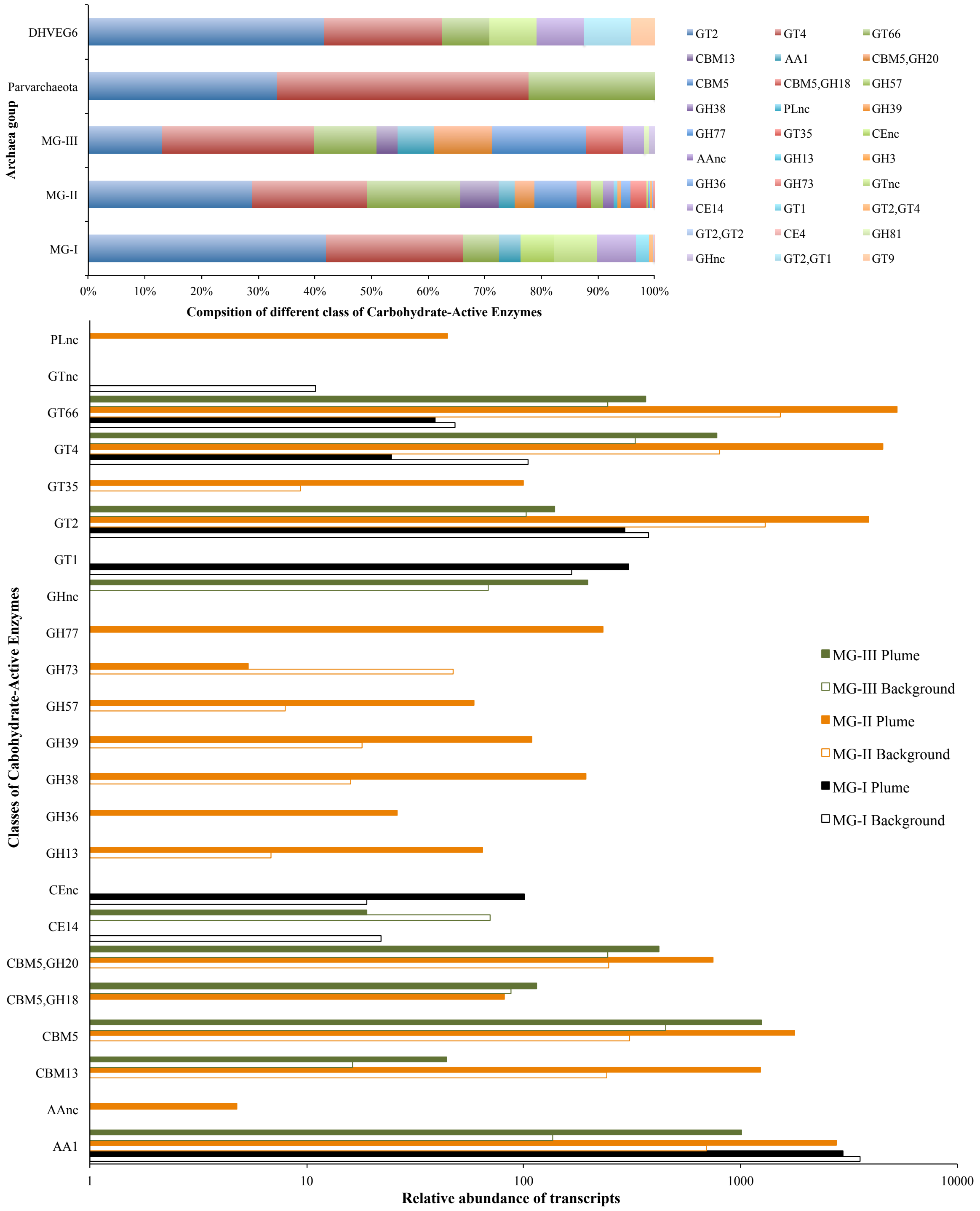

(b)

Supplementary Figure 6. Gene composition (the upper part) and transcriptional abundance (the lower part) of extracellular peptidase (a) and carbohydrate active enzymes (b) in deep-sea archaea.

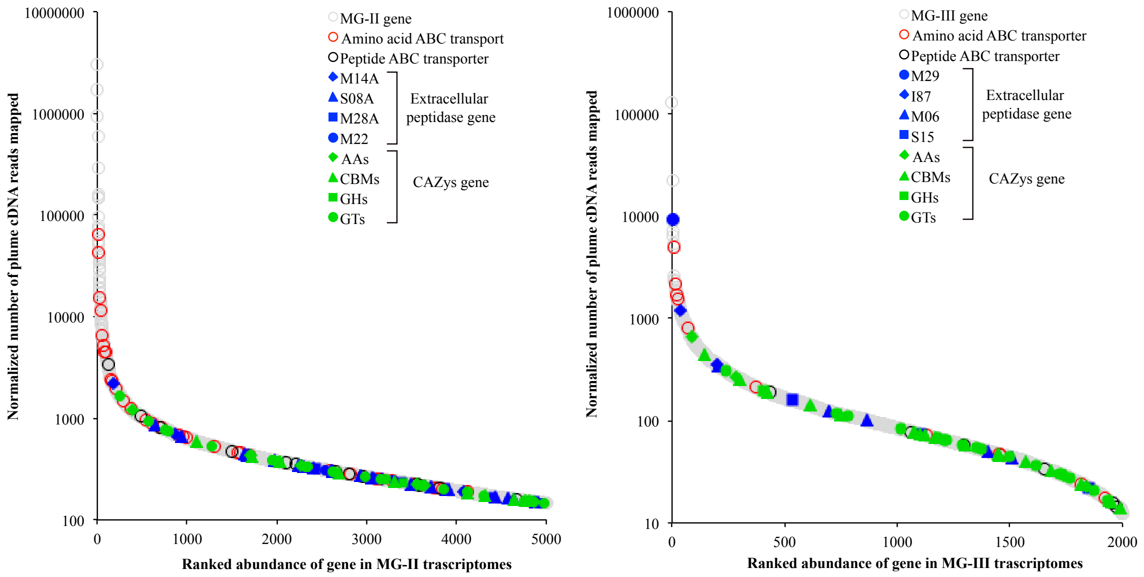

Supplementary Figure 7. Transcript abundance of extracellular peptidase and carbohydrate-active enzymes (CAZYs) genes in deep-sea MG-II (left) and MG-III (right). Genes for the top four families of extracellular peptidase (M14A, S08A, M28A and M22 for MG-II and M29, I87, M06 and S15 for MG-III) and CAZYs (Auxiliary Activities (AAs), Carbohydrate-Binding Modules (CBMs), Glycoside Hydrolases (GHs), and Glycosyl Transferases (GTs)) are highlighted in the figure.

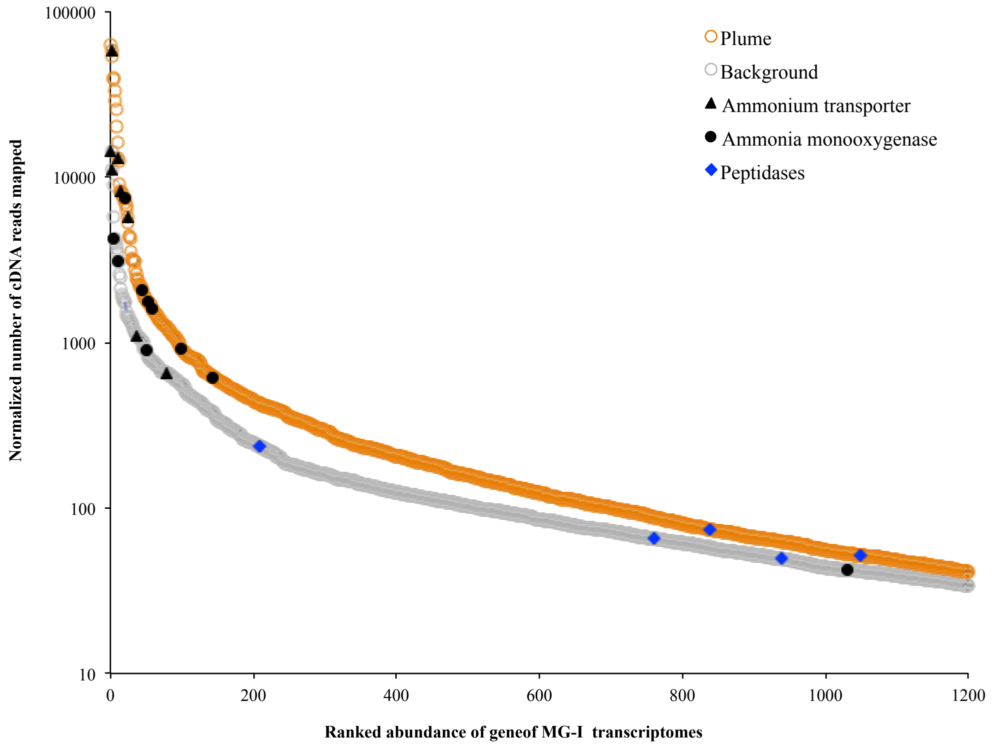

Supplementary Figure 8. Rank abundance of gene transcripts in deep-sea MG-I. Genes for ammonium transporters, ammonia monooxygenases and extracellular peptidases are highlighted in the figure.

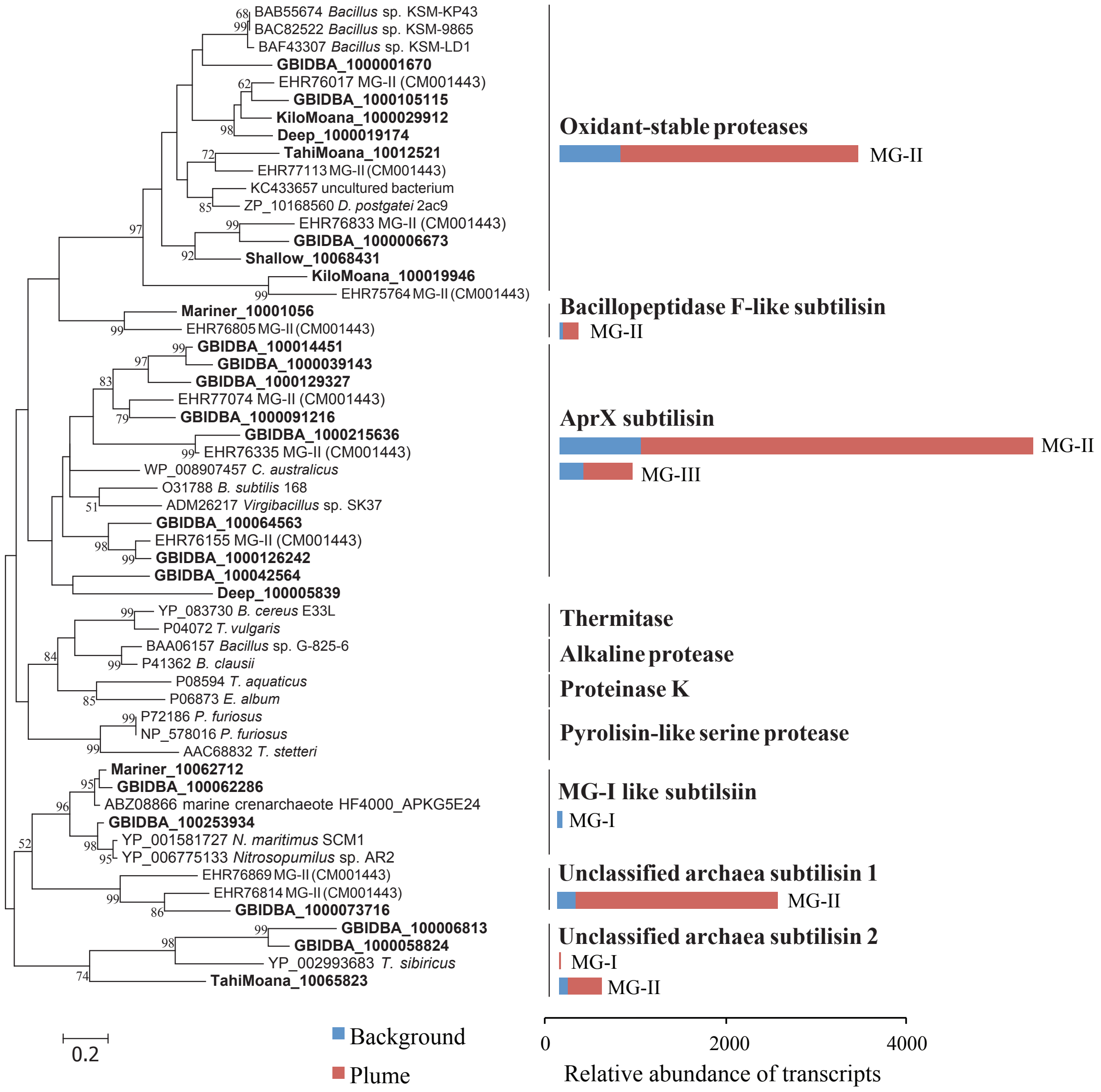

Supplementary Figure 9. Phylogenetic analysis of representative S08A family extracellular peptidase coding genes and their expression.

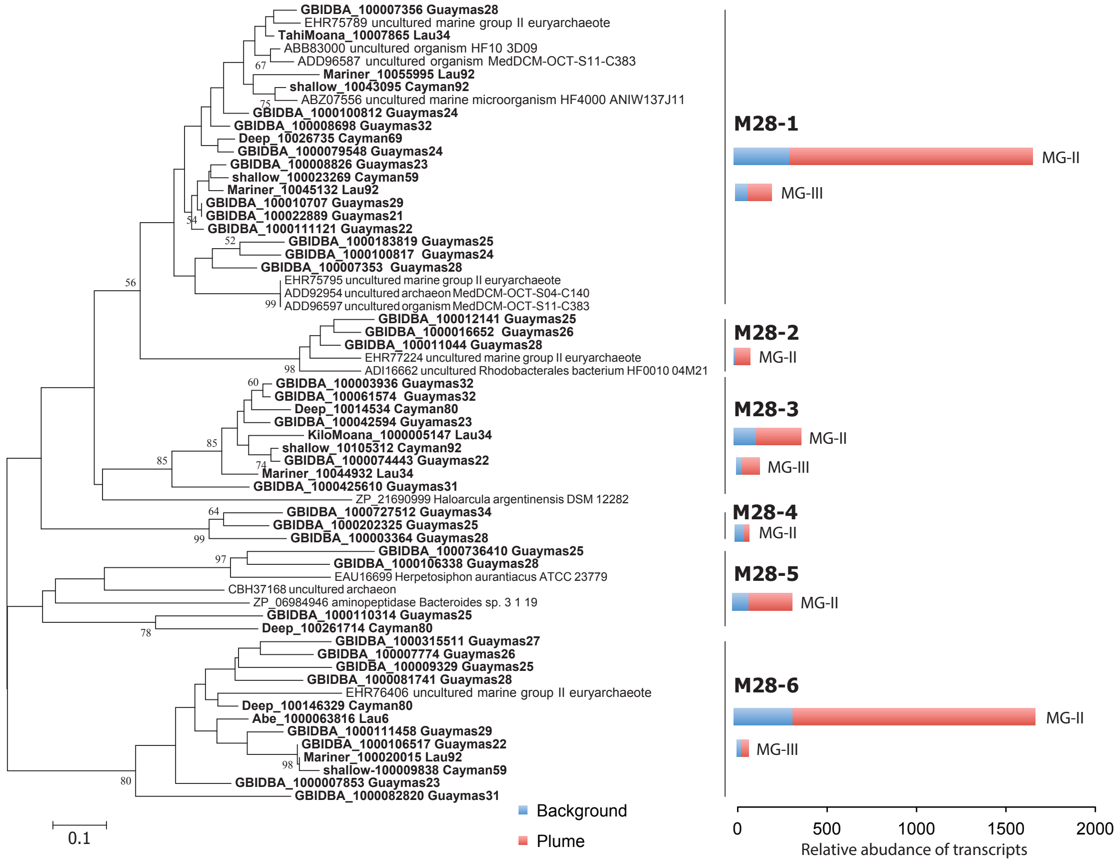

Supplementary Figure 10. Phylogenetic analysis of representative M28 family extracellular peptidase coding genes and their expression.

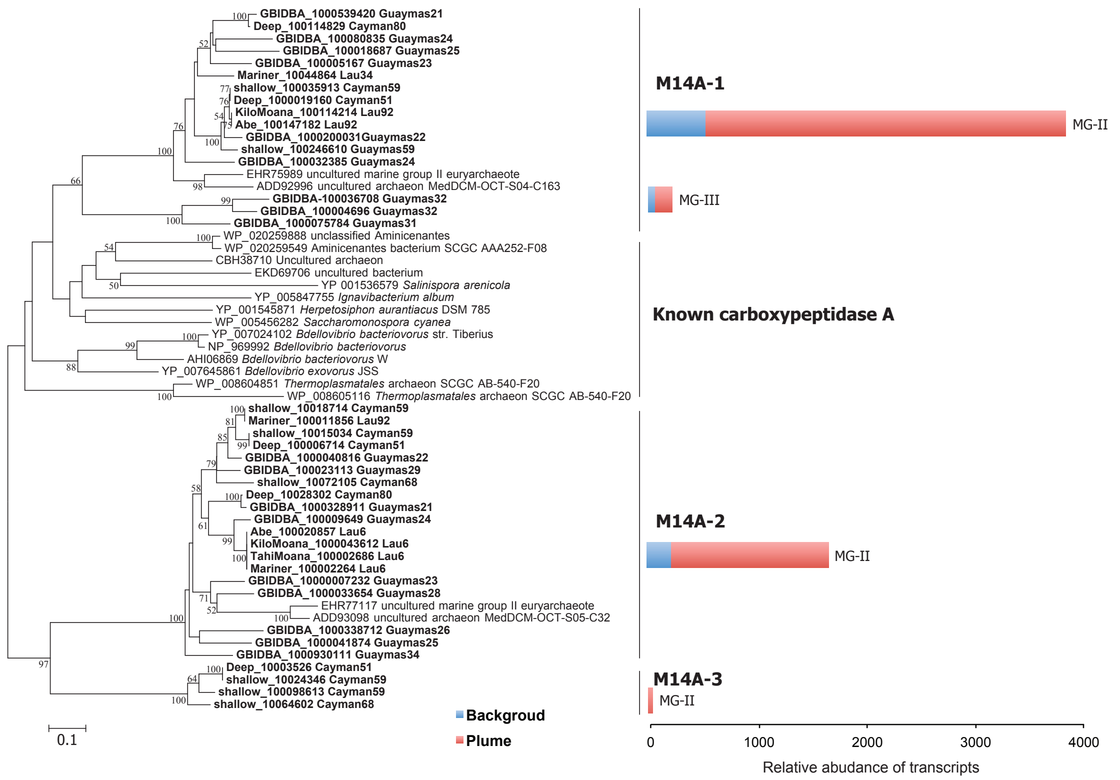

Supplementary Figure 11. Phylogenetic analysis of representative M14A family extracellular peptidase coding genes and their expression.

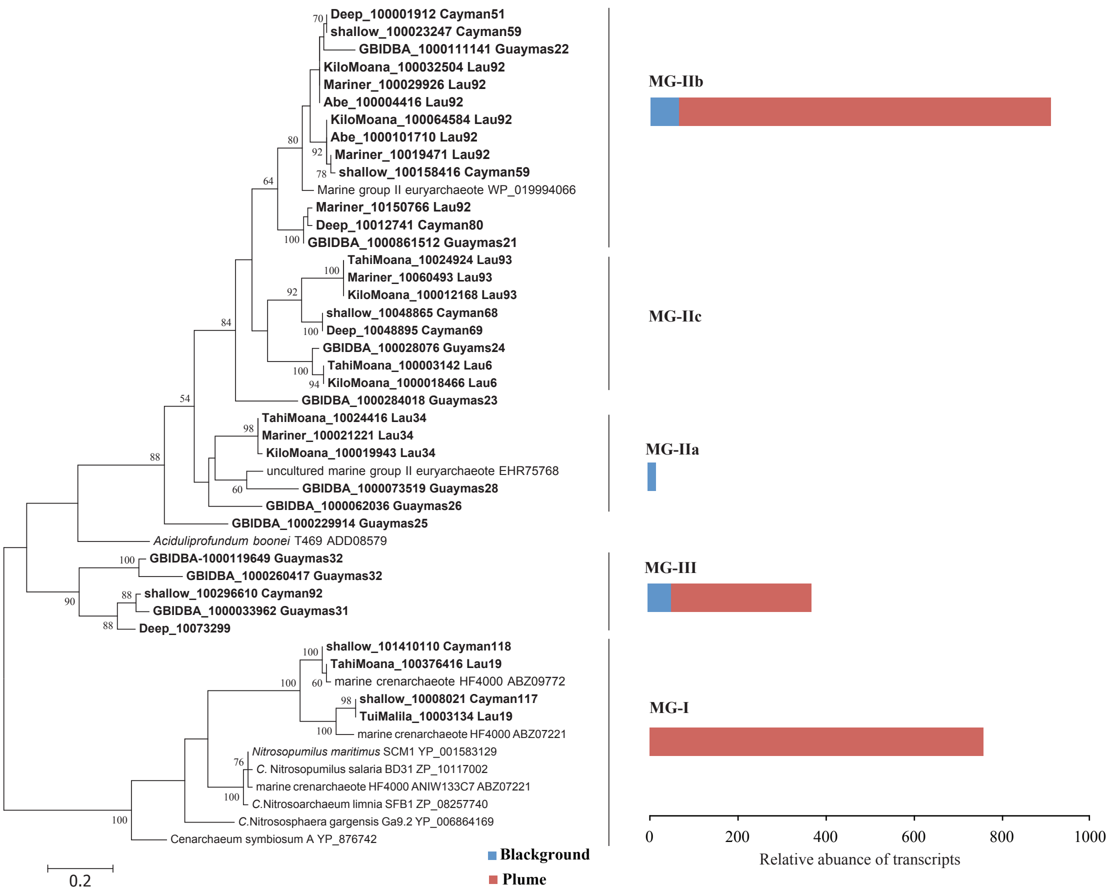

Supplementary Figure 12. Phylogenetic analysis of geranylgeranylglyceryl phosphate synthase (GGGPS) coding genes and their expression.

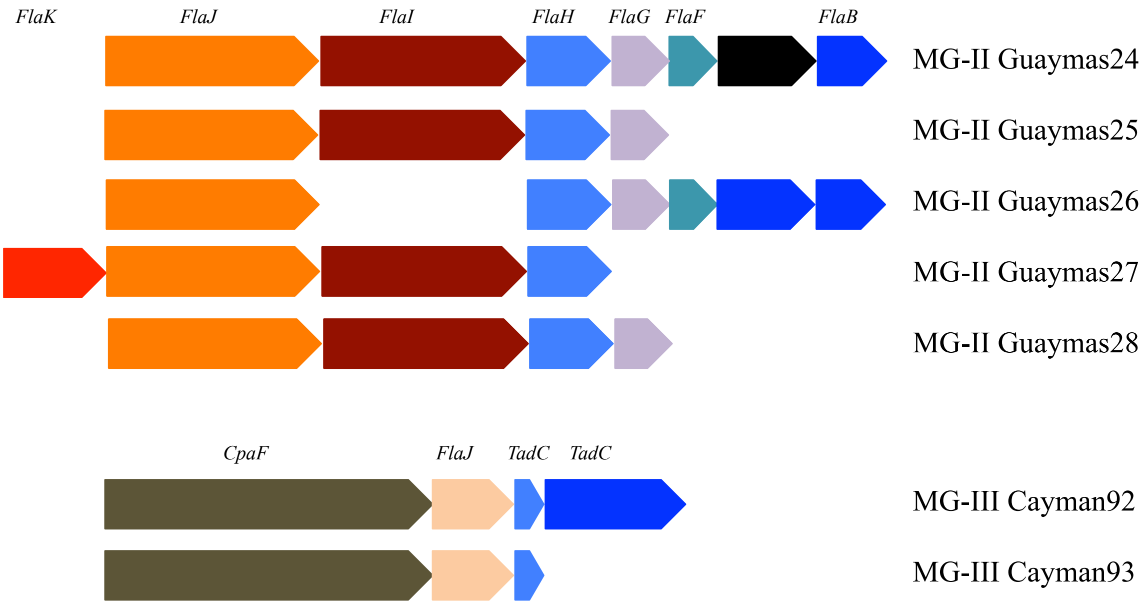

Supplementary Figure 13. Gene cluster for flagellar protein in deep-sea archaea genomes.

Supplementary Tables

Supplementary Table 1. Sample information of this study.

Site	Vent	Sample ID	Sample type	Latitude/Longitude	Depth (m)	Filter size (µm)	No. of sequence reads	DNA or cDNA	Date (DD/MM/YYYY)
Guaymas Basin	Guaymas	GOC11-1#2	Neutrally buoyant plume	N 27°30.95 W 111°25.5	1,993	0.2	171,620,910	DNA	11/07/2004
	Guaymas	GOC12-27a#1	Neutrally buoyant plume	N 27°30.360 W 111°20.818	1,950	0.2	206,157,516	cDNA	11/07/2004
	Guaymas	GOC12-8#12	Above plume background	N 27°29.174 W 111°21.844	1,600	0.2	244,519,176	cDNA	11/07/2004
Cayman Shallow	Von Dam	CTD06_SUPR46	Rising plume	N 18°22.582 W 81°47.885	2,040	0.2	341,285,378	DNA	18/01/2012
	Von Dam	CTD06_SUPR47	Rising plume	N 18°22.58 W 81°47.884	2,238	0.2	318,714,354	DNA	18/01/2012
	Background	CTD11_SUPR60	Shallow background	N 18°43.001 W 81°46.999	2,294	0.2	291,819,402	DNA	26/01//2012
	Von Dam	CTD06_SUPR45	Rising plume	N 18°22.55629 W 81°47.79187	2,042	0.2	21,996,797	cDNA	18/01/2012
	Von Dam	CTD06_SUPR46	Rising plume	N 18°22.582 W 81°47.885	2,040	0.2	12,072,079	cDNA	18/01/2012
	Von Dam	CTD06_SUPR47	Rising plume	N 18°22.58 W 81°47.884	2,238	0.2	83,077,428	cDNA	18/01/2012
	Von Dam	CTD06_SUPR48	Rising plume	N 18°22.583 W 81°47.878	2,254	0.2	30,816,790	cDNA	18/01/2012
	Background	CTD11_SUPR60	Shallow background	N 18°43.001 W 81°46.999	2,202	0.2	111,970,712	cDNA	26/01//2012
	Background	J2-614_SUPR04	Near bottom background	N 18°37.424 W 81°79.695	2,291	0.2	15,605,729	cDNA	13/01/2012
	Background	J2-614_SUPR07	Near bottom background	N 18°37.844 W 81°79.904	2,291	0.2	97,171,498	cDNA	13/01/2012
Cayman Deep	Beebe	CTD08_SUPR50	Rising plume	N 18°32.7901 W 81°43.094	4869	0.2	374,769,904	DNA	21/01/2012
	Beebe	CTD08_SUPR51	Rising plume	N 18°32.7935 W 81°43.1048	4,946	0.2	364,625,048	DNA	21/01/2012
	Background	CTD11_SUPR62	Deep background	N 18°43.001 W 81°46.999	1,953	0.2	267,710,556	DNA	26/01//2012
	Beebe	CTD08_SUPR49	Rising plume	N 18°32.798 W 81°43.096	4,316	0.2	7,076,188	cDNA	21/01/2012
	Beebe	CTD08_SUPR50	Rising plume	N 18°32.7901 W 81°43.094	4,869	0.2	67,250,461	cDNA	21/01/2012
	Beebe	CTD08_SUPR51	Rising plume	N 18°32.7935 W 81°43.1048	4,946	0.2	60,821,416	cDNA	21/01/2012
	Background	CTD11_SUPR62	Deep background	N 18°43.001 W 81°46.999	1,953	0.2	32,468,931	cDNA	26/01//2012
	Background	CTD11_SUPR61	Deep background	N 18°43.001 W 81°46.999	4,850	0.2	16,097,840	cDNA	26/01//2012
Lau Basin Abe	Abe/A1	TN235-J2426-20	Rising plume	S 20 45.672883 W 176 11.434418	1,960	0.8	168,325,584	DNA	25/05/2009

	Abe	TN236-J2449-2	Near bottom background	S 22 45.677706 W 176 11.369574	2,155	0.8	169,488,288	DNA	04/07/2009
	Abe/A1	TN235-J2426-7	Rising plume	S 20 45.672883 W 176 11.434418	2,159	0.8	181,094,744	DNA	25/05/2009
Lau Basin Kilo Moana	Kilo Moana/KM1	TN236-J2436-16	Rising plume	S 20 3.229502 W 176 8.015363	2,605	0.8	174,530,426	DNA	15/06/2009
	Kilo Moana/KM4	TN235-J2424-20	Rising plume	S 20 3.234200 W 176 8.008000	2,440	0.8	157,276,514	DNA	22/05/2009
	Kilo Moana/KM4	TN235-J2424-8	Rising plume	S 20 3.234200 W 176 8.008000	2,639	0.8	118,751,402	DNA	22/05/2009
	Kilo Moana/KM1	TN236-CTD-KM-IP2	Neutrally buoyant plume	S 20 3.246489 W 176 8.011308	2,315	0.2	188,964,668	DNA	13/06/2009
Lau Basin Mariner	Mariner/MA3	TN236-J2440-18	Rising Plume	S 22 10.818293 W 176 36.086423	1,890	0.8	185,135,248	DNA	20/06/2009
	Mariner	TN236-CTD-Mar-BP1	Below plume background	S 20 10.8035165 W 176 36.074496	1,785	0.2	154,673,072	DNA	19/06/2009
Lau Basin Tahi Moana	Tahi Moana	TN236-J2445-20.2	Above plume background	S 20 40.927843 W 176 11.001806	800	0.2	181,482,188	DNA	29/06/2009
	Tahi Moana 1a/SP2	TN236-J2450-9	Rising plume	S 20 40.894100 W 176 10.940463	2,229	0.8	186,087,990	DNA	05/07/2009
Lau Basin Tui Malia	Tui Malila/TM1	TN236-J2447-10	Rising plume	S 21 59.401181 W 176 34.124651	1,919	0.8	187,067,650	DNA	01/07/2009

Supplementary Table 2. Summary of metagenomic *de novo* assembly in this study

Site	Name of genomic assembly	Total size of assembly (bp)	No. of scaffolds	Protein coding genes	rRNA genes	tRNA genes	COG clusters	Pfam clusters
Guaymas Basin	GB	572,189,614	449,452	944,447	1,811	9,684	4,673	14,576
Cayman	Deep	218,214,362	130,227	310,600	717	3,531	4,386	12,423
	Shallow	424,665,720	326,156	656,792	1,677	6,819	4,584	13,870
Lau Basin	Abe	429,147,887	170,673	401,957	294	4,218	4,456	13,850
	KM	317,764,985	106,034	664,114	188	8,039	4,443	14,587
	Mariner	160,783,924	55,478	202,932	186	2,322	4,203	12,115
	Tahi	213,649,834	66,057	267,200	181	2,933	4,336	13,038
	Tui	107,945,955	35,532	141,590	94	1,614	3,771	10,753

Supplementary Table 3. Information on Archaea genomic bins identified from metagenomes of three hydrothermal vent sites. CDs, coding sequences. N, none identified.

Site	ESOM bin	No. of scaffolds	Genes	CDs	Size (bp)	Average GC (%)	Coverage	No. of genomes	Genome completeness	16S rRNA	23S rRNA	Taxonomy
Guaymas Basin	Guaymas69	378	3,943	3,807	3,000,321	28.33	60.8	2	0.53	N	N	MGI
	Guaymas96	131	1,563	1,480	1,086,991	35.30	18.2	2	0.52	N	N	MGI
	Guaymas21	69	1,432	1,367	1,501,106	56.28	123.8	1	0.85	Scaffold_436	Scaffold_268 Scaffold_7335	MGII
	Guaymas29	68	1,578	1,524	1,597,417	43.81	64.7	1	0.84	Scaffold_4201	Scaffold_667 Scaffold_1558	MGII
	Guaymas23	50	1,484	1,425	1,633,324	49.67	57.4	1	0.84	Scaffold_6	Scaffold_77 Scaffold _666	MGII
	Guaymas22	42	1,381	1,322	1,448,805	55.78	15.8	1	0.78	Scaffold_1054	Scaffold_3568 Scaffold_1680	MGII
	Guaymas24	107	2,159	2,067	2,402,520	44.03	29.0	2	0.79	Scaffold_951	Scaffold_9661	MGII
	Guaymas25	77	1,946	1,886	2,275,685	43.83	34.9	2	0.91	Scaffold_931<br>Scaffold_1742	Scaffold_114 Scaffold_938	MGII
	Guaymas26	31	1,467	1,401	1,751,774	43.36	20.2	1	0.76	Scaffold_1827	Scaffold_2530 Scaffold_65	MGII
	Guaymas27	125	1,135	1,100	1,026,877	41.71	61.2	1	0.48	N	Scaffold_16284<br>Scaffold_14647	MGII
	Guaymas28	81	1,764	1,702	1,921,720	44.88	12.7	1	0.89	Scaffold_908	Scaffold_2540 Scaffold_1321	MGII
	Guaymas34	29	219	213	181,895	59.60	8.0	1	0.07	N	N	MGII
	Guaymas31	38	1,268	1,213	1,235,045	62.89	14.1	1	0.85	Scaffold_5840	Scaffold_827	MGIII
	Guaymas32	108	2,550	2,437	2,392,464	36.85	26.0	2	0.83	Scaffold_1157<br>Scaffold_201<br>Scaffold_1821	Scaffold_868 Scaffold_9321<br>Scaffold_1291 Scaffold_5965	MGIII
	Guaymas33	44	596	538	418,682	29.93	8.4	1	0.48	Scaffold_1531	Scaffold_1531	Parvarchaeota
	Guaymas35	66	1,152	1,085	836,653	33.72	12.2	2	0.56	N	Scaffold_25602	putative DHVEG-6
Cayman Shallow	Cayman117	191	2,158	2,024	1,486,040	35.06	30.9	2	0.60	Scaffold_13711<br>Scaffold_30009	N	MGI
	Cayman118	77	719	672	533,720	33.94	19.1	1	0.49	N	N	MGI
	Cayman59	177	2,509	2,406	2,600,802	59.86	27.8	2	0.83	Scaffold_923<br>Scaffold_12733	N	MGII

	Cayman68	220	1,395	1362	1,381,458	61.49	21.5	1	0.69	N	N	MGII
	Cayman92	100	827	793	702,020	65.41	31.8	1	0.65	N	N	MGIII
Cayman Deep	Cayman91	120	1,064	994	680,344	35.62	229.0	1	0.23	N	N	MGI
	Cayman51	23	1,353	1,294	1,479,632	59.42	68.4	1	0.83	Scaffold_214	N	MGII
	Cayman80	115	1,290	1,235	1,322,386	56.06	32.1	1	0.68	Scaffold_5359	N	MGII
	Cayman69	181	1,126	1,095	1,100,332	62.21	47.1	1	0.51	N	N	MGII
	Cayman93	88	619	597	541,227	64.41	73.7	1	0.46	N	N	MGIII
Lau Basin	Lau19	2,100	20,732	19759	15,219,040	33.50	319.3	10	0.77	Abe_scaffold_6793<br>Mariner_scaffold_8696<br>Tahi_Moana_scaffold_6459<br>Tui_Malila_scaffold_9553	Kilo_Moana_scaffold_11328<br>Tui_Malila_scaffold_9553	MGI
	Lau6	298	6,159	5,919	7,417,145	44.630	29.5	4	0.68	Abe_scaffold_10380<br>Kilo_Moana_scaffold_516<br>Tahi_Moana_scaffold_148	Kilo_Moana_scaffold_11<br>Tahi_Moana_scaffold_57<br>Tahi_Moana_scaffold_128	MGII
	Lau34	297	2,658	2579	3,085,883	52.33	18.4	3	0.37	N	Mariner_scaffold_1596	MGII
	Lau92	252	2,856	2,764	2,780,614	57.76	27.9	4	0.31	Kilo_Moana_scaffold_3617<br>Mariner_scaffold_1345	Mariner_scaffold_389<br>Mariner_scaffold_4239	MGII
	Lau93	76	621	602	623,523	55.18	15.5	2	0.15	N	N	MGII
	Lau125	51	474	435	337,928	30.21	16.2	2	0.10	N	N	Parvarchaeota

Supplementary Table 4. Identification of single copy conserved genes in each archaea genome bins

Annotation	PFAM-ID	Guaymas69	Guaymas96	Guaymas21	Guaymas29	Guaymas23	Guaymas22	Guaymas24	Guaymas25	Guaymas26	Guaymas27	Guaymas28	Cayman34	Guaymas31	Guaymas32	Guaymas33	Guaymas35	Cayman51	Cayman69	Cayman80	Cayman91	Cayman93	Cayman59	Cayman68	Cayman92	Cayman117	Cayman118	Guaymas_30	Lau6	Lau19	Lau34	Lau92	Lau93	Lau125
Enolase, C0terminal TIM barrel domain	pfam00113	0	0	1	1	1	1	1	1	1	1	1	0	1	2	0	0	1	0	1	0	0	2	0	0	0	1	0	4	1	1	2	0	0
Triosephosphate isomerase	pfam00121	0	2	1	1	1	1	1	1	1	1	1	0	1	2	0	0	1	1	1	0	0	2	0	0	1	2	4	3	10	0	4	0	0
ATP synthase, subunit c	pfam00137	2	3	1	1	1	1	1	2	0	1	1	1	1	2	0	0	1	0	1	0	0	2	1	1	1	2	0	4	10	1	0	0	0
Phosphoglycerate kinase	pfam00162	0	0	0	0	0	0	0	0	0	0	0	0	0	0	0	0	1	2	1	0	0	0	1	1	2	0	0	3	7	0	1	0	0
Ribosomal protein S12	pfam00164	0	2	1	1	1	0	1	1	1	1	1	0	1	2	0	0	0	0	0	0	0	0	0	0	0	0	0	2	0	0	0	0	0
Ribosomal protein S7p/S5e	pfam00177	0	1	1	1	1	2	1	1	1	0	1	0	0	2	0	0	1	1	1	0	0	2	1	1	1	1	0	2	11	0	0	0	0
Ribosomal protein L2, RNA binding domain	pfam00181	0	2	1	1	1	1	1	1	1	0	1	0	2	2	0	0	1	0	1	0	0	2	0	0	0	1	1	4	10	1	1	0	0
Ribosomal protein S3, C0terminal domain	pfam00189	0	1	0	0	1	1	1	1	1	0	1	1	1	2	1	2	1	0	0	0	0	2	0	1	3	1	0	3	10	2	1	0	1
Ribosomal protein S19	pfam00203	0	0	1	1	1	0	0	0	0	0	0	0	1	0	0	0	0	1	0	0	1	0	0	1	0	0	0	0	4	0	0	0	0
Ribosomal protein L22p/L17e	pfam00237	2	0	1	1	1	1	2	2	0	1	1	0	1	1	0	0	1	0	1	0	0	2	2	0	2	0	0	4	10	1	2	0	0
Ribosomal protein L14p/L23e	pfam00238	1	0	1	1	1	1	2	1	0	0	1	0	1	2	0	0	1	0	1	0	2	2	0	1	1	1	0	3	5	1	1	0	0
Ribosomal protein L16p/L10e	pfam00252	1	3	1	1	1	1	0	1	1	1	1	0	1	2	0	0	1	0	1	0	1	2	0	1	2	0	0	3	10	0	1	0	0
Ribosomal protein S14p/S29e	pfam00253	3	1	1	1	1	1	1	1	1	1	1	0	1	2	0	0	1	1	1	0	1	2	0	0	2	1	0	4	9	3	0	0	0
Ribosomal protein L23	pfam00276	0	2	2	1	1	1	2	1	1	2	1	0	1	2	1	2	1	1	1	0	0	2	2	1	1	1	0	4	10	2	1	0	0
Ribosomal protein L5	pfam00281	1	2	0	1	1	1	1	2	1	0	1	0	1	2	0	0	0	0	0	0	0	0	0	0	0	0	0	0	3	2	0	0	0
Ribosomal protein L3	pfam00297	1	1	0	0	0	0	0	0	0	0	0	0	0	0	1	1	1	0	1	0	1	2	0	1	0	1	0	4	9	2	1	0	0
Ribosomal protein L11, RNA binding domain	pfam00298	0	0	0	0	0	0	0	0	0	0	0	0	0	0	0	1	0	0	0	0	0	0	0	0	0	0	1	0	5	0	0	0	0
Ribosomal protein S15	pfam00312	0	2	1	1	1	1	1	1	2	2	1	0	1	2	1	2	1	0	0	1	1	2	2	1	1	0	0	4	9	3	3	0	0
Ribosomal protein S2	pfam00318	2	1	1	1	1	1	1	1	1	1	1	0	1	2	0	0	1	1	1	0	3	2	0	2	2	0	0	3	10	0	1	0	0
Ribosomal protein L30p/L7e	pfam00327	0	1	1	1	1	1	0	1	1	3	1	0	1	1	0	0	1	1	2	0	0	2	1	0	1	1	1	2	10	1	2	0	0
Ribosomal protein S5,N0terminal domain	pfam00333	1	0	1	1	0	0	1	2	0	0	1	1	0	0	0	0	1	0	0	2	1	1	0	0	2	2	1	4	10	3	0	0	0
Nucleoside diphosphate kinase	pfam00334	0	1	0	0	0	0	0	0	0	0	0	0	1	0	0	0	1	1	0	1	1	1	2	0	1	1	0	0	8	0	0	0	0
SecY translocase	pfam00344	0	1	1	1	1	1	1	1	1	1	1	0	1	2	0	0	1	0	1	1	1	2	0	1	0	0	0	4	5	1	3	0	0
Ribosomal protein L6	pfam00347	0	2	1	1	1	1	1	1	1	1	1	0	1	2	0	0	1	0	1	0	1	2	0	0	2	1	0	3	7	1	3	0	0
Ribosomal protein S17	pfam00366	0	2	1	1	1	1	1	1	1	1	1	0	1	2	0	0	1	0	1	1	1	2	0	0	1	1	0	1	1	1	3	0	0
Hydroxymethylglutaryl0CoA reductase	pfam00368	0	2	2	1	1	1	2	1	1	2	1	0	0	3	0	0	2	0	2	0	1	4	1	0	3	0	1	4	8	1	0	1	0
Ribosomal protein S9/S16	pfam00380	1	0	1	1	1	1	1	1	2	0	1	0	1	2	0	1	1	1	1	0	0	2	1	1	1	2	0	3	6	5	1	0	0
Ribosomal protein S8	pfam00410	1	2	0	0	1	1	1	1	1	1	1	0	1	2	1	2	1	1	1	0	0	2	0	1	2	1	0	3	10	0	2	0	2
Ribosomal protein S11	pfam00411	1	2	1	1	1	1	1	1	1	0	1	0	1	2	1	2	1	1	0	0	1	2	2	1	2	0	0	4	10	3	4	1	1
Ribosomal protein S13/S18	pfam00416	1	2	1	1	1	1	1	1	1	1	1	0	1	2	0	0	1	2	1	0	1	2	2	0	2	0	0	4	10	1	1	0	0
Ribosomal protein L10	pfam00466	1	2	1	1	1	1	1	1	1	1	1	0	1	2	1	2	1	0	1	0	2	2	0	1	2	1	0	3	10	0	2	0	1
RNA polymerase Ppb2, domain 6	pfam00562	2	2	0	0	0	0	0	1	1	0	1	0	1	1	1	2	1	0	1	0	2	2	0	1	1	0	0	3	10	0	2	0	1

Ribosomal protein L13	pfam00572	0	1	1	1	1	1	1	1	1	1	1	1	1	2	1	2	1	0	1	0	1	2	0	0	2	0	0	4	8	0	2	0	0
Ribosomal protein L4/L1 family	pfam00573	0	1	1	1	1	1	1	1	1	1	1	0	1	2	1	2	1	0	1	0	0	2	0	0	0	1	0	3	0	0	2	0	1
RNA polymerase Rpb1, domain 2	pfam00623	1	1	0	0	0	0	0	0	0	0	0	0	0	0	0	0	0	0	0	0	0	0	0	1	2	0	0	0	10	0	0	0	0
S0adenosyl0L0homocysteine hydrolase, NAD binding domain	pfam00670	2	1	1	1	1	1	2	1	0	1	1	0	1	2	0	2	1	1	1	0	0	2	2	0	1	0	0	4	8	0	1	0	0
Ribosomal L5P family C0terminus	pfam00673	0	0	1	1	1	1	1	1	1	0	1	0	1	2	1	2	1	0	1	0	1	2	0	1	0	0	0	4	4	1	0	0	0
Elongation factor G C0terminus	pfam00679	0	1	1	1	1	1	2	1	1	0	1	0	1	3	1	2	1	1	1	0	0	2	1	1	1	0	0	2	6	0	1	2	0
Ribosomal protein L1p/10e family	pfam00687	1	2	1	1	1	1	2	1	1	0	1	0	2	2	1	2	1	1	0	0	0	2	1	1	2	0	0	2	10	0	1	2	0
Adenylosuccinate synthase	pfam00709	0	1	1	1	1	1	1	1	1	1	1	0	1	2	0	1	1	1	1	0	1	2	1	1	0	0	0	4	4	0	2	0	0
EF01 guanine nucleotide exchange domain	pfam00736	2	0	1	1	1	1	2	1	1	0	1	0	1	2	1	2	1	1	1	0	1	2	0	1	2	1	0	3	7	0	1	0	0
tRNA synthetases class I (E and Q), catalytic domain	pfam00749	0	1	1	1	1	1	0	1	1	0	1	1	1	2	1	1	1	0	0	0	0	2	0	1	2	1	0	3	7	1	1	0	0
tRNA synthetases class I (R)	pfam00750	3	0	1	1	1	1	2	1	1	0	1	0	1	2	1	2	1	1	1	0	1	2	0	1	2	1	0	2	6	0	1	0	0
XPG N0terminal domain	pfam00752	0	1	1	1	1	1	2	1	1	0	1	0	1	2	1	2	1	0	1	0	0	3	0	1	1	1	0	2	5	0	1	2	0
Ribosomal protein L15	pfam00827	2	1	1	1	1	1	1	1	1	0	1	0	1	2	1	2	1	1	0	0	1	2	1	0	0	0	0	4	6	3	4	1	0
Ribosomal protein L29	pfam00831	1	2	1	2	1	1	2	0	1	0	0	0	1	2	1	1	1	1	1	0	0	2	1	2	2	0	0	2	10	0	1	1	0
Ribosomal protein L39	pfam00832	0	3	1	1	1	1	1	2	1	1	1	0	1	3	0	1	1	1	1	0	0	2	3	1	2	0	0	4	10	4	4	1	1
Ribosomal protein S17	pfam00833	1	2	0	0	0	0	0	0	1	0	0	0	0	0	0	0	1	1	0	0	0	1	1	0	0	0	0	0	6	0	4	0	0
Ribosomal protein L18p/L5e family	pfam00861	0	1	1	1	1	1	1	1	1	1	1	0	1	2	1	2	1	0	1	1	0	2	1	1	1	1	0	3	10	0	0	0	0
XPGI0region	pfam00867	2	0	1	1	1	1	2	1	1	0	1	0	1	3	1	2	1	2	1	0	1	2	0	1	2	1	0	3	8	0	1	0	0
Ribosomal protein S4e	pfam00900	1	2	1	1	1	1	1	1	1	1	1	0	1	2	0	2	1	1	1	0	1	1	0	1	1	1	0	4	10	1	2	0	0
Uncharacterized protein family UPF0004	pfam00919	2	0	1	1	2	2	1	1	1	1	1	0	1	2	0	2	1	1	1	0	0	2	0	0	1	0	0	4	8	1	2	0	0
Ribosomal protein L44	pfam00935	0	1	1	1	1	1	1	1	1	0	1	0	0	2	1	2	0	0	0	0	0	0	1	1	0	1	2	0	1	1	0	0	0
GMP synthase C terminal domain	pfam00958	0	0	0	0	0	0	0	0	0	0	0	0	1	0	1	0	1	1	1	0	0	2	0	0	1	1	0	4	9	2	1	0	0
RNA polymerase Rpb3/RpoA insert domain	pfam01000	1	2	1	1	1	1	1	1	1	0	1	0	1	2	1	2	1	1	0	0	0	2	1	0	1	1	0	4	10	3	4	1	0
Ribosomal protein S3Ae family	pfam01015	2	2	1	1	1	1	1	1	1	0	1	0	1	2	1	2	1	2	0	0	0	2	1	0	2	0	0	4	8	3	4	1	0
60O0methylguanie DAN methyltransferase, DNA binding domain	pfam01035	1	0	1	1	1	1	1	1	1	1	1	0	1	2	2	1	1	1	0	0	1	2	2	1	1	1	0	4	6	1	1	0	0
Ribosomal protein S19e	pfam01090	1	2	1	1	1	1	1	1	1	1	1	0	1	2	1	1	1	0	0	0	1	2	1	1	1	0	0	4	9	3	1	1	2
Ribosomal protein S6e	pfam01092	1	2	1	1	1	1	1	1	1	1	1	0	1	2	1	1	1	0	0	0	1	2	1	1	2	0	0	4	7	3	1	1	2
Uncharacterized protein family UPF0027	pfam01139	1	1	1	1	0	1	1	0	0	1	0	0	1	2	1	3	1	0	1	0	0	2	1	1	1	1	0	3	10	0	0	1	0
Ribosomal protein L21e	pfam01157	0	1	1	1	1	1	1	1	1	0	1	1	1	2	1	1	1	0	0	0	0	2	0	1	2	1	0	3	9	1	1	0	0
Shwachman0Bodian0Diamond syndrome (SBDS) protein	pfam01172	1	1	0	0	1	0	0	0	0	0	0	0	1	2	1	2	1	1	1	0	0	2	0	1	1	2	0	2	10	3	4	0	0
SNO glutamine amidotransferase family	pfam01174	0	0	1	0	0	0	1	1	0	1	1	0	1	0	1	2	1	0	1	0	0	1	0	0	1	0	0	2	5	0	0	2	0
Dihydroorotate dehydrogenase	pfam01180	0	1	0	1	1	1	1	0	1	0	0	0	0	2	0	0	0	0	0	0	0	1	1	1	0	0	0	0	0	0	1	0	0
RNA polymerase Rpb5, C0terminal domain	pfam01191	0	1	1	1	1	1	1	1	1	1	1	0	1	2	1	2	1	1	1	0	0	2	1	1	1	1	0	4	7	2	3	0	0
RNA polymerase Rpb6	pfam01192	0	2	1	1	1	1	1	1	1	0	1	0	1	2	1	2	1	1	1	0	0	2	1	1	1	0	0	4	10	2	4	1	0
RNA polymerase N	pfam01194	0	2	1	1	1	1	1	1	1	1	1	0	1	2	1	1	1	0	1	1	0	2	0	0	1	2	0	1	10	3	0	0	2
Ribosomal protein L31e	pfam01198	2	2	1	1	1	1	2	1	0	1	1	0	1	2	0	2	1	1	1	0	0	2	2	0	1	0	0	4	8	0	1	0	0
Ribosomal protein S28e	pfam01200	2	1	1	1	1	1	1	2	1	1	1	0	1	3	2	3	2	0	2	0	1	5	2	1	2	2	1	4	10	2	4	0	2
Ribosomal protein S8e	pfam01201	0	0	0	0	0	0	1	0	0	0	0	0	0	1	0	0	0	0	0	0	0	0	0	0	0	0	1	0	0	0	1	0	0
Ribosomal protein L24e	pfam01246	0	0	0	1	0	0	0	0	0	0	0	0	1	2	0	0	0	0	0	0	0	0	0	0	0	0	1	0	0	0	0	0	0
Translation initiation factor SUI1	pfam01253	2	2	1	1	1	1	1	1	1	1	1	0	1	3	0	2	1	1	1	0	1	1	0	1	1	0	1	4	6	1	2	0	0

Fibrillarin	pfam01269	1	0	1	1	1	1	2	1	1	1	1	0	1	2	2	1	1	0	1	0	1	2	1	1	1	0	1	4	6	3	2	1	2
Ribosomal protein L19e	pfam01280	1	1	1	1	1	1	1	1	1	1	1	0	1	2	1	1	1	0	0	0	1	2	1	1	1	0	0	4	6	3	1	1	2
Ribosomal protein S24e	pfam01282	1	1	1	1	1	1	1	1	1	1	1	0	1	2	0	1	1	1	1	0	1	2	1	1	0	0	0	4	4	0	2	0	0
Eukaryotic elongation factor 5A hypusine, DNA0binding OB fold	pfam01287	0	0	1	1	1	1	2	1	1	0	1	0	1	2	1	1	1	0	1	0	0	2	1	1	3	0	2	4	7	1	4	0	0
Ribonuclease HII	pfam01351	2	0	2	0	1	0	1	1	1	1	2	0	1	2	1	1	1	1	2	1	1	2	0	1	1	0	2	3	10	3	2	0	0
V0type APTase 116kDa subunit family	pfam01496	0	0	1	1	1	1	2	1	0	1	1	0	1	2	0	2	1	1	1	0	0	2	2	0	1	0	1	4	5	0	1	0	0
TruB family pseudouridylate synthase (N terminal domain)	pfam01509	0	1	1	0	0	0	1	1	1	0	0	0	0	1	1	2	1	0	1	0	0	3	0	1	2	1	0	2	10	0	2	2	0
Ribosomal protein S27a	pfam01599	1	1	1	1	1	1	1	0	0	0	1	0	1	1	0	0	0	0	0	0	0	0	0	0	0	0	0	0	1	0	0	0	0
Ribosomal protein L32	pfam01655	0	2	1	1	1	1	1	1	1	1	1	0	1	2	0	0	1	0	0	1	0	2	0	0	1	1	0	1	10	3	0	0	0
Ribosomal protein S27	pfam01667	2	2	1	1	1	1	2	1	1	0	1	0	1	2	1	2	1	1	1	2	1	2	0	1	1	1	0	3	10	0	1	0	0
Hami family	pfam01725	1	0	1	1	1	1	1	2	0	0	1	0	1	2	0	2	1	0	1	1	0	2	0	0	1	0	0	4	10	3	4	1	0
Ribosomal protein L37ae protein family	pfam01780	1	1	1	1	1	1	1	2	0	0	1	0	1	2	0	2	1	0	2	0	1	2	0	1	1	0	0	5	10	3	4	0	0
Putative snoRNA binding domain	pfam01798	2	1	1	1	1	1	2	1	1	1	1	0	1	2	1	2	1	1	1	0	1	2	1	1	2	0	1	4	10	3	4	1	2
ATP synthase subunit D	pfam01813	1	1	2	1	1	1	1	1	1	1	2	0	1	3	1	4	1	1	1	0	0	2	1	1	1	0	0	3	7	2	4	1	0
NAC domain	pfam01849	0	0	0	0	0	0	0	0	0	0	0	0	0	0	0	0	0	0	1	0	0	0	0	0	0	0	0	0	1	0	0	0	0
Putative integral membrane protein DUF46	pfam01864	0	1	1	1	1	1	1	1	1	1	1	0	1	2	1	2	1	0	1	1	0	2	1	1	2	1	0	3	10	0	0	1	0
Putativee diphthamide synthesis protein	pfam01866	1	0	0	0	0	0	0	0	0	0	0	0	0	0	0	0	0	0	1	0	0	0	0	0	0	0	0	0	1	1	0	0	0
Domain of unknown function UPF0086	pfam01868	0	1	2	1	1	1	2	1	1	0	1	1	1	2	1	2	1	1	1	0	1	2	2	0	3	1	0	3	10	2	2	0	0
PcrB family	pfam01884	0	2	1	1	1	1	1	1	1	0	1	0	1	2	1	2	1	0	1	0	0	2	1	0	1	1	1	3	10	3	0	1	2
Eukaryotic and archaeal DNA primase small subunit	pfam01896	2	1	1	1	1	1	0	1	1	1	1	0	1	2	0	2	1	0	0	0	1	2	0	0	3	1	0	4	10	0	4	0	0
Ribosomal protein L37e	pfam01907	2	2	1	1	1	1	2	2	0	0	1	0	1	2	1	2	1	0	1	1	1	2	1	1	1	0	0	2	10	1	2	0	0
Translation initiation factor 6 (eIF06)	pfam01912	1	2	1	1	1	1	1	1	1	1	1	0	1	2	2	1	1	0	0	0	0	2	2	1	1	2	2	4	10	2	1	0	0
Prefoldin subunit	pfam01920	1	1	0	0	0	0	0	0	0	0	0	0	1	2	1	0	0	0	0	1	1	0	0	1	2	1	0	0	10	0	0	0	2
SRP19 protein	pfam01922	0	0	1	1	1	1	1	1	1	0	1	0	1	0	0	0	1	0	0	0	1	2	1	1	0	0	0	4	0	0	1	0	0
S0adenosylmethionine synthetase (AdoMet synthetase)	pfam01941	1	0	1	1	1	1	1	1	1	0	1	1	1	0	0	0	1	0	1	0	1	2	1	0	0	0	1	4	0	3	1	0	0
Aspartate carbamoyltransferase regulatory chain, allosteric domain	pfam01948	2	1	1	1	1	1	1	2	0	0	2	0	1	2	0	2	1	0	0	1	1	1	1	1	2	0	0	0	10	2	3	0	0
Protein of unknown function DUF99	pfam01949	1	2	1	1	1	1	1	1	1	0	1	0	2	2	0	0	1	1	0	0	1	1	1	1	3	2	0	2	10	3	0	0	0
Archease protein family (MTH1598/TM1083)	pfam01951	1	1	1	0	1	1	2	1	1	1	2	0	1	2	1	3	1	1	1	0	0	1	2	1	1	1	0	4	9	3	2	1	1
Molybdenum cofactor biosynthesis enzyme	pfam01967	0	2	1	1	1	1	2	1	2	1	1	0	1	2	1	2	1	1	2	0	0	2	2	1	2	0	0	4	7	2	4	2	0
Protein of unknown function DUF115	pfam01973	2	0	1	1	1	1	1	1	1	0	1	0	1	2	0	2	0	0	0	1	0	1	0	0	2	0	4	0	1	0	0	0	0
Peptidyl0tRNA hydrolase PTH2	pfam01981	0	0	0	0	0	0	0	0	0	0	0	0	0	0	0	0	1	1	1	0	0	1	0	1	0	1	0	3	10	2	0	0	0
Domain of unknown function DUF120	pfam01982	1	2	1	1	1	1	0	1	0	0	1	0	1	2	0	0	1	0	0	0	1	2	1	0	2	0	0	4	10	1	4	0	0
Double stranded DNA binding domain	pfam01984	3	2	0	0	0	0	1	1	1	0	1	0	1	0	0	0	0	1	0	0	0	0	1	0	3	1	0	4	10	3	0	1	0
ATP synthase (F/14 kDa) subunit	pfam01990	0	0	1	0	1	1	1	1	1	1	1	0	1	2	0	0	1	2	1	1	1	2	1	1	1	1	0	2	6	3	4	2	0
ATP synthase (E/31 kDa) subunit	pfam01991	2	1	1	1	1	1	1	1	1	1	1	1	1	3	0	0	1	2	1	2	0	2	1	1	1	2	1	4	10	2	1	0	0
N2,N20dimethylguanosine tRNA methyltransferase	pfam02005	2	1	1	1	1	1	1	1	1	0	1	0	1	2	0	2	1	1	1	0	0	3	1	2	0	1	1	4	10	1	1	0	0
Protein of unknown function DUF137	pfam02006	1	1	0	0	0	0	0	0	0	0	0	0	1	2	1	2	0	0	0	2	0	0	0	1	2	1	0	0	9	0	0	0	0
Aspartate carbamoyltransferase regulatory	pfam02748	1	1	1	1	1	1	1	1	1	1	1	0	1	2	0	4	1	0	1	0	0	2	0	1	2	1	0	4	10	1	2	0	0

chain, metal binding domain																																		
LigT like Phosphoesterase	pfam02834	1	0	1	1	1	1	1	1	1	0	1	0	1	2	0	2	1	1	1	0	0	2	1	1	0	1	0	4	2	3	0	0	0
GAD domain	pfam02938	0	1	2	1	1	1	2	1	1	1	1	0	1	2	0	1	1	1	1	0	1	2	1	1	0	0	0	4	4	0	2	0	0
Signal peptide binding domain	pfam02978	2	2	1	1	1	1	1	2	1	1	1	0	1	2	0	0	1	2	0	0	0	2	2	0	1	0	0	4	8	3	2	0	0
Prefoldin subunit	pfam02996	1	1	1	1	1	1	1	1	1	1	1	0	1	2	0	0	1	0	0	0	0	2	1	0	0	1	0	4	2	1	0	0	0
Early transcription elongation factor of RNA pol II, NGN section	pfam03439	0	1	0	0	0	0	0	0	0	0	0	0	0	0	0	2	0	0	0	0	0	0	0	0	3	0	0	0	10	1	0	0	0
tRNA synthetases B5 damain	pfam03484	2	2	1	1	1	1	1	1	1	1	1	0	1	2	0	0	1	2	0	0	0	2	1	1	2	1	0	4	10	3	1	0	0
Ribosomal protein S5, C0terminal domain	pfam03719	0	0	1	1	1	1	1	1	1	0	1	0	1	1	0	0	1	1	1	3	0	2	2	0	2	0	0	4	10	2	1	1	0
Elongation factors G, domain IV	pfam03764	2	2	1	1	1	1	1	1	1	1	1	0	1	2	0	2	1	1	1	0	0	2	1	1	0	0	0	4	0	2	3	0	0
YjeF0related protein N0terminus	pfam03853	1	0	0	1	0	0	0	0	1	1	0	0	1	2	0	0	1	0	1	0	1	2	1	1	2	0	0	4	5	1	1	0	0
RNA polymerase Rpb4	pfam03874	0	0	1	1	1	1	1	1	1	1	1	0	1	0	0	0	1	0	1	1	0	2	1	0	1	1	0	3	4	1	0	1	0
SHS2 domain found in N terminus of Rpb7p/Rpc25p/MJ0397	pfam03876	0	1	0	1	0	1	0	0	0	0	1	0	1	0	1	2	0	1	0	0	0	0	1	0	1	0	0	0	3	0	0	0	0
Sec61beta family	pfam03911	1	3	2	1	2	1	2	2	2	2	2	0	1	4	0	0	2	1	2	1	3	4	2	1	3	3	0	4	10	2	7	1	0
Ribosomal protein L11, N0terminal domain	pfam03946	2	0	1	1	1	1	1	1	0	0	1	1	2	2	0	0	1	0	1	2	1	2	2	1	2	0	0	3	10	2	8	1	0
Ribosomal protein L2, C0terminal domain	pfam03947	0	1	0	0	0	0	0	0	0	0	1	0	0	0	0	0	1	0	1	0	1	2	2	1	3	0	0	4	10	1	4	0	1
tRNA synthetases class I (E and Q), anti0codon binding domain	pfam03950	1	0	0	0	0	0	0	0	0	0	0	0	0	0	1	0	0	0	0	0	0	0	0	0	0	1	0	0	0	0	0	0	0
Protein of unknown function (DUF357)	pfam04010	2	1	1	1	1	1	1	1	1	1	1	0	1	3	0	0	1	0	1	0	1	2	1	1	1	1	0	4	11	2	1	0	0
Protein of unknown function (DUF359)	pfam04019	0	2	1	1	1	1	1	1	1	0	1	0	1	2	0	0	1	1	0	0	1	1	1	2	1	2	1	2	10	3	0	0	0
Eukaryotic0type DNA primase, large subunit	pfam04104	2	2	1	1	1	1	1	1	1	1	1	0	1	2	1	2	0	1	0	0	0	0	0	0	0	0	0	0	0	0	0	1	0
DNA / pantothenate metabolism flavoprotein	pfam04127	0	0	0	0	0	0	0	0	0	0	1	0	0	0	0	0	1	0	1	0	0	2	2	1	1	0	0	4	7	2	4	0	1
Nucleolar RNA0binding protein, Nop10p family	pfam04135	0	2	1	1	1	1	1	1	1	0	1	0	1	2	1	2	1	1	1	0	0	2	1	1	1	0	0	4	12	2	4	1	0
Type IIB DNA topoisomerase	pfam04406	1	0	1	0	1	1	1	1	1	0	1	0	1	2	0	0	0	0	0	0	0	1	0	1	2	1	0	0	6	0	0	0	0
D0aminoacyl0tRNA deacylase	pfam04414	0	0	0	0	0	0	0	0	0	0	0	0	0	0	0	2	0	0	0	0	0	0	0	0	0	0	0	0	0	0	0	0	0
RNA polymerase Rpb2, domain 7	pfam04560	0	0	1	1	1	1	2	1	1	0	1	0	1	2	1	2	0	0	0	0	0	0	0	0	0	1	0	0	0	0	0	0	0
RNA polymerase Rpb2, domain 2	pfam04561	0	1	0	0	0	0	0	0	0	0	0	0	0	0	0	0	0	0	0	0	0	0	0	0	0	2	0	0	0	0	0	0	0
RNA polymerase beta subunit	pfam04563	1	0	0	0	0	0	0	0	0	0	1	0	0	0	0	0	0	0	0	0	0	0	0	0	0	0	0	0	4	0	0	0	0
RNA polymerase Rpb2, domain 3	pfam04565	1	0	1	1	1	1	1	2	1	0	1	0	1	2	1	0	1	1	1	0	2	2	0	1	0	0	3	4	1	2	1	2	0
RNA polymerase Rpb2, domain 4	pfam04566	0	1	2	1	2	2	3	2	2	0	2	0	2	2	1	4	1	0	1	0	0	2	1	1	1	0	1	3	10	0	4	2	0
RNA polymerase Rpb2, domain 5	pfam04567	0	2	1	1	1	1	2	0	1	0	1	0	1	2	0	2	0	0	0	0	0	0	0	0	0	0	0	0	0	0	0	0	0
Protein of unknown function, DUF655	pfam04919	1	1	1	1	1	1	2	1	1	0	1	0	1	2	0	2	0	0	0	0	0	0	0	0	0	0	0	0	0	0	0	0	0
NMD3 family	pfam04981	0	1	1	1	1	1	2	1	1	0	1	0	1	2	1	2	1	1	1	1	0	2	1	1	2	0	0	2	5	0	1	1	0
RNA polymerase Rpb1, domain 3	pfam04983	2	0	1	1	1	1	2	1	1	0	1	0	1	2	1	2	1	0	1	0	1	2	0	1	2	0	0	3	5	0	1	0	0
RNA polymerase Rpb1, domain 1	pfam04997	2	1	1	1	1	1	2	1	1	0	1	0	1	2	1	2	1	1	1	0	1	2	0	1	1	0	0	3	5	0	1	0	0
RNA polymerase Rpb1, domain 4	pfam05000	2	1	1	1	1	1	2	1	1	0	1	0	1	2	0	2	1	1	1	0	1	2	0	1	1	0	0	3	5	0	1	0	0
S0adenosyl0L0homocysteine hydrolase	pfam05221	0	2	1	1	1	1	1	2	1	0	1	0	0	2	1	2	1	0	1	0	1	2	2	0	3	1	1	4	10	5	4	1	1
Domain of unknown function (DUF814)	pfam05670	0	0	0	1	1	1	1	1	1	1	1	0	1	2	0	1	1	1	1	0	0	1	2	0	0	0	0	3	0	3	2	1	0
DALR anticodon binding domain	pfam05746	2	1	0	0	0	0	0	0	0	0	0	0	0	0	0	0	0	0	0	0	0	0	0	0	0	1	0	0	10	0	0	0	0
Ribose 50phosphate isomerase A	pfam06026	3	0	1	1	1	1	1	1	1	1	1	0	1	3	0	0	1	0	1	0	1	1	0	0	1	0	0	4	10	3	1	0	0
Spt4/RpoE2 zinc finger	pfam06093	0	1	2	0	1	0	1	1	1	1	2	0	0	2	0	2	1	1	1	2	2	3	1	2	3	1	1	4	10	2	2	0	0
CTP synthase N0terminus	pfam06418	1	1	1	1	0	1	1	1	1	1	1	0	1	2	1	0	1	1	0	0	0	2	1	0	3	0	0	3	10	1	1	0	0

Molybdenum Cofactor Synthesis C	pfam06463	0	0	0	0	1	0	0	0	0	0	0	0	0	0	0	1	1	1	1	0	0	2	1	1	1	0	0	3	5	0	1	1	0
Eukaryotic translation initiation factor 2 alpha subunit	pfam07541	1	0	1	0	0	0	1	0	1	0	0	0	1	1	0	1	1	0	1	0	0	2	1	1	0	0	0	3	0	0	1	0	0
DKCLD (NUC011) domain	pfam08068	2	2	1	1	1	1	1	1	1	1	1	1	1	2	0	0	1	0	0	0	0	1	1	1	1	0	0	1	15	4	2	0	0
Ribosomal S13/S15, N0terminal domain	pfam08069	2	0	0	1	1	1	1	1	1	0	1	0	1	0	1	2	1	0	1	0	1	2	0	1	1	0	0	3	5	0	1	0	0
RS4NT (NUC023) domain	pfam08071	2	0	1	1	1	1	2	1	1	0	1	0	1	3	1	2	1	0	1	0	1	2	0	1	2	0	0	3	5	0	1	0	0
Wyosine base formation	pfam08608	2	1	1	1	1	1	2	1	1	0	1	0	1	2	1	2	1	1	1	0	1	2	1	1	1	0	0	3	5	0	1	0	0
tRNA methyltransferase complex GCD14 subunit	pfam08704	2	1	0	1	0	0	2	1	1	0	1	0	1	2	1	2	0	1	0	0	0	1	1	1	2	0	0	0	6	1	1	0	0
Initiation factor eIF2 gamma, C terminal	pfam09173	0	1	1	2	1	1	2	1	1	0	1	0	1	2	1	2	1	1	1	1	0	2	1	1	3	0	0	2	3	0	1	1	0
Topoisomerase VI B subunit, transducer	pfam09239	2	0	1	1	1	1	2	1	1	0	1	0	1	2	0	2	1	1	1	0	1	2	0	1	0	0	0	3	0	0	1	0	0
tRNA nucleotidyltransferase, second domain	pfam09249	2	1	1	1	0	1	2	1	1	1	1	0	1	2	1	1	0	0	0	0	0	0	0	0	0	0	0	0	0	0	0	0	0
SBDS protein C0terminal domain	pfam09377	0	2	1	1	1	1	1	1	0	1	0	0	0	2	0	0	1	0	1	0	1	2	0	1	2	0	0	3	7	1	2	0	0
Plug domain of Sec61p	pfam10559	0	0	0	0	0	0	0	0	0	0	0	0	0	0	1	0	1	1	1	0	0	2	1	1	1	0	0	3	5	0	1	0	0
Translation initiation factor 2	pfam11987	1	0	0	0	0	0	0	1	0	0	0	0	0	0	1	0	1	0	1	0	1	2	0	1	1	1	0	3	6	0	1	0	0

04

Supplementary Table 5. Peptidase genes with extracellular transport signals (identified with SignalP 4.1, PSORTb version 3.0.2 or PRED-SIGNAL) aligned to the MEROPs database.

Taxonomy	Bin	Gene	MEPS_ID	Family	Subfamily	E-value	Background*	Plume*	MEPS annotation
MG-I	Cayman117	shallow_10118261	MER160056	M01	M01.UPW	7E-129	0.00	7.13	family M1 unassigned peptidases
MG-I	Cayman117	shallow_10035205	MER344017	M22	M22.UPW	1E-142	0.00	0.00	family M22 unassigned peptidases
MG-I	Cayman117	shallow_10034864	MER161432	S08A	S08.097	0	0.00	6.86	peptidase C1
MG-I	Cayman117	shallow_10045724	MER162654	S08A	S08.UPA	0	0.00	0.00	subfamily S8A unassigned peptidases
MG-I	Cayman118	shallow_10070857	MER344010	M22	M22.UPW	6E-102	0.00	0.00	family M22 unassigned peptidases
MG-I	Cayman118	shallow_10081562	MER173096	S26A	S26.UPA	2E-15	0.00	30.87	subfamily S26A unassigned peptidases
MG-I	Cayman91	Deep_10069111	MER160210	M67B	M67.010	8E-21	0.00	0.00	JAMM-like protein
MG-I	Guaymas69	GBIDBA_100132187	MER344010	M22	M22.UPW	1E-148	234.19	74.10	family M22 unassigned peptidases
MG-I	Guaymas69	GBIDBA_100253934	MER175136	S08A	S08.104	0	49.21	0.00	AF70 peptidase
MG-I	Guaymas96	GBIDBA_100062286	MER162591	S08A	S08.104	0	0.00	0.00	AF70 peptidase
MG-I	Guaymas96	GBIDBA_100121738	MER267556	S26B	S26.UPB	4E-28	65.40	51.74	subfamily S26B unassigned peptidases
MG-I	Lau19	Abe_100032812	MER344017	M22	M22.UPW	2E-142	-	-	family M22 unassigned peptidases
MG-I	Lau19	Abe_100069075	MER344017	M22	M22.UPW	2E-139	-	-	family M22 unassigned peptidases
MG-I	Lau19	KiloMoana_100056035	MER161334	M22	M22.UPW	5E-92	-	-	family M22 unassigned peptidases
MG-I	Lau19	Mariner_10014285	MER344017	M22	M22.UPW	2E-142	-	-	family M22 unassigned peptidases
MG-I	Lau19	Mariner_10072061	MER344017	M22	M22.UPW	3E-63	-	-	family M22 unassigned peptidases
MG-I	Lau19	TahiMoana_100348211	MER344017	M22	M22.UPW	2E-142	-	-	family M22 unassigned peptidases
MG-I	Lau19	TuiMalila_10010998	MER344017	M22	M22.UPW	2E-142	-	-	family M22 unassigned peptidases
MG-I	Lau19	TuiMalila_10027305	MER344017	M22	M22.UPW	8E-139	-	-	family M22 unassigned peptidases
MG-I	Lau19	Mariner_10006386	MER160065	M67B	M67.010	1E-13	-	-	JAMM-like protein
MG-I	Lau19	Abe_100027619	MER161432	S08A	S08.097	2E-157	-	-	peptidase C1
MG-I	Lau19	KiloMoana_100093884	MER161432	S08A	S08.097	9E-88	-	-	peptidase C1
MG-I	Lau19	KiloMoana_100093885	MER161432	S08A	S08.097	4E-65	-	-	peptidase C1
MG-I	Lau19	Mariner_10053172	MER161432	S08A	S08.097	0	-	-	peptidase C1
MG-I	Lau19	Mariner_100720617	MER161432	S08A	S08.097	0	-	-	peptidase C1
MG-I	Lau19	TahiMoana_10065823	MER161432	S08A	S08.097	0	-	-	peptidase C1
MG-I	Lau19	TuiMalila_10026305	MER161432	S08A	S08.097	0	-	-	peptidase C1
MG-I	Lau19	TuiMalila_10042841	MER161432	S08A	S08.097	0	-	-	peptidase C1
MG-I	Lau19	Abe_100082244	MER162591	S08A	S08.104	0	-	-	AF70 peptidase
MG-I	Lau19	KiloMoana_100088854	MER162591	S08A	S08.104	0	-	-	AF70 peptidase
MG-I	Lau19	TahiMoana_10043084	MER162591	S08A	S08.104	0	-	-	AF70 peptidase
MG-I	Lau19	TuiMalila_10026548	MER162591	S08A	S08.104	0	-	-	AF70 peptidase
MG-I	Lau19	Abe_1000389615	MER162654	S08A	S08.UPA	0	-	-	subfamily S8A unassigned peptidases
MG-I	Lau19	KiloMoana_100053584	MER162654	S08A	S08.UPA	0	-	-	subfamily S8A unassigned peptidases
MG-I	Lau19	KiloMoana_100225212	MER162654	S08A	S08.UPA	1E-150	-	-	subfamily S8A unassigned peptidases
MG-I	Lau19	Mariner_10062712	MER162654	S08A	S08.UPA	0	-	-	subfamily S8A unassigned peptidases
MG-I	Lau19	TahiMoana_10049034	MER162654	S08A	S08.UPA	0	-	-	subfamily S8A unassigned peptidases
MG-II	Cayman51	Deep_1000059109	MER331724	M06	M06.UPW	7E-21	0.00	6.92	family M6 unassigned peptidases
MG-II	Cayman51	Deep_1000019160	MER382133	M14A	M14.007	2E-20	18.96	25.76	carboxypeptidase T

MG-II	Cayman51	Deep_100006714	MER242589	M14A	M14.UNA	7E-82	0.00	20.28	subfamily M14A non-peptidase homologues
MG-II	Cayman51	Deep_10003526	MER242589	M14A	M14.UNA	2E-24	0.00	27.59	subfamily M14A non-peptidase homologues
MG-II	Cayman51	Deep_100005967	MER173423	M22	M22.UPW	9E-179	0.00	13.45	family M22 unassigned peptidases
MG-II	Cayman51	Deep_100010359	MER195385	M28A	M28.UPA	8E-52	0.00	36.71	subfamily M28A unassigned peptidases
MG-II	Cayman51	Deep_10012275	MER195385	M28A	M28.UPA	2E-50	0.00	23.61	subfamily M28A unassigned peptidases
MG-II	Cayman51	Deep_100019541	MER196242	M28B	M28.UPB	1E-33	0.00	0.00	subfamily M28B unassigned peptidases
MG-II	Cayman51	Deep_100006344	MER162347	M30	M30.UPW	3E-103	0.00	23.18	family M30 unassigned peptidases
MG-II	Cayman51	Deep_1000019174	MER203517	S08A	S08.UPA	1E-66	8.89	51.35	subfamily S8A unassigned peptidases
MG-II	Cayman51	Deep_10002577	MER016991	S08A	S08.123	5E-61	0.00	17.05	KP-43 peptidase
MG-II	Cayman51	Deep_100021563	MER159986	S08A	S08.UPA	0	0.00	57.31	subfamily S8A unassigned peptidases
MG-II	Cayman51	Deep_100006333	MER054569	S08A	S08.UPA	2E-42	16.80	51.36	subfamily S8A unassigned peptidases
MG-II	Cayman51	Deep_100005839	MER145048	S08A	S08.UPA	5E-34	0.00	42.67	subfamily S8A unassigned peptidases
MG-II	Cayman51	Deep_1000019206	MER198352	S26B	S26.UPB	2E-14	0.00	48.75	subfamily S26B unassigned peptidases
MG-II	Cayman59	shallow_100067312	MER331724	M06	M06.UPW	2E-21	0.00	0.00	family M6 unassigned peptidases
MG-II	Cayman59	shallow_10041915	MER331724	M06	M06.UPW	4E-20	0.00	0.00	family M6 unassigned peptidases
MG-II	Cayman59	shallow_10015034	MER242589	M14A	M14.UNA	7E-82	10.23	2214.77	subfamily M14A non-peptidase homologues
MG-II	Cayman59	shallow_100035913	MER382133	M14A	M14.007	2E-20	0.00	6.93	carboxypeptidase T
MG-II	Cayman59	shallow_10018714	MER242589	M14A	M14.UNA	2E-82	0.00	0.00	subfamily M14A non-peptidase homologues
MG-II	Cayman59	shallow_100246610	MER242588	M14A	M14.UPA	7E-21	0.00	0.00	subfamily M14A unassigned peptidases
MG-II	Cayman59	shallow_10024346	MER242589	M14A	M14.UNA	2E-24	0.00	9.89	subfamily M14A non-peptidase homologues
MG-II	Cayman59	shallow_100098613	MER242589	M14A	M14.UNA	6E-23	0.00	0.00	subfamily M14A non-peptidase homologues
MG-II	Cayman59	shallow_10011381	MER111823	M14X	M14.UNW	6E-14	65.11	444.61	family M14 non-peptidase homologues
MG-II	Cayman59	shallow_100033425	MER173423	M22	M22.UPW	9E-179	0.00	28.94	family M22 unassigned peptidases
MG-II	Cayman59	shallow_100142412	MER173423	M22	M22.UPW	2E-148	0.00	0.00	family M22 unassigned peptidases
MG-II	Cayman59	shallow_10119392	MER195385	M28A	M28.UPA	8E-52	64.77	9.87	subfamily M28A unassigned peptidases
MG-II	Cayman59	shallow_10105312	MER195385	M28A	M28.UPA	2E-48	23.13	0.00	subfamily M28A unassigned peptidases
MG-II	Cayman59	shallow_100023269	MER195385	M28A	M28.UPA	2E-50	0.00	8.47	subfamily M28A unassigned peptidases
MG-II	Cayman59	shallow_100009838	MER196242	M28B	M28.UPB	1E-33	0.00	81.89	subfamily M28B unassigned peptidases
MG-II	Cayman59	shallow_10029871	MER196242	M28B	M28.UPB	2E-34	0.00	50.28	subfamily M28B unassigned peptidases
MG-II	Cayman59	shallow_100119723	MER162347	M30	M30.UPW	4E-112	0.00	21.37	family M30 unassigned peptidases
MG-II	Cayman59	shallow_10011679	MER162347	M30	M30.UPW	3E-103	0.00	17.81	family M30 unassigned peptidases
MG-II	Cayman59	shallow_100035927	MER203517	S08A	S08.UPA	1E-66	0.00	42.24	subfamily S8A unassigned peptidases
MG-II	Cayman59	shallow_100034923	MER016985	S08A	S08.123	3E-59	0.00	15.20	KP-43 peptidase
MG-II	Cayman59	shallow_100183610	MER016991	S08A	S08.123	1E-61	17.74	10.33	KP-43 peptidase
MG-II	Cayman59	shallow_10002536	MER016991	S08A	S08.123	5E-61	0.00	3.06	KP-43 peptidase
MG-II	Cayman59	shallow_10051215	MER159986	S08A	S08.UPA	0	0.00	20.55	subfamily S8A unassigned peptidases
MG-II	Cayman59	shallow_100092437	MER159986	S08A	S08.UPA	0	0.00	0.00	subfamily S8A unassigned peptidases
MG-II	Cayman59	shallow_10000982	MER161502	S08A	S08.UPA	3E-69	0.00	10.79	subfamily S8A unassigned peptidases
MG-II	Cayman59	shallow_10047414	MER054569	S08A	S08.UPA	2E-42	5.75	49.11	subfamily S8A unassigned peptidases
MG-II	Cayman59	shallow_100169110	MER054569	S08A	S08.UPA	8E-37	15.76	9.17	subfamily S8A unassigned peptidases
MG-II	Cayman59	shallow_100024230	MER145048	S08A	S08.UPA	5E-34	0.00	11.47	subfamily S8A unassigned peptidases
MG-II	Cayman59	shallow_10053811	MER145048	S08A	S08.UPA	1E-39	8.84	9.43	subfamily S8A unassigned peptidases
MG-II	Cayman59	shallow_100035959	MER198352	S26B	S26.UPB	2E-14	0.00	0.00	subfamily S26B unassigned peptidases
MG-II	Cayman68	shallow_10123122	MER280279	I87	I87.UPW	2E-21	0.00	0.00	family I87 unassigned peptidase inhibitors

MG-II	Cayman68	shallow_100281311	MER280279	I87	I87.UPW	4E-22	0.00	0.00	family I87 unassigned peptidase inhibitors
MG-II	Cayman68	shallow_10098414	MER075049	M06	M06.UPW	1E-18	0.00	9.20	family M6 unassigned peptidases
MG-II	Cayman68	shallow_10048576	MER075049	M06	M06.UPW	7E-19	0.00	0.00	family M6 unassigned peptidases
MG-II	Cayman68	shallow_10072105	MER242589	M14A	M14.UNA	5E-81	281.28	0.00	subfamily M14A non-peptidase homologues
MG-II	Cayman68	shallow_10064602	MER242589	M14A	M14.UNA	6E-24	0.00	0.00	subfamily M14A non-peptidase homologues
MG-II	Cayman68	shallow_10052398	MER343990	M22	M22.UPW	1E-130	0.00	0.00	family M22 unassigned peptidases
MG-II	Cayman68	shallow_10078306	MER343990	M22	M22.UPW	1E-129	0.00	0.00	family M22 unassigned peptidases
MG-II	Cayman68	shallow_10082481	MER182283	M22	M22.UNW	4E-15	81.04	311.29	family M22 non-peptidase homologues
MG-II	Cayman68	shallow_10068431	MER072148	S08A	S08.123	2E-83	35.45	0.00	KP-43 peptidase
MG-II	Cayman68	shallow_10048861	MER085659	S08A	S08.UPA	1E-20	32.82	0.00	subfamily S8A unassigned peptidases
MG-II	Cayman68	shallow_10136651	MER054569	S08A	S08.UPA	4E-20	0.00	12.77	subfamily S8A unassigned peptidases
MG-II	Cayman68	shallow_10018375	MER161502	S08A	S08.UPA	1E-28	0.00	11.24	subfamily S8A unassigned peptidases
MG-II	Cayman68	shallow_10127053	MER019280	S08A	S08.135	1E-44	64.43	0.00	tengconlysin
MG-II	Cayman69	Deep_10027616	MER075049	M06	M06.UPW	6E-19	0.00	5.91	family M6 unassigned peptidases
MG-II	Cayman69	Deep_10026735	MER195385	M28A	M28.UPA	2E-48	0.00	0.00	subfamily M28A unassigned peptidases
MG-II	Cayman69	Deep_10022026	MER162347	M30	M30.UPW	6E-75	0.00	36.37	family M30 unassigned peptidases
MG-II	Cayman69	Deep_10103504	MER159986	S08A	S08.UPA	1E-135	0.00	0.00	subfamily S8A unassigned peptidases
MG-II	Cayman69	Deep_10078491	MER054569	S08A	S08.UPA	1E-23	0.00	0.00	subfamily S8A unassigned peptidases
MG-II	Cayman69	Deep_10059692	MER161420	S15	S15.UPW	7E-101	0.00	0.00	family S15 unassigned peptidases
MG-II	Cayman80	Deep_10018048	MER075049	M06	M06.UPW	4E-17	0.00	20.18	family M6 unassigned peptidases
MG-II	Cayman80	Deep_100114829	MER242588	M14A	M14.UPA	2E-21	0.00	37.42	subfamily M14A unassigned peptidases
MG-II	Cayman80	Deep_10028302	MER242589	M14A	M14.UNA	6E-80	0.00	0.00	subfamily M14A non-peptidase homologues
MG-II	Cayman80	Deep_100261714	MER194154	M28A	M28.UPA	3E-11	0.00	0.00	subfamily M28A unassigned peptidases
MG-II	Cayman80	Deep_10014534	MER195385	M28A	M28.UPA	1E-49	0.00	9.18	subfamily M28A unassigned peptidases
MG-II	Cayman80	Deep_100117110	MER195385	M28A	M28.UPA	3E-48	0.00	0.00	subfamily M28A unassigned peptidases
MG-II	Cayman80	Deep_100146329	MER196242	M28B	M28.UPB	4E-36	0.00	0.00	subfamily M28B unassigned peptidases
MG-II	Cayman80	Deep_10009995	MER162347	M30	M30.UPW	8E-89	0.00	3.31	family M30 unassigned peptidases
MG-II	Cayman80	Deep_10010933	MER016985	S08A	S08.123	1E-70	0.00	33.34	KP-43 peptidase
MG-II	Cayman80	Deep_100066617	MER054569	S08A	S08.UPA	0	0.00	0.00	subfamily S8A unassigned peptidases
MG-II	Cayman80	Deep_10052722	MER054569	S08A	S08.UPA	4E-33	0.00	11.32	subfamily S8A unassigned peptidases
MG-II	Cayman80	Deep_10043696	MER019280	S08A	S08.135	2E-44	0.00	20.30	tengconlysin
MG-II	Cayman80	Deep_100095614	MER161420	S15	S15.UPW	3E-119	0.00	29.39	family S15 unassigned peptidases
MG-II	Guaymas21	GBIDBA_1000026916	MER075049	M06	M06.UPW	5E-17	45.56	126.15	family M6 unassigned peptidases
MG-II	Guaymas21	GBIDBA_1000328911	MER242589	M14A	M14.UNA	2E-80	98.68	303.05	subfamily M14A non-peptidase homologues
MG-II	Guaymas21	GBIDBA_1000539420	MER242588	M14A	M14.UPA	1E-21	154.88	250.61	subfamily M14A unassigned peptidases
MG-II	Guaymas21	GBIDBA_1000133238	MER173423	M22	M22.UPW	1E-129	197.54	216.37	family M22 unassigned peptidases
MG-II	Guaymas21	GBIDBA_100016701	MER194154	M28A	M28.UPA	3E-11	95.76	90.90	subfamily M28A unassigned peptidases
MG-II	Guaymas21	GBIDBA_100022889	MER195385	M28A	M28.UPA	3E-48	115.93	253.96	subfamily M28A unassigned peptidases
MG-II	Guaymas21	GBIDBA_100029251	MER196242	M28B	M28.UPB	4E-36	259.96	594.08	subfamily M28B unassigned peptidases
MG-II	Guaymas21	GBIDBA_1000050726	MER162347	M30	M30.UPW	4E-89	93.45	328.20	family M30 unassigned peptidases
MG-II	Guaymas21	GBIDBA_100003259	MER016985	S08A	S08.123	3E-70	80.38	259.75	KP-43 peptidase
MG-II	Guaymas21	GBIDBA_100013961	MER016991	S08A	S08.123	3E-55	107.03	200.12	KP-43 peptidase
MG-II	Guaymas21	GBIDBA_1000043762	MER054569	S08A	S08.UPA	0	163.58	431.33	subfamily S8A unassigned peptidases
MG-II	Guaymas21	GBIDBA_1000073716	MER161502	S08A	S08.UPA	8E-108	105.27	867.81	subfamily S8A unassigned peptidases

MG-II	Guaymas21	GBIDBA_1000050714	MER054569	S08A	S08.UPA	6E-31	109.78	438.00	subfamily S8A unassigned peptidases
MG-II	Guaymas21	GBIDBA_100007269	MER019280	S08A	S08.135	6E-45	110.94	381.28	tengconlysin
MG-II	Guaymas21	GBIDBA_1000234026	MER145048	S08A	S08.UPA	3E-36	82.73	210.70	subfamily S8A unassigned peptidases
MG-II	Guaymas21	GBIDBA_1000226331	MER161420	S15	S15.UPW	8E-96	128.97	353.15	family S15 unassigned peptidases
MG-II	Guaymas22	GBIDBA_1000060962	MER075049	M06	M06.UPW	1E-20	0.00	18.75	family M6 unassigned peptidases
MG-II	Guaymas22	GBIDBA_1000200031	MER118947	M14A	M14.UPA	7E-19	21.33	33.74	subfamily M14A unassigned peptidases
MG-II	Guaymas22	GBIDBA_1000040816	MER242589	M14A	M14.UNA	1E-82	11.45	0.00	subfamily M14A non-peptidase homologues
MG-II	Guaymas22	GBIDBA_1000060920	MER173423	M22	M22.UPW	1E-137	14.76	0.00	family M22 unassigned peptidases
MG-II	Guaymas22	GBIDBA_1000074443	MER195385	M28A	M28.UPA	1E-49	31.03	24.54	subfamily M28A unassigned peptidases
MG-II	Guaymas22	GBIDBA_1000111121	MER195385	M28A	M28.UPA	2E-50	35.55	63.27	subfamily M28A unassigned peptidases
MG-II	Guaymas22	GBIDBA_1000106517	MER196242	M28B	M28.UPB	1E-34	14.19	0.00	subfamily M28B unassigned peptidases
MG-II	Guaymas22	GBIDBA_1000054120	MER162347	M30	M30.UPW	3E-101	14.95	106.44	family M30 unassigned peptidases
MG-II	Guaymas22	GBIDBA_100003109	MER016985	S08A	S08.123	6E-60	23.91	40.53	KP-43 peptidase
MG-II	Guaymas22	GBIDBA_1000168126	MER016985	S08A	S08.123	4E-58	0.00	15.22	KP-43 peptidase
MG-II	Guaymas22	GBIDBA_1000105533	MER159986	S08A	S08.UPA	0	7.20	0.00	subfamily S8A unassigned peptidases
MG-II	Guaymas22	GBIDBA_100006813	MER243713	S08A	S08.UPA	5E-56	17.79	28.15	subfamily S8A unassigned peptidases
MG-II	Guaymas22	GBIDBA_100005418	MER054569	S08A	S08.UPA	1E-34	4.44	84.21	subfamily S8A unassigned peptidases
MG-II	Guaymas22	GBIDBA_100051761	MER145048	S08A	S08.UPA	1E-38	16.72	0.00	subfamily S8A unassigned peptidases
MG-II	Guaymas22	GBIDBA_1000042726	MER161420	S15	S15.UPW	4E-118	8.45	0.00	family S15 unassigned peptidases
MG-II	Guaymas22	GBIDBA_1000031040	MER155256	S26B	S26.UPB	5E-14	0.00	135.06	subfamily S26B unassigned peptidases
MG-II	Guaymas23	GBIDBA_1000032651	MER075049	M06	M06.UPW	6E-19	7.81	222.54	family M6 unassigned peptidases
MG-II	Guaymas23	GBIDBA_100005167	MER118947	M14A	M14.UPA	2E-23	0.00	694.06	subfamily M14A unassigned peptidases
MG-II	Guaymas23	GBIDBA_10000007232	MER242589	M14A	M14.UNA	7E-107	32.61	257.94	subfamily M14A non-peptidase homologues
MG-II	Guaymas23	GBIDBA_1000032611	MER173423	M22	M22.UPW	2E-127	15.24	0.00	family M22 unassigned peptidases
MG-II	Guaymas23	GBIDBA_100042594	MER195385	M28A	M28.UPA	3E-54	10.28	170.76	subfamily M28A unassigned peptidases
MG-II	Guaymas23	GBIDBA_100008826	MER195385	M28A	M28.UPA	1E-51	4.51	321.37	subfamily M28A unassigned peptidases
MG-II	Guaymas23	GBIDBA_1000007853	MER196242	M28B	M28.UPB	2E-21	28.49	450.76	subfamily M28B unassigned peptidases
MG-II	Guaymas23	GBIDBA_1000108317	MER162347	M30	M30.UPW	1E-67	11.64	202.55	family M30 unassigned peptidases
MG-II	Guaymas23	GBIDBA_1000070411	MER016985	S08A	S08.123	1E-67	22.25	227.43	KP-43 peptidase
MG-II	Guaymas23	GBIDBA_100000787	MER016985	S08A	S08.123	1E-56	12.88	68.78	KP-43 peptidase
MG-II	Guaymas23	GBIDBA_1000000733	MER054569	S08A	S08.UPA	1E-178	0.00	137.44	subfamily S8A unassigned peptidases
MG-II	Guaymas23	GBIDBA_1000142320	MER161502	S08A	S08.UPA	2E-65	12.70	271.35	subfamily S8A unassigned peptidases
MG-II	Guaymas23	GBIDBA_100010837	MER054569	S08A	S08.UPA	2E-34	0.00	198.10	subfamily S8A unassigned peptidases
MG-II	Guaymas23	GBIDBA_1000001670	MER085875	S08A	S08.UPA	9E-58	20.01	324.10	subfamily S8A unassigned peptidases
MG-II	Guaymas23	GBIDBA_1000001638	MER145048	S08A	S08.UPA	1E-34	18.41	300.92	subfamily S8A unassigned peptidases
MG-II	Guaymas24	GBIDBA_100006592	MER280279	I87	I87.UPW	4E-25	7.27	483.32	family I87 unassigned peptidase inhibitors
MG-II	Guaymas24	GBIDBA_1000111312	MER239341	M06	M06.UPW	7E-18	7.04	33.41	family M6 unassigned peptidases
MG-II	Guaymas24	GBIDBA_100080835	MER116728	M14A	M14.UNA	2E-20	17.70	151.20	subfamily M14A non-peptidase homologues
MG-II	Guaymas24	GBIDBA_100009649	MER242589	M14A	M14.UNA	3E-81	11.56	54.85	subfamily M14A non-peptidase homologues
MG-II	Guaymas24	GBIDBA_1000158220	MER343990	M22	M22.UPW	2E-118	15.20	144.24	family M22 unassigned peptidases
MG-II	Guaymas24	GBIDBA_1000100817	MER195385	M28A	M28.UPA	6E-105	0.00	91.86	subfamily M28A unassigned peptidases
MG-II	Guaymas24	GBIDBA_1000079548	MER191900	M28A	M28.UPA	8E-15	15.06	71.49	subfamily M28A unassigned peptidases
MG-II	Guaymas24	GBIDBA_1000100812	MER195385	M28A	M28.UPA	5E-57	8.84	41.96	subfamily M28A unassigned peptidases
MG-II	Guaymas24	GBIDBA_1000086128	MER196242	M28B	M28.UPB	1E-33	0.00	41.11	subfamily M28B unassigned peptidases

MG-II	Guaymas24	GBIDBA_1000086126	MER004565	S01A	S01.467	2E-12	32.82	62.31	hemolymph proteinase 21
MG-II	Guaymas24	GBIDBA_100190936	MER016985	S08A	S08.123	1E-72	4.60	76.38	KP-43 peptidase
MG-II	Guaymas24	GBIDBA_100028073	MER085659	S08A	S08.UPA	3E-35	6.31	149.76	subfamily S8A unassigned peptidases
MG-II	Guaymas24	GBIDBA_100005735	MER016985	S08A	S08.123	9E-64	3.21	68.65	KP-43 peptidase
MG-II	Guaymas24	GBIDBA_1000132816	MER016991	S08A	S08.123	2E-68	4.71	29.80	KP-43 peptidase
MG-II	Guaymas24	GBIDBA_100009522	MER159986	S08A	S08.UPA	4E-168	0.00	16.15	subfamily S8A unassigned peptidases
MG-II	Guaymas24	GBIDBA_1000239118	MER161502	S08A	S08.UPA	5E-70	0.00	92.52	subfamily S8A unassigned peptidases
MG-II	Guaymas24	GBIDBA_100062888	MER054569	S08A	S08.UPA	2E-25	18.55	0.00	subfamily S8A unassigned peptidases
MG-II	Guaymas24	GBIDBA_1000057335	MER161502	S08A	S08.UPA	1E-31	6.14	0.00	subfamily S8A unassigned peptidases
MG-II	Guaymas24	GBIDBA_100037045	MER019280	S08A	S08.135	1E-49	3.81	108.45	tengconlysin
MG-II	Guaymas24	GBIDBA_100003755	MER114217	S09X	S09.UNW	3E-12	5.93	0.00	family S9 non-peptidase homologues
MG-II	Guaymas25	GBIDBA_100011031	MER089612	M11	M11.UPW	3E-17	4.15	78.75	family M11 unassigned peptidases
MG-II	Guaymas25	GBIDBA_1000041874	MER242589	M14A	M14.UNA	7E-81	0.00	50.20	subfamily M14A non-peptidase homologues
MG-II	Guaymas25	GBIDBA_100018687	MER040020	M14A	M14.007	2E-19	3.53	67.01	carboxypeptidase T
MG-II	Guaymas25	GBIDBA_100004735	MER343990	M22	M22.UPW	3E-130	0.00	0.00	family M22 unassigned peptidases
MG-II	Guaymas25	GBIDBA_1000081837	MER194807	M24B	M24.031	2E-37	0.00	0.00	leucine aminopeptidase (Thermotoga}-type)
MG-II	Guaymas25	GBIDBA_100001155	MER160149	M24B	M24.008	2E-37	0.00	104.16	hyperthermophile prolidase
MG-II	Guaymas25	GBIDBA_1000110314	MER194185	M28A	M28.UPA	1E-10	0.00	28.12	subfamily M28A unassigned peptidases
MG-II	Guaymas25	GBIDBA_1000736410	MER085866	M28A	M28.UNA	5E-31	0.00	59.42	subfamily M28A non-peptidase homologues
MG-II	Guaymas25	GBIDBA_1000183819	MER195385	M28A	M28.UPA	4E-103	37.43	88.83	subfamily M28A unassigned peptidases
MG-II	Guaymas25	GBIDBA_1000202325	MER191900	M28A	M28.UPA	8E-23	23.98	0.00	subfamily M28A unassigned peptidases
MG-II	Guaymas25	GBIDBA_100012141	MER195385	M28A	M28.UPA	4E-14	0.00	57.45	subfamily M28A unassigned peptidases
MG-II	Guaymas25	GBIDBA_100009329	MER196242	M28B	M28.UPB	3E-21	0.00	0.00	subfamily M28B unassigned peptidases
MG-II	Guaymas25	GBIDBA_100073649	MER294630	M30	M30.UPW	3E-110	7.30	121.26	family M30 unassigned peptidases
MG-II	Guaymas25	GBIDBA_1000059272	MER294630	M30	M30.UPW	4E-75	21.95	69.44	family M30 unassigned peptidases
MG-II	Guaymas25	GBIDBA_1000011510	MER050059	S08A	S08.UPA	6E-42	18.57	58.74	subfamily S8A unassigned peptidases
MG-II	Guaymas25	GBIDBA_100009309	MER016991	S08A	S08.123	1E-68	0.00	104.41	KP-43 peptidase
MG-II	Guaymas25	GBIDBA_100017435	MER054569	S08A	S08.UPA	5E-155	14.14	0.00	subfamily S8A unassigned peptidases
MG-II	Guaymas25	GBIDBA_1000093915	MER161502	S08A	S08.UPA	1E-55	6.72	127.57	subfamily S8A unassigned peptidases
MG-II	Guaymas25	GBIDBA_1000034726	MER054569	S08A	S08.UPA	9E-47	9.42	29.82	subfamily S8A unassigned peptidases
MG-II	Guaymas25	GBIDBA_100012144	MER016985	S08A	S08.123	3E-79	0.00	51.24	KP-43 peptidase
MG-II	Guaymas25	GBIDBA_1000123322	MER019280	S08A	S08.135	5E-46	16.17	57.57	tengconlysin
MG-II	Guaymas25	GBIDBA_1000059248	MER161420	S15	S15.UPW	2E-116	12.40	78.49	family S15 unassigned peptidases
MG-II	Guaymas25	GBIDBA_100022991	MER360268	S15	S15.UPW	6E-41	19.04	0.00	family S15 unassigned peptidases
MG-II	Guaymas25	GBIDBA_100003358	MER198352	S26B	S26.UPB	3E-13	15.47	36.72	subfamily S26B unassigned peptidases
MG-II	Guaymas26	GBIDBA_10000358131	MER075049	M06	M06.UPW	4E-17	0.00	0.00	family M6 unassigned peptidases
MG-II	Guaymas26	GBIDBA_1000338712	MER242589	M14A	M14.UNA	1E-80	0.00	0.00	subfamily M14A non-peptidase homologues
MG-II	Guaymas26	GBIDBA_100032385	MER242588	M14A	M14.UPA	1E-19	7.25	189.25	subfamily M14A unassigned peptidases
MG-II	Guaymas26	GBIDBA_1000035875	MER343990	M22	M22.UPW	1E-122	0.00	0.00	family M22 unassigned peptidases
MG-II	Guaymas26	GBIDBA_1000016652	MER195385	M28A	M28.UPA	8E-22	10.99	26.07	subfamily M28A unassigned peptidases
MG-II	Guaymas26	GBIDBA_100007774	MER196242	M28B	M28.UPB	9E-35	0.00	0.00	subfamily M28B unassigned peptidases
MG-II	Guaymas26	GBIDBA_1000045926	MER294630	M30	M30.UPW	4E-91	1.82	17.32	family M30 unassigned peptidases
MG-II	Guaymas26	GBIDBA_1000006627	MER050059	S08A	S08.UPA	2E-43	5.40	115.30	subfamily S8A unassigned peptidases
MG-II	Guaymas26	GBIDBA_1000006673	MER050633	S08A	S08.UPA	3E-51	0.00	43.24	subfamily S8A unassigned peptidases

MG-II	Guaymas26	GBIDBA_1000105115	MER051661	S08A	S08.UPA	2E-59	1.59	15.06	subfamily S8A unassigned peptidases
MG-II	Guaymas26	GBIDBA_1000062033	MER085659	S08A	S08.UPA	8E-32	0.00	0.00	subfamily S8A unassigned peptidases
MG-II	Guaymas26	GBIDBA_1000215636	MER179414	S08A	S08.UPA	6E-164	0.00	160.66	subfamily S8A unassigned peptidases
MG-II	Guaymas26	GBIDBA_1000058824	MER230765	S08A	S08.UPA	8E-58	1.08	10.24	subfamily S8A unassigned peptidases
MG-II	Guaymas26	GBIDBA_10000358149	MER161502	S08A	S08.UPA	2E-57	12.67	300.76	subfamily S8A unassigned peptidases
MG-II	Guaymas26	GBIDBA_1000091216	MER054569	S08A	S08.UPA	1E-39	0.00	78.94	subfamily S8A unassigned peptidases
MG-II	Guaymas26	GBIDBA_1000174214	MER016985	S08A	S08.123	4E-73	6.07	57.65	KP-43 peptidase
MG-II	Guaymas26	GBIDBA_1000031638	MER019280	S08A	S08.135	5E-47	0.00	38.08	tengconlysin
MG-II	Guaymas26	GBIDBA_1000062024	MER161420	S15	S15.UPW	3E-85	8.74	41.46	family S15 unassigned peptidases
MG-II	Guaymas26	GBIDBA_1000143226	MER161420	S15	S15.UPW	3E-117	0.00	0.00	family S15 unassigned peptidases
MG-II	Guaymas27	GBIDBA_1000571312	MER331724	M06	M06.UPW	3E-18	0.00	34.51	family M6 unassigned peptidases
MG-II	Guaymas27	GBIDBA_100139213	MER343990	M22	M22.UPW	4E-141	0.00	148.21	family M22 unassigned peptidases
MG-II	Guaymas27	GBIDBA_1000315511	MER196242	M28B	M28.UPB	3E-45	0.00	0.00	subfamily M28B unassigned peptidases
MG-II	Guaymas27	GBIDBA_100063668	MER016991	S08A	S08.123	7E-61	15.92	170.02	KP-43 peptidase
MG-II	Guaymas27	GBIDBA_100285773	MER161502	S08A	S08.UPA	4E-55	36.68	348.20	subfamily S8A unassigned peptidases
MG-II	Guaymas27	GBIDBA_100054791	MER161502	S08A	S08.UPA	3E-23	15.71	111.84	subfamily S8A unassigned peptidases
MG-II	Guaymas27	GBIDBA_100126889	MER161420	S15	S15.UPW	4E-96	8.47	160.89	family S15 unassigned peptidases
MG-II	Guaymas28	GBIDBA_1000200810	MER162003	M06	M06.UPW	2E-14	11.01	0.00	family M6 unassigned peptidases
MG-II	Guaymas28	GBIDBA_1000033654	MER242589	M14A	M14.UNA	6E-98	11.33	26.88	subfamily M14A non-peptidase homologues
MG-II	Guaymas28	GBIDBA_1000200846	MER343990	M22	M22.UPW	2E-130	0.00	0.00	family M22 unassigned peptidases
MG-II	Guaymas28	GBIDBA_1000177115	MER198755	M24B	M24.008	1E-34	0.00	72.55	hyperthermophile prolidase
MG-II	Guaymas28	GBIDBA_1000106338	MER085866	M28A	M28.UNA	3E-29	0.00	64.29	subfamily M28A non-peptidase homologues
MG-II	Guaymas28	GBIDBA_100007353	MER195385	M28A	M28.UPA	2E-88	19.32	137.53	subfamily M28A unassigned peptidases
MG-II	Guaymas28	GBIDBA_100007356	MER195385	M28A	M28.UPA	1E-52	26.71	21.13	subfamily M28A unassigned peptidases
MG-II	Guaymas28	GBIDBA_100011044	MER195385	M28A	M28.UPA	1E-19	0.00	0.00	subfamily M28A unassigned peptidases
MG-II	Guaymas28	GBIDBA_100003364	MER195385	M28A	M28.UPA	1E-24	0.00	27.93	subfamily M28A unassigned peptidases
MG-II	Guaymas28	GBIDBA_1000081741	MER196242	M28B	M28.UPB	7E-43	0.00	0.00	subfamily M28B unassigned peptidases
MG-II	Guaymas28	GBIDBA_1000106337	MER294630	M30	M30.UPW	5E-87	0.00	0.00	family M30 unassigned peptidases
MG-II	Guaymas28	GBIDBA_100011042	MER016985	S08A	S08.123	3E-67	4.93	62.35	KP-43 peptidase
MG-II	Guaymas28	GBIDBA_1000138510	MER016991	S08A	S08.123	3E-72	3.55	50.53	KP-43 peptidase
MG-II	Guaymas28	GBIDBA_1000132216	MER016991	S08A	S08.123	7E-53	6.47	15.36	KP-43 peptidase
MG-II	Guaymas28	GBIDBA_1000073522	MER085659	S08A	S08.UPA	3E-26	0.00	0.00	subfamily S8A unassigned peptidases
MG-II	Guaymas28	GBIDBA_100009092	MER159986	S08A	S08.UPA	1E-151	0.00	0.00	subfamily S8A unassigned peptidases
MG-II	Guaymas28	GBIDBA_1000254118	MER161502	S08A	S08.UPA	1E-65	0.00	102.12	subfamily S8A unassigned peptidases
MG-II	Guaymas28	GBIDBA_1000103641	MER054569	S08A	S08.UPA	7E-43	1.61	15.31	subfamily S8A unassigned peptidases
MG-II	Guaymas28	GBIDBA_1000126242	MER182372	S08A	S08.UPA	3E-42	8.00	75.92	subfamily S8A unassigned peptidases
MG-II	Guaymas28	GBIDBA_1000073531	MER161420	S15	S15.UPW	5E-93	8.65	102.60	family S15 unassigned peptidases
MG-II	Guaymas28	GBIDBA_1000103617	MER161420	S15	S15.UPW	2E-115	17.23	40.90	family S15 unassigned peptidases
MG-II	Guaymas29	GBIDBA_10000668110	MER284620	M06	M06.UPW	2E-21	34.48	0.00	family M6 unassigned peptidases
MG-II	Guaymas29	GBIDBA_100023113	MER242589	M14A	M14.UNA	1E-81	78.08	344.10	subfamily M14A non-peptidase homologues
MG-II	Guaymas29	GBIDBA_1000066871	MER173423	M22	M22.UPW	8E-123	129.55	325.52	family M22 unassigned peptidases
MG-II	Guaymas29	GBIDBA_1000232026	MER129787	M22	M22.UNW	2E-17	101.98	242.00	family M22 non-peptidase homologues
MG-II	Guaymas29	GBIDBA_100010707	MER195385	M28A	M28.UPA	5E-50	67.47	234.84	subfamily M28A unassigned peptidases
MG-II	Guaymas29	GBIDBA_1000111458	MER196242	M28B	M28.UPB	2E-29	48.02	136.75	subfamily M28B unassigned peptidases

MG-II	Guaymas29	GBIDBA_1000201471	MER016985	S08A	S08.123	3E-69	160.87	146.21	KP-43 peptidase
MG-II	Guaymas29	GBIDBA_100015596	MER016985	S08A	S08.123	2E-54	75.25	136.78	KP-43 peptidase
MG-II	Guaymas29	GBIDBA_1000420219	MER054569	S08A	S08.UPA	3E-173	121.86	666.04	subfamily S8A unassigned peptidases
MG-II	Guaymas29	GBIDBA_1000299641	MER243713	S08A	S08.UPA	2E-27	66.42	339.48	subfamily S8A unassigned peptidases
MG-II	Guaymas29	GBIDBA_1000091076	MER019280	S08A	S08.135	1E-48	47.10	447.12	tengconlysin
MG-II	Guaymas29	GBIDBA_1000091063	MER145048	S08A	S08.UPA	9E-35	22.23	163.07	subfamily S8A unassigned peptidases
MG-II	Guaymas34	GBIDBA_1000930111	MER242589	M14A	M14.UNA	6E-82	5.80	55.10	subfamily M14A non-peptidase homologues
MG-II	Guaymas34	GBIDBA_1000727512	MER195385	M28A	M28.UPA	1E-10	34.14	0.00	subfamily M28A unassigned peptidases
MG-II	Guaymas34	GBIDBA_100250151	MER203517	S08A	S08.UPA	5E-47	6.35	150.75	subfamily S8A unassigned peptidases
MG-II	Guaymas34	GBIDBA_100072757	MER161420	S15	S15.UPW	7E-105	0.00	38.87	family S15 unassigned peptidases
MG-II	Lau34	KiloMoana_1000326519	MER089612	M11	M11.UPW	4E-19	-	-	family M11 unassigned peptidases
MG-II	Lau34	Mariner_10004953	MER089612	M11	M11.UPW	3E-19	-	-	family M11 unassigned peptidases
MG-II	Lau34	TahiMoana_100070913	MER089612	M11	M11.UPW	4E-19	-	-	family M11 unassigned peptidases
MG-II	Lau34	Mariner_10044864	MER242588	M14A	M14.UPA	6E-19	-	-	subfamily M14A unassigned peptidases
MG-II	Lau34	Mariner_100007940	MER343990	M22	M22.UPW	1E-122	-	-	family M22 unassigned peptidases
MG-II	Lau34	TahiMoana_10092411	MER343990	M22	M22.UPW	5E-126	-	-	family M22 unassigned peptidases
MG-II	Lau34	KiloMoana_1000005147	MER195385	M28A	M28.UPA	2E-55	-	-	subfamily M28A unassigned peptidases
MG-II	Lau34	Mariner_10000835	MER195385	M28A	M28.UPA	2E-55	-	-	subfamily M28A unassigned peptidases
MG-II	Lau34	Mariner_10044932	MER195385	M28A	M28.UPA	6E-52	-	-	subfamily M28A unassigned peptidases
MG-II	Lau34	TahiMoana_100012311	MER195385	M28A	M28.UPA	2E-55	-	-	subfamily M28A unassigned peptidases
MG-II	Lau34	TahiMoana_10007865	MER195385	M28A	M28.UPA	7E-50	-	-	subfamily M28A unassigned peptidases
MG-II	Lau34	Mariner_10001056	MER050059	S08A	S08.UPA	1E-38	-	-	subfamily S8A unassigned peptidases
MG-II	Lau34	TahiMoana_10032502	MER050059	S08A	S08.UPA	1E-38	-	-	subfamily S8A unassigned peptidases
MG-II	Lau34	KiloMoana_100019946	MER085659	S08A	S08.UPA	7E-31	-	-	subfamily S8A unassigned peptidases
MG-II	Lau34	KiloMoana_100057794	MER051661	S08A	S08.UPA	4E-50	-	-	subfamily S8A unassigned peptidases
MG-II	Lau34	Mariner_100021218	MER085659	S08A	S08.UPA	7E-31	-	-	subfamily S8A unassigned peptidases
MG-II	Lau34	Mariner_10010918	MER051661	S08A	S08.UPA	6E-50	-	-	subfamily S8A unassigned peptidases
MG-II	Lau34	Mariner_10068392	MER051661	S08A	S08.UPA	1E-18	-	-	subfamily S8A unassigned peptidases
MG-II	Lau34	TahiMoana_100075614	MER051661	S08A	S08.UPA	2E-47	-	-	subfamily S8A unassigned peptidases
MG-II	Lau34	TahiMoana_10024419	MER085659	S08A	S08.UPA	7E-31	-	-	subfamily S8A unassigned peptidases
MG-II	Lau34	Mariner_10004453	MER054569	S08A	S08.UPA	2E-148	-	-	subfamily S8A unassigned peptidases
MG-II	Lau34	KiloMoana_100012862	MER161502	S08A	S08.UPA	9E-29	-	-	subfamily S8A unassigned peptidases
MG-II	Lau34	KiloMoana_100025752	MER161502	S08A	S08.UPA	8E-57	-	-	subfamily S8A unassigned peptidases
MG-II	Lau34	Mariner_10000495	MER161502	S08A	S08.UPA	9E-29	-	-	subfamily S8A unassigned peptidases
MG-II	Lau34	Mariner_100028410	MER054569	S08A	S08.UPA	6E-40	-	-	subfamily S8A unassigned peptidases
MG-II	Lau34	Mariner_10003614	MER161502	S08A	S08.UPA	6E-57	-	-	subfamily S8A unassigned peptidases
MG-II	Lau34	TahiMoana_10004646	MER161502	S08A	S08.UPA	9E-29	-	-	subfamily S8A unassigned peptidases
MG-II	Lau34	TahiMoana_10010601	MER161502	S08A	S08.UPA	9E-40	-	-	subfamily S8A unassigned peptidases
MG-II	Lau34	Mariner_100002922	MER016985	S08A	S08.123	3E-62	-	-	KP-43 peptidase
MG-II	Lau34	TahiMoana_10012521	MER016985	S08A	S08.123	9E-62	-	-	KP-43 peptidase
MG-II	Lau34	KiloMoana_100075333	MER019280	S08A	S08.135	6E-47	-	-	tengconlysin
MG-II	Lau34	Mariner_10009034	MER019280	S08A	S08.135	6E-47	-	-	tengconlysin
MG-II	Lau34	TahiMoana_10022786	MER019280	S08A	S08.135	6E-47	-	-	tengconlysin
MG-II	Lau34	TahiMoana_10014931	MER241299	S09X	S09.UNW	1E-10	-	-	family S9 non-peptidase homologues

MG-II	Lau34	Mariner_10002128	MER161420	S15	S15.UPW	3E-81	-	-	family S15 unassigned peptidases
MG-II	Lau6	Abe_100056403	MER280279	I87	I87.UPW	1E-25	-	-	family I87 unassigned peptidase inhibitors
MG-II	Lau6	KiloMoana_1000003867	MER280279	I87	I87.UPW	1E-25	-	-	family I87 unassigned peptidase inhibitors
MG-II	Lau6	Mariner_100019415	MER280279	I87	I87.UPW	1E-25	-	-	family I87 unassigned peptidase inhibitors
MG-II	Lau6	TahiMoana_100026823	MER280279	I87	I87.UPW	1E-25	-	-	family I87 unassigned peptidase inhibitors
MG-II	Lau6	Abe_100018366	MER239341	M06	M06.UPW	7E-17	-	-	family M6 unassigned peptidases
MG-II	Lau6	KiloMoana_100000126	MER239341	M06	M06.UPW	7E-17	-	-	family M6 unassigned peptidases
MG-II	Lau6	Mariner_10009167	MER239341	M06	M06.UPW	7E-17	-	-	family M6 unassigned peptidases
MG-II	Lau6	TahiMoana_10001297	MER239341	M06	M06.UPW	7E-17	-	-	family M6 unassigned peptidases
MG-II	Lau6	Abe_100020857	MER242589	M14A	M14.UNA	1E-82	-	-	subfamily M14A non-peptidase homologues
MG-II	Lau6	KiloMoana_1000043612	MER242589	M14A	M14.UNA	1E-82	-	-	subfamily M14A non-peptidase homologues
MG-II	Lau6	Mariner_100002264	MER242589	M14A	M14.UNA	8E-83	-	-	subfamily M14A non-peptidase homologues
MG-II	Lau6	TahiMoana_100002686	MER242589	M14A	M14.UNA	8E-83	-	-	subfamily M14A non-peptidase homologues
MG-II	Lau6	Abe_100118432	MER173423	M22	M22.UPW	3E-117	-	-	family M22 unassigned peptidases
MG-II	Lau6	KiloMoana_1000002418	MER173423	M22	M22.UPW	3E-117	-	-	family M22 unassigned peptidases
MG-II	Lau6	Mariner_10000707	MER173423	M22	M22.UPW	3E-117	-	-	family M22 unassigned peptidases
MG-II	Lau6	TahiMoana_10001267	MER173423	M22	M22.UPW	3E-117	-	-	family M22 unassigned peptidases
MG-II	Lau6	Abe_1000020530	MER195385	M28A	M28.UPA	2E-55	-	-	subfamily M28A unassigned peptidases
MG-II	Lau6	Abe_1000063816	MER196242	M28B	M28.UPB	2E-33	-	-	subfamily M28B unassigned peptidases
MG-II	Lau6	KiloMoana_1000009421	MER196242	M28B	M28.UPB	1E-33	-	-	subfamily M28B unassigned peptidases
MG-II	Lau6	Mariner_100005126	MER196242	M28B	M28.UPB	8E-34	-	-	subfamily M28B unassigned peptidases
MG-II	Lau6	TahiMoana_100008325	MER196242	M28B	M28.UPB	1E-33	-	-	subfamily M28B unassigned peptidases
MG-II	Lau6	Abe_100004632	MER016991	S08A	S08.123	4E-69	-	-	KP-43 peptidase
MG-II	Lau6	Abe_100123201	MER016985	S08A	S08.123	3E-64	-	-	KP-43 peptidase
MG-II	Lau6	KiloMoana_1000001247	MER016985	S08A	S08.123	4E-64	-	-	KP-43 peptidase
MG-II	Lau6	KiloMoana_1000029912	MER016991	S08A	S08.123	4E-69	-	-	KP-43 peptidase
MG-II	Lau6	Mariner_100005427	MER016991	S08A	S08.123	4E-69	-	-	KP-43 peptidase
MG-II	Lau6	TahiMoana_100003218	MER016991	S08A	S08.123	4E-69	-	-	KP-43 peptidase
MG-II	Lau6	KiloMoana_1000018463	MER085659	S08A	S08.UPA	3E-35	-	-	subfamily S8A unassigned peptidases
MG-II	Lau6	Mariner_10008501	MER085659	S08A	S08.UPA	1E-32	-	-	subfamily S8A unassigned peptidases
MG-II	Lau6	TahiMoana_100003145	MER085659	S08A	S08.UPA	3E-35	-	-	subfamily S8A unassigned peptidases
MG-II	Lau6	Abe_100031712	MER159986	S08A	S08.UPA	2E-167	-	-	subfamily S8A unassigned peptidases
MG-II	Lau6	KiloMoana_100005176	MER159986	S08A	S08.UPA	3E-167	-	-	subfamily S8A unassigned peptidases
MG-II	Lau6	Mariner_10005371	MER159986	S08A	S08.UPA	2E-167	-	-	subfamily S8A unassigned peptidases
MG-II	Lau6	TahiMoana_10001498	MER159986	S08A	S08.UPA	3E-167	-	-	subfamily S8A unassigned peptidases
MG-II	Lau6	Abe_100007388	MER161502	S08A	S08.UPA	2E-58	-	-	subfamily S8A unassigned peptidases
MG-II	Lau6	KiloMoana_1000001220	MER161502	S08A	S08.UPA	2E-58	-	-	subfamily S8A unassigned peptidases
MG-II	Lau6	Mariner_100013020	MER161502	S08A	S08.UPA	2E-58	-	-	subfamily S8A unassigned peptidases
MG-II	Lau6	TahiMoana_100012921	MER161502	S08A	S08.UPA	2E-58	-	-	subfamily S8A unassigned peptidases
MG-II	Lau6	Abe_100011121	MER054569	S08A	S08.UPA	3E-39	-	-	subfamily S8A unassigned peptidases
MG-II	Lau6	KiloMoana_1000034311	MER054569	S08A	S08.UPA	7E-39	-	-	subfamily S8A unassigned peptidases
MG-II	Lau6	Mariner_100015911	MER054569	S08A	S08.UPA	7E-39	-	-	subfamily S8A unassigned peptidases
MG-II	Lau6	TahiMoana_100035512	MER054569	S08A	S08.UPA	7E-39	-	-	subfamily S8A unassigned peptidases
MG-II	Lau6	Abe_100003308	MER019280	S08A	S08.135	2E-42	-	-	tengconlysin

MG-II	Lau6	KiloMoana_1000001289	MER019280	S08A	S08.135	2E-42	-	-	tengconlysin
MG-II	Lau6	Mariner_100001448	MER019280	S08A	S08.135	2E-42	-	-	tengconlysin
MG-II	Lau6	TahiMoana_100015822	MER019280	S08A	S08.135	2E-42	-	-	tengconlysin
MG-II	Lau6	Abe_100016183	MER196475	S09X	S09.UNW	1E-14	-	-	family S9 non-peptidase homologues
MG-II	Lau6	KiloMoana_1000000565	MER196475	S09X	S09.UNW	1E-14	-	-	family S9 non-peptidase homologues
MG-II	Lau6	Mariner_10000562	MER196475	S09X	S09.UNW	1E-14	-	-	family S9 non-peptidase homologues
MG-II	Lau6	TahiMoana_100001664	MER196475	S09X	S09.UNW	1E-14	-	-	family S9 non-peptidase homologues
MG-II	Lau92	Abe_100005706	MER075049	M06	M06.UPW	6E-20	-	-	family M6 unassigned peptidases
MG-II	Lau92	Abe_100006454	MER331724	M06	M06.UPW	4E-21	-	-	family M6 unassigned peptidases
MG-II	Lau92	KiloMoana_1000090213	MER075049	M06	M06.UPW	6E-20	-	-	family M6 unassigned peptidases
MG-II	Lau92	KiloMoana_100076974	MER075049	M06	M06.UPW	3E-21	-	-	family M6 unassigned peptidases
MG-II	Lau92	Mariner_100005940	MER075049	M06	M06.UPW	4E-20	-	-	family M6 unassigned peptidases
MG-II	Lau92	Mariner_100042013	MER075049	M06	M06.UPW	3E-21	-	-	family M6 unassigned peptidases
MG-II	Lau92	TahiMoana_100047017	MER075049	M06	M06.UPW	6E-20	-	-	family M6 unassigned peptidases
MG-II	Lau92	Mariner_100011856	MER242589	M14A	M14.UNA	6E-82	-	-	subfamily M14A non-peptidase homologues
MG-II	Lau92	Abe_100147182	MER242588	M14A	M14.UPA	2E-20	-	-	subfamily M14A unassigned peptidases
MG-II	Lau92	KiloMoana_100114214	MER242588	M14A	M14.UPA	1E-20	-	-	subfamily M14A unassigned peptidases
MG-II	Lau92	Abe_1000002865	MER173423	M22	M22.UPW	0	-	-	family M22 unassigned peptidases
MG-II	Lau92	Abe_1000030462	MER173423	M22	M22.UPW	2E-148	-	-	family M22 unassigned peptidases
MG-II	Lau92	KiloMoana_1000036552	MER173423	M22	M22.UPW	0	-	-	family M22 unassigned peptidases
MG-II	Lau92	KiloMoana_1000089014	MER173423	M22	M22.UPW	2E-148	-	-	family M22 unassigned peptidases
MG-II	Lau92	Mariner_100005866	MER173423	M22	M22.UPW	0	-	-	family M22 unassigned peptidases
MG-II	Lau92	Mariner_100032912	MER173423	M22	M22.UPW	2E-148	-	-	family M22 unassigned peptidases
MG-II	Lau92	TahiMoana_100015160	MER173423	M22	M22.UPW	2E-148	-	-	family M22 unassigned peptidases
MG-II	Lau92	TahiMoana_100022444	MER173423	M22	M22.UPW	0	-	-	family M22 unassigned peptidases
MG-II	Lau92	Abe_100104653	MER195385	M28A	M28.UPA	8E-52	-	-	subfamily M28A unassigned peptidases
MG-II	Lau92	Abe_100055768	MER195385	M28A	M28.UPA	5E-51	-	-	subfamily M28A unassigned peptidases
MG-II	Lau92	Mariner_10045132	MER195385	M28A	M28.UPA	6E-48	-	-	subfamily M28A unassigned peptidases
MG-II	Lau92	Mariner_10055995	MER195385	M28A	M28.UPA	3E-60	-	-	subfamily M28A unassigned peptidases
MG-II	Lau92	Mariner_100020015	MER196242	M28B	M28.UPB	7E-35	-	-	subfamily M28B unassigned peptidases
MG-II	Lau92	Mariner_100015413	MER162347	M30	M30.UPW	2E-111	-	-	family M30 unassigned peptidases
MG-II	Lau92	Mariner_10002393	MER016991	S08A	S08.123	9E-62	-	-	KP-43 peptidase
MG-II	Lau92	Mariner_10011471	MER016985	S08A	S08.123	7E-59	-	-	KP-43 peptidase
MG-II	Lau92	Mariner_10003223	MER203517	S08A	S08.UPA	8E-66	-	-	subfamily S8A unassigned peptidases
MG-II	Lau92	Abe_100120594	MER159986	S08A	S08.UPA	0	-	-	subfamily S8A unassigned peptidases
MG-II	Lau92	Abe_100165621	MER159986	S08A	S08.UPA	0	-	-	subfamily S8A unassigned peptidases
MG-II	Lau92	KiloMoana_100095523	MER159986	S08A	S08.UPA	0	-	-	subfamily S8A unassigned peptidases
MG-II	Lau92	KiloMoana_100110584	MER159986	S08A	S08.UPA	0	-	-	subfamily S8A unassigned peptidases
MG-II	Lau92	Mariner_10020354	MER159986	S08A	S08.UPA	0	-	-	subfamily S8A unassigned peptidases
MG-II	Lau92	Mariner_10059596	MER159986	S08A	S08.UPA	0	-	-	subfamily S8A unassigned peptidases
MG-II	Lau92	Mariner_10001542	MER054569	S08A	S08.UPA	5E-36	-	-	subfamily S8A unassigned peptidases
MG-II	Lau92	Mariner_10005281	MER145048	S08A	S08.UPA	1E-39	-	-	subfamily S8A unassigned peptidases
MG-II	Lau92	Mariner_10012048	MER198352	S26B	S26.UPB	2E-13	-	-	subfamily S26B unassigned peptidases
MG-II	Lau92	Mariner_10020522	MER198352	S26B	S26.UPB	3E-14	-	-	subfamily S26B unassigned peptidases

MG-II	Lau93	KiloMoana_1000002648	MER343990	M22	M22.UPW	9E-125	-	-	family M22 unassigned peptidases
MG-II	Lau93	KiloMoana_100012165	MER085659	S08A	S08.UPA	1E-25	-	-	subfamily S8A unassigned peptidases
MG-II	Lau93	KiloMoana_100019208	MER019280	S08A	S08.135	1E-49	-	-	tengconlysin
MG-II	Lau93	Mariner_10049991	MER019280	S08A	S08.135	4E-43	-	-	tengconlysin
MG-II	Lau93	KiloMoana_100030911	MER241299	S09X	S09.UNW	2E-12	-	-	family S9 non-peptidase homologues
MG-III	Cayman92	shallow_10065503	MER291592	C11	C11.UPW	5E-36	0.00	11.12	family C11 unassigned peptidases
MG-III	Cayman92	shallow_100314310	MER234074	I87	I87.UPW	2E-56	0.00	353.57	family I87 unassigned peptidase inhibitors
MG-III	Cayman92	shallow_10144731	MER097192	M03B	M03.UPB	2E-96	28.04	79.78	subfamily M3B unassigned peptidases
MG-III	Cayman92	shallow_10087562	MER284620	M06	M06.UPW	7E-55	0.00	342.76	family M6 unassigned peptidases
MG-III	Cayman92	shallow_10114731	MER284620	M06	M06.UPW	2E-51	0.00	9.32	family M6 unassigned peptidases
MG-III	Cayman92	shallow_100187517	MER308482	M14C	M14.UPC	4E-16	0.00	34.51	subfamily M14C unassigned peptidases
MG-III	Cayman92	shallow_10126303	MER223020	M22	M22.UPW	8E-73	0.00	16.15	family M22 unassigned peptidases
MG-III	Cayman92	shallow_10063902	MER150270	M22	M22.UNW	3E-21	0.00	357.47	family M22 non-peptidase homologues
MG-III	Cayman92	shallow_10063901	MER071349	M24A	M24.035	1E-57	0.00	344.83	methionyl aminopeptidase (archaean-type)
MG-III	Cayman92	shallow_10043095	MER195385	M28A	M28.UPA	3E-58	0.00	50.62	subfamily M28A unassigned peptidases
MG-III	Cayman92	shallow_10015132	MER100706	M28A	M28.UPA	9E-19	0.00	39.81	subfamily M28A unassigned peptidases
MG-III	Cayman92	shallow_10113873	MER197705	M28C	M28.UPC	4E-16	0.00	0.00	subfamily M28C unassigned peptidases
MG-III	Cayman92	shallow_10047822	MER171696	M29	M29.UPW	1E-134	49.15	9347.30	family M29 unassigned peptidases
MG-III	Cayman92	shallow_10065502	MER159986	S08A	S08.UPA	1E-21	60.34	0.00	subfamily S8A unassigned peptidases
MG-III	Cayman93	Deep_100102616	MER195385	M28A	M28.UPA	3E-52	23.13	15.71	subfamily M28A unassigned peptidases
MG-III	Cayman93	Deep_10042583	MER159986	S08A	S08.UPA	2E-151	39.72	80.96	subfamily S8A unassigned peptidases
MG-III	Cayman93	Deep_10028227	MER145048	S08A	S08.UPA	4E-44	0.00	58.44	subfamily S8A unassigned peptidases
MG-III	Cayman93	Deep_10056652	MER161420	S15	S15.UPW	0	43.19	73.36	family S15 unassigned peptidases
MG-III	Cayman93	Deep_100102617	MER161420	S15	S15.UPW	0	0.00	22.11	family S15 unassigned peptidases
MG-III	Guaymas31	GBIDBA_1000129329	MER291592	C11	C11.UPW	1E-35	4.80	0.00	family C11 unassigned peptidases
MG-III	Guaymas31	GBIDBA_1000082857	MER234074	I87	I87.UPW	3E-58	19.20	1215.07	family I87 unassigned peptidase inhibitors
MG-III	Guaymas31	GBIDBA_100137704	MER284620	M06	M06.UPW	1E-53	46.02	43.68	family M6 unassigned peptidases
MG-III	Guaymas31	GBIDBA_10000219108	MER287932	M06	M06.UPW	4E-13	42.27	100.30	family M6 unassigned peptidases
MG-III	Guaymas31	GBIDBA_1000430611	MER284620	M06	M06.UPW	7E-50	19.48	123.29	family M6 unassigned peptidases
MG-III	Guaymas31	GBIDBA_1000075784	MER382071	M14A	M14.UPA	3E-15	39.49	93.71	subfamily M14A unassigned peptidases
MG-III	Guaymas31	GBIDBA_1000052548	MER308482	M14C	M14.UPC	3E-16	12.08	57.31	subfamily M14C unassigned peptidases
MG-III	Guaymas31	GBIDBA_100047375	MER223020	M22	M22.UPW	7E-76	16.96	40.24	family M22 unassigned peptidases
MG-III	Guaymas31	GBIDBA_1000105853	MER128658	M24B	M24.UPB	5E-36	32.01	113.94	subfamily M24B unassigned peptidases
MG-III	Guaymas31	GBIDBA_1000425610	MER195385	M28A	M28.UPA	4E-52	5.00	94.91	subfamily M28A unassigned peptidases
MG-III	Guaymas31	GBIDBA_10000339142	MER195385	M28A	M28.UPA	2E-59	31.27	63.60	subfamily M28A unassigned peptidases
MG-III	Guaymas31	GBIDBA_1000082820	MER100706	M28A	M28.UPA	6E-20	26.12	0.00	subfamily M28A unassigned peptidases
MG-III	Guaymas31	GBIDBA_100013604	MER348722	M48B	M48.UNB	9E-65	72.39	0.00	subfamily M48B non-peptidase homologues
MG-III	Guaymas31	GBIDBA_1000358714	MER054569	S08A	S08.UPA	7E-128	26.62	72.19	subfamily S8A unassigned peptidases
MG-III	Guaymas31	GBIDBA_100042564	MER182372	S08A	S08.UPA	6E-46	21.44	45.23	subfamily S8A unassigned peptidases
MG-III	Guaymas31	GBIDBA_1000129327	MER159986	S08A	S08.UPA	1E-20	6.93	32.91	subfamily S8A unassigned peptidases
MG-III	Guaymas31	GBIDBA_100056174	MER145048	S08A	S08.UPA	5E-43	36.45	57.66	subfamily S8A unassigned peptidases
MG-III	Guaymas31	GBIDBA_100003394	MER145048	S08A	S08.UPA	4E-42	12.57	0.00	subfamily S8A unassigned peptidases
MG-III	Guaymas31	GBIDBA_10000339143	MER161420	S15	S15.UPW	0	24.97	158.01	family S15 unassigned peptidases
MG-III	Guaymas32	GBIDBA_100119794	MER203535	A22B	A22.015	4E-57	0.00	78.92	MCMJR1 peptidase

MG-III	Guaymas32	GBIDBA_1000039144	MER087000	C11	C11.UPW	2E-31	11.87	0.00	family C11 unassigned peptidases
MG-III	Guaymas32	GBIDBA_100060701	MER229584	M06	M06.UPW	2E-11	3.54	50.39	family M6 unassigned peptidases
MG-III	Guaymas32	GBIDBA_100002021	MER162003	M06	M06.UPW	1E-47	16.44	0.00	family M6 unassigned peptidases
MG-III	Guaymas32	GBIDBA_100045817	MER284620	M06	M06.UPW	6E-40	16.34	0.00	family M6 unassigned peptidases
MG-III	Guaymas32	GBIDBA_100036708	MER242588	M14A	M14.UPA	6E-15	0.00	46.30	subfamily M14A unassigned peptidases
MG-III	Guaymas32	GBIDBA_100004696	MER088220	M14A	M14.UPA	1E-12	26.11	15.49	subfamily M14A unassigned peptidases
MG-III	Guaymas32	GBIDBA_1000324522	MER198363	M22	M22.UPW	2E-97	23.42	0.00	family M22 unassigned peptidases
MG-III	Guaymas32	GBIDBA_1000095728	MER198363	M22	M22.UPW	1E-98	0.00	0.00	family M22 unassigned peptidases
MG-III	Guaymas32	GBIDBA_100061574	MER195385	M28A	M28.UPA	3E-48	19.58	0.00	subfamily M28A unassigned peptidases
MG-III	Guaymas32	GBIDBA_100003936	MER195385	M28A	M28.UPA	7E-51	9.79	0.00	subfamily M28A unassigned peptidases
MG-III	Guaymas32	GBIDBA_100008698	MER195385	M28A	M28.UPA	7E-58	17.47	0.00	subfamily M28A unassigned peptidases
MG-III	Guaymas32	GBIDBA_1000429115	MER195385	M28A	M28.UPA	8E-31	0.00	0.00	subfamily M28A unassigned peptidases
MG-III	Guaymas32	GBIDBA_1000297412	MER296334	M48B	M48.UNB	2E-63	18.42	0.00	subfamily M48B non-peptidase homologues
MG-III	Guaymas32	GBIDBA_1000039310	MER317741	M82	M82.001	1E-19	0.00	0.00	PrsW peptidase
MG-III	Guaymas32	GBIDBA_1000127614	MER054569	S08A	S08.UPA	2E-137	7.44	70.62	subfamily S8A unassigned peptidases
MG-III	Guaymas32	GBIDBA_100014451	MER179414	S08A	S08.UPA	4E-28	5.18	49.17	subfamily S8A unassigned peptidases
MG-III	Guaymas32	GBIDBA_1000195322	MER054569	S08A	S08.UPA	5E-133	11.29	35.72	subfamily S8A unassigned peptidases
MG-III	Guaymas32	GBIDBA_1000039143	MER019280	S08A	S08.135	3E-23	16.05	0.00	tengconlysin
MG-III	Guaymas32	GBIDBA_100064563	MER137810	S08A	S08.UPA	9E-52	8.39	39.83	subfamily S8A unassigned peptidases
MG-III	Guaymas32	GBIDBA_1000077527	MER019280	S08A	S08.135	1E-35	0.00	0.00	tengconlysin
MG-III	Guaymas32	GBIDBA_100104327	MER019280	S08A	S08.135	8E-53	0.00	0.00	tengconlysin
MG-III	Guaymas32	GBIDBA_100019656	MER161420	S15	S15.UPW	1E-120	17.56	0.00	family S15 unassigned peptidases
MG-III	Guaymas32	GBIDBA_100054587	MER161420	S15	S15.UPW	1E-114	8.65	0.00	family S15 unassigned peptidases
Parvarchaeota	Guaymas33	GBIDBA_1000153217	MER240051	S01C	S01.UPC	4E-30	0.00	0.00	subfamily S1C unassigned peptidases
Parvarchaeota	Lau125	Abe_100084857	MER240051	S01C	S01.UPC	5E-30	-	-	subfamily S1C unassigned peptidases
Putative DHVEG-6	Guaymas35	GBIDBA_100037317	MER296343	M48B	M48.UNB	7E-66	0.00	0.00	subfamily M48B non-peptidase homologues
Putative DHVEG-6	Guaymas35	GBIDBA_1000784710	MER348719	M48B	M48.UNB	8E-66	0.00	0.00	subfamily M48B non-peptidase homologues

* indicates the relative abundance of transcripts (number of transcripts mapped was normalized to the length of the gene and total number of transcript reads from plume and background samples in each site)

Supplementary Table 6. Genes involved to carbohydrate active enzymes aligned to the CAZy database. “Aux. Act.”, auxiliary activities. PS, polysaccharide.

Group	Bin	Aux. Act.		Carbohydrate-binding module					Carbohydrate Esterase			Glycoside Hydrolase										Glycosyl Transferase												PS Lyase
		AA1	AAnc	CBM13	CBM34, GH13	CBM5	CBM5, GH18	CBM5, GH20	CE14	CE4	CEnc	GH13	GH3	GH36	GH38	GH39	GH57	GH73	GH77	GH81	GHnc	GT1	GT2	GT2, GT1	GT2, GT2	GT2, GT4	GT35	GT4	GT66	GT75	GT81	GT9	GTnc	PLnc
MG-I	Cayman91	0	0	0	0	0	0	0	0	0	1	0	0	0	0	0	0	0	0	0	0	0	3	0	0	0	0	4	0	0	0	0	0	0
MG-I	Cayman117	4	0	0	0	0	0	0	0	0	1	0	0	0	0	0	0	0	0	0	0	0	13	0	0	0	0	6	4	0	0	0	0	0
MG-I	Cayman118	1	0	0	0	0	0	0	0	0	0	0	0	0	0	0	0	0	0	0	0	3	1	0	0	0	0	1	2	0	0	0	1	0
MG-I	Guaymas69	5	0	0	0	0	0	0	13	1	20	0	0	0	0	0	0	0	0	0	0	4	81	0	2	4	0	72	5	0	0	0	31	0
MG-I	Guaymas96	4	0	0	0	0	0	0	0	0	0	0	0	0	0	0	0	0	0	0	0	3	7	0	0	0	0	0	3	0	0	0	0	0
MG-I	Lau19	23	0	0	0	0	0	0	54	0	36	0	0	0	0	0	0	0	0	0	0	13	306	0	0	2	0	154	48	1	1	0	42	0
MG-II	Cayman51	0	0	1	0	2	2	0	0	0	0	0	0	0	0	0	0	0	0	0	0	0	3	0	0	0	0	3	3	0	0	0	0	0
MG-II	Cayman59	0	0	2	0	3	3	1	0	0	0	0	1	0	0	0	0	0	0	0	0	0	4	0	0	0	0	7	3	0	0	0	0	0
MG-II	Cayman68	1	1	0	0	0	1	0	0	0	0	0	0	0	0	0	1	0	0	0	0	0	8	0	0	0	0	3	5	0	0	0	0	0
MG-II	Cayman69	1	0	0	0	0	0	0	0	0	0	0	0	1	1	0	1	1	1	0	0	0	2	0	0	0	1	2	0	0	0	0	0	1
MG-II	Cayman80	3	0	1	0	1	0	1	0	0	0	0	0	0	0	0	0	0	0	0	0	0	2	0	0	0	0	3	3	0	0	0	0	0
MG-II	Guaymas21	3	0	0	0	1	0	1	0	0	0	0	0	0	0	0	0	0	0	0	0	0	2	0	0	0	0	3	2	0	0	0	0	0
MG-II	Guaymas22	0	0	1	0	2	0	1	0	0	0	0	0	0	0	0	0	0	0	0	0	0	2	0	0	0	0	3	2	0	0	0	0	0
MG-II	Guaymas2	1	0	1	0	2	0	1	0	0	0	0	0	0	0	0	0	0	0	0	0	0	7	0	0	0	0	7	2	0	0	0	0	0

	3																																	
MG-II	Guaymas24	0	0	2	2	1	1	1	0	0	0	0	0	0	1	0	2	0	2	0	0	0	6	0	0	0	4	7	1	0	0	0	0	0
MG-II	Guaymas25	1	0	2	0	1	0	1	0	0	1	0	0	0	1	1	0	0	0	0	0	0	12	0	0	0	0	9	6	0	0	0	0	1
MG-II	Guaymas26	1	0	3	0	0	0	0	0	0	0	0	0	0	1	0	0	0	0	0	0	0	7	0	0	0	0	2	5	0	0	0	0	0
MG-II	Guaymas27	0	0	2	0	0	0	0	0	0	0	0	0	0	0	0	0	0	0	0	0	0	4	0	0	0	0	2	4	0	0	0	0	0
MG-II	Guaymas28	0	0	1	0	1	0	1	0	0	0	1	0	0	0	1	2	0	1	0	0	0	9	0	0	0	1	6	5	0	0	0	0	1
MG-II	Guaymas29	1	0	1	0	0	0	1	0	0	0	0	0	0	0	0	0	0	0	0	0	0	2	0	0	0	0	3	2	0	0	0	0	0
MG-II	Guaymas34	0	0	1	0	0	0	1	0	0	0	0	0	0	0	0	0	0	0	0	0	0	1	0	0	0	0	0	1	0	0	0	0	0
MG-II	Lau6	5	0	0	0	0	0	0	13	1	20	0	0	0	0	0	0	0	0	0	0	4	81	0	2	4	0	72	5	0	0	0	31	0
MG-II	Lau34	0	0	4	3	1	2	0	0	0	0	0	0	0	1	0	0	0	0	0	0	0	16	0	0	0	3	1	5	0	0	0	0	0
MG-II	Lau92	0	0	1	0	1	1	1	0	0	0	0	0	0	0	0	0	0	0	0	0	0	3	0	0	0	0	3	4	0	0	0	0	0
MG-II	Lau93	0	0	0	0	0	0	1	0	0	0	0	0	0	0	0	0	0	0	0	0	0	1	0	0	0	0	1	1	0	0	0	0	0
MG-III	Cayman92	0	0	0	0	1	1	3	1	0	0	0	0	0	0	0	0	0	0	0	0	0	0	0	0	0	0	5	2	0	0	0	0	0
MG-III	Cayman93	2	0	1	0	0	0	2	0	0	0	0	0	0	0	0	0	0	0	0	0	0	3	0	0	0	0	1	1	0	0	0	0	0
MG-III	Guaymas31	0	0	0	0	5	2	2	1	0	0	0	0	0	0	0	0	0	0	0	1	0	4	0	0	0	0	7	4	0	0	0	0	0
MG-III	Guaymas32	5	0	3	0	3	4	4	2	0	0	0	0	0	0	0	0	0	0	1	0	0	7	0	0	0	0	16	5	0	0	0	0	0
Parvarchaeota	Guaymas33	0	0	0	0	0	0	0	0	0	0	0	0	0	0	0	0	0	0	0	0	0	1	0	0	0	0	4	1	0	0	0	0	0
Parvarchaeota	Lau125	0	0	0	0	0	0	0	0	0	0	0	0	0	0	0	0	0	0	0	0	0	2	0	0	0	0	0	1	0	0	0	0	0
DHVEG6	Guaymas35	0	0	0	0	0	0	0	2	0	0	0	0	0	0	0	0	0	0	0	0	0	10	2	0	0	0	5	2	0	0	1	2	0

Supplementary Table 7. GH family in deep-sea MG-II and MG-III

Group	Bin	Locus	Best-hit	Family	E-value	Background*	Plume*	Annotation
α-amylase								
MG-II	Guaymas28	GBIDBA_1000577913	AEH25509.1	GH13	5.00E-34	6.43	64.78	Alpha amylase, catalytic domain subfamily
β-glucosidase								
MG-II	Cayman59	shallow_10075591	ABW02044.1	GH3	3.00E-12	0.00	0.00	glycoside hydrolase family 3 domain protein
α-galactosidase								
MG-II	Cayman69	Deep_10009349	ACM58429.1	GH36	1.00E-16	0.00	26.21	Alpha-galactosidase-like protein
α-mannosidase								
MG-II	lau6	Mariner_100001638	ADT82990.1	GH38	6.00E-90			putative alpha-mannosidase
MG-II	lau6	KiloMoana_1000009735	ADT82990.1	GH38	7.00E-90			putative alpha-mannosidase
MG-II	lau6	TahiMoana_100019439	ADT82990.1	GH38	7.00E-90			putative alpha-mannosidase
MG-II	Guaymas24	GBIDBA_1000098222	ADT82990.1	GH38	6.00E-88	0.00	68.28	putative alpha-mannosidase
MG-II	Guaymas26	GBIDBA_1000013918	ADT82990.1	GH38	8.00E-78	9.96	87.73	putative alpha-mannosidase
MG-II	Guaymas25	GBIDBA_100011039	ADT82990.1	GH38	1.00E-75	5.11	90.06	putative alpha-mannosidase
MG-II	Lau34	Mariner_10068042	ADT82990.1	GH38	6.00E-33			putative alpha-mannosidase
MG-II	Cayman69	Deep_10058121	ADT82990.1	GH38	4.00E-30	0.00	16.22	putative alpha-mannosidase
β-xylosidase								
MG-II	lau6	KiloMoana_100003612	ABD40448.1	GH39	2.00E-13			glycoside hydrolase, family 39
MG-II	Guaymas25	GBIDBA_100115604	ABD40448.1	GH39	5.00E-12	13.07	109.64	glycoside hydrolase, family 39
MG-II	Guaymas28	GBIDBA_100105725	ABD40448.1	GH39	9.00E-11	3.88	0.00	glycoside hydrolase, family 39
Pullulanase								
MG-II	Cayman68	shallow_100431314	AEH25371.1	GH57	1.00E-14	0.00	11.78	pullulanase
Amylopullulanase								
MG-II	Guaymas24	GBIDBA_100096046	CAC11276.1	GH57	5.00E-82	7.05	17.74	amylopullulanase related protein
MG-II	lau6	KiloMoana_1000011434	CAC11276.1	GH57	1.00E-80			amylopullulanase related protein
MG-II	lau6	TahiMoana_100004337	CAC11276.1	GH57	1.00E-80			amylopullulanase related protein
MG-II	lau6	Mariner_100004827	CAC11276.1	GH57	3.00E-80			amylopullulanase related protein
MG-II	Guaymas24	GBIDBA_1000019925	CAC11276.1	GH57	3.00E-61	0.00	89.65	amylopullulanase related protein
MG-II	Cayman69	Deep_10052243	CAC11276.1	GH57	2.00E-60	0.00	9.76	amylopullulanase related protein
MG-II	Guaymas28	GBIDBA_1000577917	CAC11276.1	GH57	6.00E-42	7.51	37.78	amylopullulanase related protein
MG-II	Guaymas28	GBIDBA_1000143427	CAC11276.1	GH57	2.00E-33	0.00	0.00	amylopullulanase related protein
peptidoglycan hydrolase								
MG-II	Cayman69	Deep_10026922	AEB68921.1	GH73	6.00E-11	47.44	5.37	hypothetical protein MCON_2475
4-α-glucanotransferase								
MG-II	Cayman69	Deep_10052242	ADL19304.1	GH77	3.00E-93	0.00	0.00	4-alpha-glucanotransferase
MG-II	lau6	Mariner_100004828	AEA11843.1	GH77	1.00E-91			4-alpha-glucanotransferase
MG-II	lau6	TahiMoana_100004338	AEA11843.1	GH77	1.00E-91			4-alpha-glucanotransferase
MG-II	lau6	KiloMoana_1000011435	AEA11843.1	GH77	3.00E-91			4-alpha-glucanotransferase
MG-II	Guaymas24	GBIDBA_100096045	AEA11843.1	GH77	3.00E-90	0.00	0.00	4-alpha-glucanotransferase
MG-II	Guaymas28	GBIDBA_1000577916	AEA11843.1	GH77	2.00E-88	0.00	233.10	4-alpha-glucanotransferase
MG-II	Guaymas24	GBIDBA_1000019926	AEA11843.1	GH77	5.00E-85	12.77	64.29	4-alpha-glucanotransferase
Chitinases								
MG-II	Cayman68	shallow_10105817	ACV49026.1	CBM5,GH18	2.00E-17	0.00	0.00	PKD domain containing protein

MG-II	Cayman51	Deep_100010369	AGN00773.1	CBM5,GH18	3.00E-13	0.00	32.93	glycoside hydrolase family protein
MG-II	Cayman51	Deep_100001982	AGN00773.1	CBM5,GH18	3.00E-13	0.00	0.00	glycoside hydrolase family protein
MG-II	Cayman59	shallow_100258910	AGN00773.1	CBM5,GH18	3.00E-13	0.00	31.02	glycoside hydrolase family protein
MG-II	Cayman59	shallow_10004672	AGN00773.1	CBM5,GH18	3.00E-13	0.00	11.75	glycoside hydrolase family protein
MG-II	Lau92	Mariner_10051435	AGN00773.1	CBM5,GH18	2.00E-12			glycoside hydrolase family protein
MG-II	Cayman59	shallow_10016696	AGN00773.1	CBM5,GH18	3.00E-12	0.00	5.54	glycoside hydrolase family protein
MG-II	Guaymas24	GBIDBA_1000088811	AGN02077.1	CBM5,GH18	9.00E-12	12.71	0.00	chitinase
MG-II	Lau34	KiloMoana_1000005157	AGN02077.1	CBM5,GH18	3.00E-11			chitinase
MG-II	Lau34	TahiMoana_10001231	AGN02077.1	CBM5,GH18	3.00E-11			chitinase
MG-II	lau6	Mariner_100012323	AGN02077.1	CBM5,GH18	4.00E-11			chitinase
Chitinase/Collegenase								
MG-II	Guaymas25	GBIDBA_100256954	ACV47620.1	CBM5,GH20	9.00E-20	30.48	30.68	Glycoside hydrolase, family 20, catalytic core
MG-II	Guaymas29	GBIDBA_1000087754	ACV47620.1	CBM5,GH20	1.00E-17	36.30	121.81	Glycoside hydrolase, family 20, catalytic core
MG-II	Lau93	KiloMoana_100156348	ACV47620.1	CBM5,GH20	1.00E-17			Glycoside hydrolase, family 20, catalytic core
MG-II	Guaymas34	GBIDBA_100320099	ACV47620.1	CBM5,GH20	7.00E-17	19.26	0.00	Glycoside hydrolase, family 20, catalytic core
MG-II	Guaymas24	GBIDBA_1000027216	ACV47620.1	CBM5,GH20	1.00E-16	24.44	0.00	Glycoside hydrolase, family 20, catalytic core
MG-II	Guaymas22	GBIDBA_1000077024	ACV47620.1	CBM5,GH20	8.00E-16	0.00	0.00	Glycoside hydrolase, family 20, catalytic core
MG-II	Cayman59	shallow_100375410	ACV47620.1	CBM5,GH20	1.00E-15	4.41	0.00	Glycoside hydrolase, family 20, catalytic core
MG-II	lau6	KiloMoana_1000036120	ACV47620.1	CBM5,GH20	1.00E-15			Glycoside hydrolase, family 20, catalytic core
MG-II	lau6	Mariner_10007252	ACV47620.1	CBM5,GH20	1.00E-15			Glycoside hydrolase, family 20, catalytic core
MG-II	lau6	TahiMoana_100032318	ACV47620.1	CBM5,GH20	1.00E-15			Glycoside hydrolase, family 20, catalytic core
MG-II	Lau92	Mariner_100195110	ACV47620.1	CBM5,GH20	1.00E-15			Glycoside hydrolase, family 20, catalytic core
MG-II	Guaymas28	GBIDBA_100089066	ACV47620.1	CBM5,GH20	7.00E-15	0.00	0.00	Glycoside hydrolase, family 20, catalytic core
MG-II	Cayman80	Deep_10009459	ACV47620.1	CBM5,GH20	1.00E-13	0.00	34.78	Glycoside hydrolase, family 20, catalytic core
MG-II	Guaymas21	GBIDBA_100036817	ACV47620.1	CBM5,GH20	1.00E-13	141.99	186.46	Glycoside hydrolase, family 20, catalytic core
MG-II	Guaymas23	GBIDBA_1000024028	ACV47620.1	CBM5,GH20	8.00E-13	0.00	373.87	Glycoside hydrolase, family 20, catalytic core
endo-β-1,3-glucanase								
MG-III	Guaymas32	GBIDBA_100112102	AEH38967.1	GH81	4.00E-118	0.00	0.00	glycoside hydrolase family 81
Chitinase								
MG-III	Guaymas31	GBIDBA_1000430611	ACV49026.1	CBM5,GH18	2.00E-25	18.37	123.29	PKD domain containing protein
MG-III	Cayman92	shallow_10114731	ACV49026.1	CBM5,GH18	1.00E-24	0.00	9.32	PKD domain containing protein
MG-III	Guaymas32	GBIDBA_1000195324	ACV49026.1	CBM5,GH18	1.00E-18	3.08	46.48	PKD domain containing protein
MG-III	Guaymas32	GBIDBA_1000125015	ACV49026.1	CBM5,GH18	4.00E-17	0.00	67.89	PKD domain containing protein
MG-III	Guaymas32	GBIDBA_1000053541	ACV49026.1	CBM5,GH18	2.00E-12	27.36	0.00	PKD domain containing protein
MG-III	Guaymas32	GBIDBA_10000469109	AGN00773.1	CBM5,GH18	6.00E-21	0.00	0.00	glycoside hydrolase family protein
MG-III	Guaymas31	GBIDBA_1000052562	AGN00773.1	CBM5,GH18	1.00E-17	52.20	0.00	glycoside hydrolase family protein
Chitinase								
MG-III	Cayman93	Deep_10065163	ACV47620.1	CBM5,GH20	1.00E-25	56.19	57.27	Glycoside hydrolase, family 20, catalytic core
MG-III	Guaymas32	GBIDBA_1000053545	ACV47620.1	CBM5,GH20	2.00E-21	0.00	0.00	Glycoside hydrolase, family 20, catalytic core
MG-III	Guaymas32	GBIDBA_1000127616	ACV47620.1	CBM5,GH20	3.00E-20	6.07	76.39	Glycoside hydrolase, family 20, catalytic core
MG-III	Cayman92	shallow_10018753	ACV47620.1	CBM5,GH20	2.00E-17	0.00	39.54	Glycoside hydrolase, family 20, catalytic core
MG-III	Cayman92	shallow_10108997	ACV47620.1	CBM5,GH20	3.00E-17	0.00	72.25	Glycoside hydrolase, family 20, catalytic core
MG-III	Guaymas32	GBIDBA_1000007750	ACV47620.1	CBM5,GH20	5.00E-17	19.15	24.10	Glycoside hydrolase, family 20, catalytic core

MG-III	Cayman93	Deep_10066901	ACV47620.1	CBM5,GH20	6.00E-17	0.00	74.08	Glycoside hydrolase, family 20, catalytic core
MG-III	Guaymas31	GBIDBA_1000021962	ACV47620.1	CBM5,GH20	4.00E-16	109.73	0.00	Glycoside hydrolase, family 20, catalytic core
MG-III	Cayman92	shallow_10080315	ACV47620.1	CBM5,GH20	8.00E-16	0.00	13.85	Glycoside hydrolase, family 20, catalytic core
MG-III	Guaymas32	GBIDBA_10000469106	ACV47620.1	CBM5,GH20	5.00E-11	6.99	46.89	Glycoside hydrolase, family 20, catalytic core
MG-III	Guaymas31	GBIDBA_1000021963	ACV47620.1	CBM5,GH20	7.00E-11	35.92	16.44	Glycoside hydrolase, family 20, catalytic core

* indicate the relative abundance of transcripts (number of transcripts mapped was normalized to the length of the gene and total number of transcript reads from plume and background samples in each site).

Supplementary Table 8. Genes involved in lipid metabolism in uncultured deep-sea archaea.

Group	Bin	Locus	Plume*	Background*	EC. No	Nr_top	Nr taxonomy
MG-I	Cayman117	shallow_100132914	0.00	6.01	EC:1.1.1.157	putative 3-hydroxyacyl-CoA dehydrogenase, NAD binding domain protein	Candidatus Nitrosopumilus sp. AR2
MG-I	Cayman117	shallow_10096891	0.00	0.00	EC:1.1.1.157	putative 3-hydroxyacyl-CoA dehydrogenase, NAD binding domain protein	Candidatus Nitrosopumilus koreensis AR1
MG-I	Cayman117	shallow_10013179	0.00	0.00	EC:2.3.1.9	putative thiolase, C-terminal domain protein	Nitrosopumilus maritimus SCM1
MG-I	Cayman117	shallow_10036332	25.20	0.00	EC:2.3.1.9	putative thiolase, C-terminal domain protein	Candidatus Nitrosopumilus sp. AR2
MG-I	Cayman117	shallow_10097204	0.00	0.00	EC:2.3.1.9	putative thiolase, C-terminal domain protein	Marine archaeal group 1 BG20
MG-I	Cayman117	shallow_10116646	0.00	0.00	EC:2.3.1.9	putative thiolase, C-terminal domain protein	Candidatus Nitrosoarchaeum limnia SFB1
MG-I	Cayman117	shallow_10137435	13.22	0.00	EC:2.3.1.9	putative thiolase, C-terminal domain protein	Candidatus Nitrosoarchaeum limnia SFB1
MG-I	Cayman117	shallow_10065976	0.00	0.00		putative carboxyl transferase domain protein	Candidatus Nitrosopumilus salaria BD31
MG-I	Cayman117	shallow_10067586	0.00	0.00	EC:6.2.1.3	AMP-dependent synthetase and ligase	Caldalkalibacillus thermarum TA2.A1
MG-I	Cayman117	shallow_10084076	7.61	0.00	EC:6.2.1.1	putative AMP-binding enzyme	Candidatus Nitrosopumilus koreensis AR1
MG-I	Cayman117	shallow_10144995	0.00	0.00	EC:6.2.1.1	putative AMP-binding enzyme	Candidatus Nitrosopumilus koreensis AR1
MG-I	Cayman117	shallow_10021172	115.20	0.00	EC:4.2.1.55	putative enoyl-CoA hydratase/isomerase family protein	Marine archaeal group 1 BG20
MG-I	Cayman117	shallow_10141555	0.00	0.00	EC:4.2.1.55	putative enoyl-CoA hydratase/isomerase family protein	Marine archaeal group 1 BG20
MG-I	Cayman117	shallow_10076733	0.00	0.00		hypothetical protein	Candidatus Nitrosopumilus salaria BD31
MG-I	Cayman118	shallow_10028544	0.00	0.00	EC:1.1.1.157	3-hydroxyacyl-CoA dehydrogenase	Ferroplasma acidarmanus Fer1
MG-I	Cayman118	shallow_10028546	0.00	0.00	EC:2.3.1.9	acetoacetyl-CoA thiolase	Candidatus Nitrosopumilus sp. AR2
MG-I	Cayman118	shallow_10028543	0.00	0.00	EC:4.2.1.55	enoyl-CoA hydratase/carnithine racemase	Thermoanaerobacter indiensis BSB-33
MG-I	Cayman118	shallow_100098819	0.00	0.00	EC:6.2.1.1	putative AMP-binding enzyme	Candidatus Nitrosopumilus koreensis AR1
MG-I	Cayman118	shallow_100231615	0.00	0.00		putative carboxyl transferase domain protein	Candidatus Nitrosopumilus sp. AR2
MG-I	Guaymas69	GBIDBA_100065386	0.00	0.00		3-oxoacyl-ACP synthase	Acidobacteriaceae bacterium KBS 83
MG-I	Guaymas69	GBIDBA_100093574	0.00	0.00		3-oxoacyl-ACP synthase	Pedobacter oryzae DSM 19973
MG-I	Guaymas69	GBIDBA_100061274	0.00	0.00	EC:6.2.1.3	AMP-dependent synthetase and ligase	Nitrosopumilus maritimus SCM1
MG-I	Guaymas69	GBIDBA_100119387	0.00	0.00	EC:6.2.1.3	AMP-dependent synthetase and ligase	Nitrosopumilus maritimus SCM1
MG-I	Guaymas69	GBIDBA_100048511	0.00	0.00	EC:2.1.3.-	carbamoyl transferase	Nitritalea halalkaliphila LW7
MG-I	Guaymas69	GBIDBA_100084831	0.00	161.21	EC:4.2.1.55	enoyl-CoA hydratase/isomerase	Nitrosopumilus maritimus SCM1
MG-I	Guaymas69	GBIDBA_100330502	34.13	0.00	EC:2.3.1.9	propanoyl-CoA C-acyltransferase	Nitrosopumilus maritimus SCM1
MG-I	Guaymas69	GBIDBA_100146742	0.00	41.63	EC:2.3.1.9	putative thiolase, C-terminal domain protein	Candidatus Nitrosoarchaeum limnia SFB1
MG-I	Guaymas96	GBIDBA_1001341914	0.00	26.95	EC:1.1.1.157	putative 3-hydroxyacyl-CoA dehydrogenase, NAD binding domain protein	Marine archaeal group 1 BG20
MG-I	Guaymas96	GBIDBA_1001142614	0.00	19.84		putative carboxyl transferase domain protein	Candidatus Nitrosopumilus salaria BD31
MG-I	Guaymas96	GBIDBA_100098125	0.00	24.80		putative carboxyl transferase domain protein	Marine archaeal group 1 BG20
MG-I	Guaymas96	GBIDBA_1001552111	95.72	90.75	EC:4.2.1.55	putative enoyl-CoA hydratase/isomerase family protein	Marine archaeal group 1 BG20
MG-I	Guaymas96	GBIDBA_100110228	47.86	40.33	EC:4.2.1.55	putative enoyl-CoA hydratase/isomerase family protein	Marine archaeal group 1 BG20
MG-I	Guaymas96	GBIDBA_100093884	0.00	39.69	EC:2.3.1.9	putative thiolase, C-terminal domain protein	Candidatus Nitrosopumilus sp. AR2
MG-II	Cayman51	Deep_100001960	47.41	17.45	EC:1.1.1.35	3-hydroxyacyl-CoA dehydrogenase	Thiothrix flexilis DSM 14609
MG-II	Cayman51	Deep_100035239	27.52	0.00	EC:1.1.1.35	bifunctional 3-hydroxyacyl-CoA	uncultured marine group II euryarchaeote

						dehydrogenase/enoyl-CoA hydratase/isomerase family protein	
MG-II	Cayman51	Deep_100010311	23.43	0.00	EC:2.3.1.9	acetyl-CoA C-acetyltransferase	uncultured marine group II euryarchaeote
MG-II	Cayman51	Deep_100019513	77.03	0.00	EC:2.3.1.9	3-ketoacyl-CoA thiolase	uncultured marine group II euryarchaeote
MG-II	Cayman51	Deep_1000059171	26.26	0.00	EC:6.4.1.3	carboxyl transferase	uncultured marine group II euryarchaeote
MG-II	Cayman51	Deep_100006794	8.77	0.00	EC:6.4.1.3	putative carboxyl transferase	uncultured marine group II euryarchaeote
MG-II	Cayman51	Deep_100001959	72.54	0.00	EC:2.3.1.16	acetyl-CoA acetyltransferase	Methylocaldum szegediense O-12
MG-II	Cayman51	Deep_1000019192	17.44	0.00	EC:6.4.1.4	carboxyltransferase subunit of acetyl-CoA carboxylase	Streptosporangium roseum NI 9100, DSM 43021
MG-II	Cayman51	Deep_1000019193	0.00	0.00	EC:4.2.1.18	enoyl-CoA hydratase / methylglutaconyl-CoA hydratase	uncultured marine group II euryarchaeote
MG-II	Cayman51	Deep_10000554	31.93	0.00	EC:1.1.1.157	3-hydroxybutyryl-CoA dehydrogenase / 3-hydroxyacyl-CoA dehydrogenase	uncultured marine group II euryarchaeote
MG-II	Cayman51	Deep_100005941	0.00	70.61	EC:2.3.1.16	putative thiolase, N-terminal domain protein	Burkholderia graminis C4D1M
MG-II	Cayman51	Deep_100006729	113.75	0.00	EC:4.2.1.55	enoyl CoA hydratase/isomerase	uncultured marine group II euryarchaeote
MG-II	Cayman51	Deep_100006738	113.75	0.00	EC:1.1.1.157	3-hydroxybutyryl-CoA dehydrogenase / 3-hydroxyacyl-CoA dehydrogenase	uncultured marine group II euryarchaeote
MG-II	Cayman51	Deep_100006741	323.83	0.00	EC:4.2.1.17	Enoyl-CoA hydratase / isomerase	uncultured marine group II euryarchaeote
MG-II	Cayman51	Deep_100006776	182.56	0.00	EC:6.2.1.3	putative fatty-acid--CoA ligase	SAR406 cluster bacterium JGI 0000113-D11
MG-II	Cayman59	shallow_100050922	0.00	0.00	EC:1.1.1.157	3-hydroxybutyryl-CoA dehydrogenase / 3-hydroxyacyl-CoA dehydrogenase	uncultured marine group II euryarchaeote
MG-II	Cayman59	shallow_10055433	0.00	0.00	EC:2.3.1.16	acetyl-CoA acetyltransferase	Methylocaldum szegediense O-12
MG-II	Cayman59	shallow_10053974	0.00	21.87	EC:2.3.1.9	acetyl-CoA C-acetyltransferase	uncultured marine group II euryarchaeote
MG-II	Cayman59	shallow_10016697	0.00	0.00	EC:1.1.1.35	bifunctional 3-hydroxyacyl-CoA dehydrogenase/enoyl-CoA hydratase/isomerase family protein	uncultured marine group II euryarchaeote
MG-II	Cayman59	shallow_10007897	0.00	0.00	EC:6.4.1.3	carboxyl transferase	uncultured marine group II euryarchaeote
MG-II	Cayman59	shallow_10044887	0.00	0.00	EC:6.4.1.4	carboxyltransferase subunit of acetyl-CoA carboxylase	Bacteriovorax marinus SJ
MG-II	Cayman59	shallow_100121222	0.00	0.00	EC:4.2.1.17	Enoyl-CoA hydratase / isomerase	uncultured marine group II euryarchaeote
MG-II	Cayman59	shallow_10006623	25.80	0.00	EC:2.3.1.16	putative thiolase, N-terminal domain protein	Burkholderia graminis C4D1M
MG-II	Cayman59	shallow_10038944	24.95	0.00	EC:2.3.1.16	putative thiolase, N-terminal domain protein	Martelella mediterranea DSM 17316
MG-II	Cayman59	shallow_100035945	9.38	0.00	EC:6.4.1.4	carboxyltransferase subunit of acetyl-CoA carboxylase	Streptosporangium roseum NI 9100, DSM 43021
MG-II	Cayman59	shallow_10107242	11.84	0.00	EC:2.3.1.9	3-ketoacyl-CoA thiolase	uncultured marine group II euryarchaeote
MG-II	Cayman59	shallow_10109824	11.84	0.00	EC:2.3.1.9	3-ketoacyl-CoA thiolase	uncultured marine group II euryarchaeote
MG-II	Cayman59	shallow_10011387	12.60	0.00	EC:2.3.1.9	acetyl-CoA C-acetyltransferase	uncultured marine group II euryarchaeote
MG-II	Cayman59	shallow_10055432	19.15	8.97	EC:1.1.1.35	3-hydroxyacyl-CoA dehydrogenase	Thiothrix flexilis DSM 14609
MG-II	Cayman59	shallow_10136665	8.14	0.00	EC:6.2.1.3	putative fatty-acid--CoA ligase	SAR406 cluster bacterium JGI 0000113-D11
MG-II	Cayman59	shallow_10044886	0.00	0.00	EC:4.2.1.18	enoyl-CoA hydratase / methylglutaconyl-CoA hydratase	uncultured marine group II euryarchaeote
MG-II	Cayman59	shallow_100031329	17.17	0.00	EC:1.1.1.157	3-hydroxybutyryl-CoA dehydrogenase / 3-hydroxyacyl-CoA dehydrogenase	uncultured marine group II euryarchaeote
MG-II	Cayman59	shallow_10058634	18.87	0.00	EC:6.4.1.3	putative carboxyl transferase	uncultured marine group II euryarchaeote
MG-II	Cayman59	shallow_100046724	19.12	0.00	EC:1.1.1.35	3-hydroxyacyl-CoA dehydrogenase	Thiothrix flexilis DSM 14609

MG-II	Cayman59	shallow_100121210	34.96	139.26	EC:4.2.1.55	enoyl CoA hydratase/isomerase	uncultured marine group II euryarchaeote
MG-II	Cayman59	shallow_10025899	28.40	0.00	EC:1.1.1.35	bifunctional 3-hydroxyacyl-CoA dehydrogenase/enoyl-CoA hydratase/isomerase family protein	uncultured marine group II euryarchaeote
MG-II	Cayman59	shallow_10128192	47.16	0.00	EC:6.4.1.3	putative carboxyl transferase	uncultured marine group II euryarchaeote
MG-II	Cayman59	shallow_100121219	50.10	0.00	EC:1.1.1.157	3-hydroxybutyryl-CoA dehydrogenase / 3-hydroxyacyl-CoA dehydrogenase	uncultured marine group II euryarchaeote
MG-II	Cayman59	shallow_100079022	52.21	0.00	EC:6.4.1.3	carboxyl transferase	uncultured marine group II euryarchaeote
MG-II	Cayman59	shallow_100050913	34.96	0.00	EC:4.2.1.55	enoyl CoA hydratase/isomerase	uncultured marine group II euryarchaeote
MG-II	Cayman59	shallow_100050925	49.76	0.00	EC:4.2.1.17	Enoyl-CoA hydratase / isomerase	uncultured marine group II euryarchaeote
MG-II	Cayman59	shallow_100035946	67.04	0.00	EC:4.2.1.18	enoyl-CoA hydratase / methylglutaconyl-CoA hydratase	uncultured marine group II euryarchaeote
MG-II	Cayman59	shallow_100046725	52.02	0.00	EC:2.3.1.16	acetyl-CoA acetyltransferase	Methylocaldum szegediense O-12
MG-II	Cayman59	shallow_100050961	98.19	0.00	EC:6.2.1.3	putative fatty-acid--CoA ligase	SAR406 cluster bacterium JGI 0000113-D11
MG-II	Cayman68	shallow_100331511	0.00	0.00	EC:6.4.1.3	carboxyl transferase	uncultured marine group II euryarchaeote
MG-II	Cayman68	shallow_10057684	0.00	0.00	EC:4.2.1.55	enoyl CoA hydratase/isomerase	uncultured marine group II euryarchaeote
MG-II	Cayman68	shallow_10067092	0.00	0.00	EC:6.4.1.3	putative carboxyl transferase	uncultured marine group II euryarchaeote
MG-II	Cayman68	shallow_10077074	0.00	0.00	EC:1.1.1.157	3-hydroxybutyryl-CoA dehydrogenase / 3-hydroxyacyl-CoA dehydrogenase	uncultured marine group II euryarchaeote
MG-II	Cayman68	shallow_10053337	12.83	0.00	EC:2.3.1.16	3-ketoacyl-CoA thiolase	Tistrella mobilis KA081020-065
MG-II	Cayman68	shallow_10117802	0.00	0.00	EC:2.3.1.16	3-ketoacyl-CoA thiolase	Methylocaldum szegediense O-12
MG-II	Cayman68	shallow_10122902	25.20	0.00	EC:2.3.1.9	acetyl-CoA C-acetyltransferase	uncultured marine group II euryarchaeote
MG-II	Cayman68	shallow_10117801	140.41	4.39	EC:1.1.1.35	Enoyl-CoA hydratase	Novispirillum itersonii itersonii ATCC 12639
MG-II	Cayman68	shallow_10141565	0.00	0.00	EC:1.3.8.6	glutaryl-CoA dehydrogenase	uncultured marine group II euryarchaeote
MG-II	Cayman68	shallow_10134262	42.53	6.64	EC:2.3.1.16	putative thiolase, N-terminal domain protein	Tistrella mobilis KA081020-065
MG-II	Cayman69	Deep_10049207	0.00	0.00	EC:2.3.1.9	3-ketoacyl-CoA thiolase	uncultured marine group II euryarchaeote
MG-II	Cayman69	Deep_10053184	11.71	0.00	EC:2.3.1.9	acetyl-CoA C-acetyltransferase	uncultured marine group II euryarchaeote
MG-II	Cayman69	Deep_10029851	37.47	0.00	EC:1.3.99.2	Acyl-CoA dehydrogenase	uncultured marine group II euryarchaeote
MG-II	Cayman69	Deep_10049256	0.00	0.00	EC:4.2.1.17	enoyl CoA hydratase/isomerase	uncultured marine group II euryarchaeote
MG-II	Cayman69	Deep_10064044	6.99	0.00	EC:6.2.1.3	long-chain acyl-CoA synthetase	uncultured marine group II euryarchaeote
MG-II	Cayman69	Deep_10141854	0.00	0.00	EC:3.3.1.1	S-adenosyl-L-homocysteine hydrolase	uncultured marine group II euryarchaeote
MG-II	Cayman80	Deep_100109322	0.00	25.18	EC:6.4.1.4	3-methylcrotonyl-CoA carboxylase, carboxyltransferase component	uncultured marine group II euryarchaeote
MG-II	Cayman80	shallow_10145294	11.71	0.00	EC:2.3.1.9	acetyl-CoA C-acetyltransferase	uncultured marine group II euryarchaeote
MG-II	Cayman80	Deep_10108143	0.00	0.00		Acyl-CoA dehydrogenase	uncultured marine group II euryarchaeote
MG-II	Cayman80	Deep_100257612	0.00	0.00	EC:4.2.1.17	crotonase/enoyl-coenzyme A (CoA) hydratase superfamily protein	uncultured marine group II euryarchaeote
MG-II	Cayman80	Deep_100109323	0.00	0.00	EC:4.2.1.18	enoyl-CoA hydratase / methylglutaconyl-CoA hydratase	uncultured marine group II euryarchaeote
MG-II	Cayman80	Deep_100465019	0.00	0.00	EC:6.4.1.3	putative carboxyl transferase	uncultured marine group II euryarchaeote
MG-II	Cayman80	Deep_100465020	0.00	0.00	EC:6.4.1.3	putative carboxyl transferase	uncultured marine group II euryarchaeote
MG-II	Cayman80	Deep_100033112	11.33	0.00	EC:1.1.1.35	3-hydroxyacyl-CoA dehydrogenase	Novispirillum itersonii itersonii ATCC 12639
MG-II	Cayman80	Deep_10010158	13.16	0.00	EC:1.1.1.35	bifunctional 3-hydroxyacyl-CoA dehydrogenase/enoyl-CoA hydratase/isomerase family protein	uncultured marine group II euryarchaeote

MG-II	Cayman80	Deep_10025769	15.26	0.00	EC:1.1.1.157	3-hydroxybutyryl-CoA dehydrogenase / 3-hydroxyacyl-CoA dehydrogenase	uncultured marine group II euryarchaeote
MG-II	Cayman80	Deep_10044523	16.25	0.00	EC:4.2.1.55	enoyl CoA hydratase/isomerase	uncultured marine group II euryarchaeote
MG-II	Cayman80	Deep_100476713	11.51	0.00	EC:2.3.1.16	putative thiolase, N-terminal domain protein	Novispirillum itersonii itersonii ATCC 12639
MG-II	Cayman80	Deep_10066314	25.61	0.00	EC:6.4.1.3	carboxyl transferase	uncultured marine group II euryarchaeote
MG-II	Cayman80	Deep_100076918	35.14	0.00	EC:2.3.1.9	acetyl-CoA C-acetyltransferase	uncultured marine group II euryarchaeote
MG-II	Cayman80	Deep_10066605	71.82	0.00	EC:6.2.1.3	AMP-dependent synthetase	SAR406 cluster bacterium JGI 0000113-D11
MG-II	Cayman80	Deep_10025765	31.70	0.00	EC:4.2.1.17	Enoyl-CoA hydratase / isomerase	uncultured marine group II euryarchaeote
MG-II	Cayman80	Deep_100033111	47.85	0.00	EC:2.3.1.16	3-ketoacyl-CoA thiolase	Methylocaldum szegediense O-12
MG-II	Cayman80	Deep_10016205	55.02	0.00	EC:2.3.1.9	3-ketoacyl-CoA thiolase	uncultured marine group II euryarchaeote
MG-II	Cayman91	Deep_10028239	0.00	0.00	EC:2.3.1.9	putative thiolase, C-terminal domain protein	Candidatus Nitrosoarchaeum limnia SFB1
MG-II	Cayman91	Deep_10100395	43.76	0.00	EC:6.2.1.1	putative AMP-binding enzyme	Candidatus Nitrosopumilus koreensis AR1
MG-II	Guaymas21	GBIDBA_1000804512	288.70	130.89	EC:6.2.1.3	AMP-dependent synthetase and ligase	Haliangium ochraceum DSM 14365
MG-II	Guaymas21	GBIDBA_1000094013	74.36	38.58	EC:4.2.1.17	crotonase/enoyl-coenzyme A (CoA) hydratase superfamily protein	uncultured marine group II euryarchaeote
MG-II	Guaymas21	GBIDBA_1000094021	226.40	219.51	EC:4.2.1.17	Enoyl-CoA hydratase / isomerase	uncultured marine group II euryarchaeote
MG-II	Guaymas21	GBIDBA_100078996	48.69	46.18	EC:1.1.1.100	3-ketoacyl-(acyl-carrier-protein) reductase	uncultured marine group II euryarchaeote
MG-II	Guaymas21	GBIDBA_1000032530	29.10	16.36	EC:6.4.1.4	3-methylcrotonyl-CoA carboxylase alpha subunit	uncultured marine group II euryarchaeote
MG-II	Guaymas21	GBIDBA_1000032527	38.88	30.26	EC:6.4.1.4	3-methylcrotonyl-CoA carboxylase, carboxyltransferase component	uncultured marine group II euryarchaeote
MG-II	Guaymas21	GBIDBA_100015632	76.83	259.12	EC:1.1.1.35	3-hydroxyacyl-CoA dehydrogenase	Tistrella mobilis KA081020-065
MG-II	Guaymas21	GBIDBA_100069942	27.72	37.39	EC:1.1.1.157	3-hydroxybutyryl-CoA dehydrogenase / 3-hydroxyacyl-CoA dehydrogenase	uncultured marine group II euryarchaeote
MG-II	Guaymas21	GBIDBA_100015633	187.88	327.60	EC:2.3.1.16	acetyl-CoA acetyltransferase	Thalassospira xiamenensis
MG-II	Guaymas21	GBIDBA_1000288815	66.22	41.87	EC:1.3.8.7	Acyl-CoA dehydrogenase	uncultured marine group II euryarchaeote
MG-II	Guaymas21	GBIDBA_100007268	26.10	51.35	EC:1.3.8.7	Acyl-CoA dehydrogenase	uncultured marine group II euryarchaeote
MG-II	Guaymas21	GBIDBA_1000118517	32.87	45.53	EC:1.1.1.35	bifunctional 3-hydroxyacyl-CoA dehydrogenase/enoyl-CoA hydratase/isomerase family protein	uncultured marine group II euryarchaeote
MG-II	Guaymas21	GBIDBA_1000133210	83.09	83.39	EC:2.3.1.16	putative thiolase, N-terminal domain protein	uncultured marine microorganism HF4000_APKG1C9
MG-II	Guaymas21	GBIDBA_1000094017	38.15	73.52	EC:1.1.1.157	3-hydroxybutyryl-CoA dehydrogenase / 3-hydroxyacyl-CoA dehydrogenase	uncultured marine group II euryarchaeote
MG-II	Guaymas21	GBIDBA_1001140814	121.76	112.59	EC:2.3.1.9	acetyl-CoA acetyltransferase	uncultured marine group II euryarchaeote EF100_57A08
MG-II	Guaymas21	GBIDBA_100033038	62.72	21.15	EC:2.3.1.9	acetyl-CoA C-acetyltransferase	uncultured marine group II euryarchaeote
MG-II	Guaymas21	GBIDBA_1000167030	0.00	10.58	EC:2.3.1.9	acetyl-CoA C-acetyltransferase	uncultured marine group II euryarchaeote
MG-II	Guaymas21	GBIDBA_1000026979	21.33	74.50	EC:6.4.1.3	carboxyl transferase	uncultured organism MedDCM-OCT-S04-C1
MG-II	Guaymas21	GBIDBA_100009409	81.23	29.35	EC:4.2.1.55	enoyl CoA hydratase/isomerase	uncultured archaeon MedDCM-OCT-S05-C205
MG-II	Guaymas21	GBIDBA_1000032528	122.38	51.59	EC:4.2.1.18	enoyl-CoA hydratase / methylglutaconyl-CoA hydratase	uncultured marine group II euryarchaeote
MG-II	Guaymas21	GBIDBA_1000288810	115.89	101.19	EC:1.3.8.6	glutaryl-CoA dehydrogenase	uncultured marine group II euryarchaeote
MG-II	Guaymas21	GBIDBA_1000105327	47.95	50.53	EC:1.3.8.4	isovaleryl-CoA dehydrogenase	uncultured marine group II euryarchaeote
MG-II	Guaymas21	GBIDBA_100009873	15.65	29.02	EC:6.4.1.3	putative carboxyl transferase	uncultured marine group II euryarchaeote

MG-II	Guaymas22	GBIDBA_100004272	226.83	102.41	EC:2.3.1.16	acetyl-CoA acetyltransferase	Thalassospira xiamenensis
MG-II	Guaymas22	GBIDBA_1000517612	196.52	34.50	EC:1.3.8.7	Acyl-CoA dehydrogenase	uncultured marine group II euryarchaeote
MG-II	Guaymas22	GBIDBA_100006811	158.87	0.00	EC:1.1.1.157	3-hydroxybutyryl-CoA dehydrogenase / 3-hydroxyacyl-CoA dehydrogenase	uncultured marine group II euryarchaeote
MG-II	Guaymas22	GBIDBA_100007701	122.12	45.03	EC:1.1.1.35	3-hydroxyacyl-CoA dehydrogenase	Azospirillum amazonense
MG-II	Guaymas22	GBIDBA_1000356911	45.84	14.49	EC:6.4.1.3	carboxyl transferase	uncultured organism MedDCM-OCT-S04-C1
MG-II	Guaymas22	GBIDBA_1000040837	41.62	35.08	EC:1.1.1.157	3-hydroxybutyryl-CoA dehydrogenase / 3-hydroxyacyl-CoA dehydrogenase	uncultured marine group II euryarchaeote
MG-II	Guaymas22	GBIDBA_100007702	20.18	25.51		acyl-CoA dehydrogenase	Thalassospira profundimaris
MG-II	Guaymas22	GBIDBA_1000116614	0.00	12.43	EC:2.3.1.9	3-ketoacyl-CoA thiolase	uncultured marine group II euryarchaeote KM3-72-G3
MG-II	Guaymas22	GBIDBA_100116824	0.00	4.88	EC:6.4.1.3	putative carboxyl transferase	uncultured marine group II euryarchaeote
MG-II	Guaymas22	GBIDBA_1000031027	0.00	14.77	EC:6.4.1.4	methylcrotonoyl-CoA carboxylase	Streptosporangium roseum DSM 43021
MG-II	Guaymas22	GBIDBA_1000031028	0.00	17.66	EC:4.2.1.18	enoyl-CoA hydratase / methylglutaconyl-CoA hydratase	uncultured marine group II euryarchaeote
MG-II	Guaymas22	GBIDBA_1000035147	0.00	13.37	EC:1.3.8.7	Acyl-CoA dehydrogenase	uncultured marine group II euryarchaeote
MG-II	Guaymas22	GBIDBA_1000035152	0.00	26.19	EC:1.3.8.6	glutaryl-CoA dehydrogenase	uncultured marine group II euryarchaeote
MG-II	Guaymas22	GBIDBA_1000040824	0.00	18.42	EC:4.2.1.55	enoyl-CoA hydratase / 3-hydroxybutyryl-CoA dehydratase	uncultured marine group II euryarchaeote
MG-II	Guaymas22	GBIDBA_1000040840	0.00	17.42	EC:4.2.1.17	Enoyl-CoA hydratase / isomerase	uncultured marine group II euryarchaeote
MG-II	Guaymas22	GBIDBA_100004271	0.00	80.39	EC:1.1.1.35	3-hydroxyacyl-CoA dehydrogenase	Thiothrix nivea
MG-II	Guaymas22	GBIDBA_100006815	0.00	12.64	EC:1.3.8.4	isovaleryl-CoA dehydrogenase	uncultured marine group II euryarchaeote
MG-II	Guaymas22	GBIDBA_100007426	0.00	40.64	EC:2.3.1.16	putative thiolase, N-terminal domain protein	uncultured marine microorganism HF4000_APKG1C9
MG-II	Guaymas22	GBIDBA_1000074454	0.00	11.18	EC:1.1.1.35	bifunctional 3-hydroxyacyl-CoA dehydrogenase/enoyl-CoA hydratase/isomerase family protein	uncultured marine group II euryarchaeote
MG-II	Guaymas23	GBIDBA_1000102414	66.22	13.96	EC:1.3.8.6	glutaryl-CoA dehydrogenase	uncultured marine group II euryarchaeote
MG-II	Guaymas23	GBIDBA_1000102419	53.53	17.36	EC:1.3.8.7	Acyl-CoA dehydrogenase	uncultured marine group II euryarchaeote
MG-II	Guaymas23	GBIDBA_100125631	0.00	0.00	EC:3.3.1.1	adenosylhomocysteinase	Pseudoxanthomonas suwonensis 11-1
MG-II	Guaymas23	GBIDBA_1000001612	25.42	7.14	EC:1.3.8.7	Acyl-CoA dehydrogenase	uncultured marine group II euryarchaeote
MG-II	Guaymas23	GBIDBA_1000001698	8.36	0.00	EC:2.3.1.9	acetyl-CoA C-acetyltransferase	uncultured marine group II euryarchaeote
MG-II	Guaymas23	GBIDBA_1000029421	25.69	14.44	EC:2.3.1.16	putative thiolase, N-terminal domain protein	uncultured marine microorganism HF4000_APKG1C9
MG-II	Guaymas23	GBIDBA_1000425914	113.19	11.93	EC:1.1.1.35	bifunctional 3-hydroxyacyl-CoA dehydrogenase/enoyl-CoA hydratase/isomerase family protein	uncultured marine group II euryarchaeote
MG-II	Guaymas23	GBIDBA_100044472	51.38	0.00	EC:2.3.1.16	acetyl-CoA acetyltransferase	Thalassospira profundimaris
MG-II	Guaymas23	GBIDBA_100044473	20.09	1.69	EC:1.1.1.35	3-hydroxyacyl-CoA dehydrogenase	Thiothrix nivea
MG-II	Guaymas23	GBIDBA_100044474	66.83	0.00		acyl-CoA dehydrogenase	Phaeospirillum molischianum
MG-II	Guaymas23	GBIDBA_1000000799	19.80	8.35	EC:2.3.1.180	3-oxoacyl-(acyl-carrier-protein) synthase III	Candidatus Desulforudis audaxviator MP104C
MG-II	Guaymas23	GBIDBA_10000007112	143.38	9.12	EC:6.2.1.3	putative fatty-acid--CoA ligase	Oceanicaulis sp. HTCC2633
MG-II	Guaymas23	GBIDBA_10000007128	46.94	0.00	EC:6.4.1.3	putative carboxyl transferase	uncultured marine group II euryarchaeote
MG-II	Guaymas23	GBIDBA_10000007186	60.36	4.63	EC:4.2.1.17	Enoyl-CoA hydratase / isomerase	uncultured marine group II euryarchaeote

MG-II	Guaymas23	GBIDBA_10000007199	26.11	0.00	EC:1.1.1.157	3-hydroxybutyryl-CoA dehydrogenase / 3-hydroxyacyl-CoA dehydrogenase	uncultured marine group II euryarchaeote
MG-II	Guaymas23	GBIDBA_10000007217	24.34	20.53	EC:5.3.3.18	enoyl CoA hydratase	uncultured archaeon MedDCM-OCT-S05-C205
MG-II	Guaymas23	GBIDBA_10000007220	47.27	9.96	EC:4.2.1.55	enoyl CoA hydratase/isomerase	uncultured archaeon MedDCM-OCT-S05-C205
MG-II	Guaymas23	GBIDBA_1000063448	15.98	3.37	EC:1.3.8.4	isovaleryl-CoA dehydrogenase	uncultured marine group II euryarchaeote
MG-II	Guaymas23	GBIDBA_1000063452	97.24	9.65	EC:1.1.1.157	3-hydroxybutyryl-CoA dehydrogenase / 3-hydroxyacyl-CoA dehydrogenase	uncultured marine group II euryarchaeote
MG-II	Guaymas23	GBIDBA_1000066738	61.40	2.59	EC:6.4.1.3	carboxyl transferase	uncultured organism MedDCM-OCT-S04-C1
MG-II	Guaymas23	GBIDBA_1000070428	46.67	2.62	EC:6.4.1.4	carboxyltransferase subunit of acetyl-CoA carboxylase	Bacteriovorax marinus SJ
MG-II	Guaymas23	GBIDBA_1000070429	61.61	14.17	EC:4.2.1.18	enoyl-CoA hydratase / methylglutaconyl-CoA hydratase	uncultured marine group II euryarchaeote
MG-II	Guaymas23	GBIDBA_1000007881	74.81	9.96	EC:2.3.1.9	3-ketoacyl-CoA thiolase	uncultured marine group II euryarchaeote AD1000-18-D2
MG-II	Guaymas24	GBIDBA_1000088851	275.33	0.00	EC:4.2.1.18	enoyl-CoA hydratase / methylglutaconyl-CoA hydratase	uncultured marine group II euryarchaeote
MG-II	Guaymas24	GBIDBA_1000096433	218.62	0.00	EC:4.2.1.55	enoyl CoA hydratase/isomerase	uncultured marine group II euryarchaeote
MG-II	Guaymas24	GBIDBA_1000091434	176.52	0.00	EC:1.3.8.4	isovaleryl-CoA dehydrogenase	uncultured marine group II euryarchaeote
MG-II	Guaymas24	GBIDBA_100014364	123.99	13.06	EC:2.3.1.9	acetyl-CoA C-acetyltransferase	uncultured marine group II euryarchaeote
MG-II	Guaymas24	GBIDBA_1000096422	93.15	0.00	EC:4.2.1.17	crotonase/enoyl-coenzyme A (CoA) hydratase superfamily protein	uncultured marine group II euryarchaeote
MG-II	Guaymas24	GBIDBA_1000097210	93.09	9.81	EC:6.4.1.3	putative carboxyl transferase	uncultured marine group II euryarchaeote
MG-II	Guaymas24	GBIDBA_100017234	89.65	18.89	EC:6.4.1.3	carboxyl transferase	uncultured marine group II euryarchaeote
MG-II	Guaymas24	GBIDBA_100003756	85.60	18.03	EC:1.1.1.157	3-hydroxybutyryl-CoA dehydrogenase / 3-hydroxyacyl-CoA dehydrogenase	uncultured marine group II euryarchaeote
MG-II	Guaymas24	GBIDBA_1000096417	84.70	0.00	EC:4.2.1.17	Enoyl-CoA hydratase / isomerase	uncultured marine group II euryarchaeote
MG-II	Guaymas24	GBIDBA_100011254	69.01	7.27	EC:1.1.1.35	bifunctional 3-hydroxyacyl-CoA dehydrogenase/enoyl-CoA hydratase/isomerase family protein	uncultured marine group II euryarchaeote
MG-II	Guaymas24	GBIDBA_1000088850	67.87	0.00	EC:6.4.1.4	carboxyltransferase subunit of acetyl-CoA carboxylase	Bacteriovorax marinus SJ
MG-II	Guaymas24	GBIDBA_100047599	60.15	0.00		Acyl-CoA dehydrogenase	uncultured marine group II euryarchaeote
MG-II	Guaymas24	GBIDBA_1000096416	59.42	0.00	EC:2.3.1.9	putative beta-ketoadipyl CoA thiolase	Anaerolinea thermophila UNI-1
MG-II	Guaymas24	GBIDBA_100009147	38.32	20.18	EC:6.2.1.3	long-chain acyl-CoA synthetase	uncultured marine group II euryarchaeote
MG-II	Guaymas24	GBIDBA_100001998	0.00	0.00		putative carboxyl transferase	uncultured marine group II euryarchaeote
MG-II	Guaymas24	GBIDBA_100027166	0.00	0.00	EC:1.3.8.7	Acyl-CoA dehydrogenase	uncultured marine group II euryarchaeote
MG-II	Guaymas24	GBIDBA_100047593	0.00	13.10	EC:1.3.8.6	glutaryl-CoA dehydrogenase	uncultured marine group II euryarchaeote
MG-II	Guaymas24	GBIDBA_1000088865	0.00	0.00		hypothetical protein MG2_1670	uncultured marine group II euryarchaeote
MG-II	Guaymas24	GBIDBA_1000096420	0.00	0.00	EC:1.1.1.157	3-hydroxybutyryl-CoA dehydrogenase / 3-hydroxyacyl-CoA dehydrogenase	uncultured marine group II euryarchaeote
MG-II	Guaymas25	GBIDBA_100011725	9.43	15.91	EC:1.1.1.35	bifunctional 3-hydroxyacyl-CoA dehydrogenase/enoyl-CoA hydratase/isomerase family protein	uncultured marine group II euryarchaeote

MG-II	Guaymas25	GBIDBA_1000121418	10.13	0.00	EC:6.2.1.3	long-chain acyl-CoA synthetase	uncultured marine group II euryarchaeote
MG-II	Guaymas25	GBIDBA_1000123314	0.00	0.00	EC:1.3.8.7	Acyl-CoA dehydrogenase	uncultured marine group II euryarchaeote
MG-II	Guaymas25	GBIDBA_100307573	12.45	0.00	EC:1.3.8.4	isovaleryl-CoA dehydrogenase	uncultured marine group II euryarchaeote
MG-II	Guaymas25	GBIDBA_1000033518	5.68	0.00	EC:4.2.1.17	enoyl-CoA hydratase / methylglutaconyl-CoA hydratase	uncultured marine group II euryarchaeote
MG-II	Guaymas25	GBIDBA_1000033519	12.05	0.00	EC:6.4.1.4	3-methylcrotonyl-CoA carboxylase, carboxyltransferase component	uncultured marine group II euryarchaeote
MG-II	Guaymas25	GBIDBA_100036427	3.88	0.00	EC:2.3.1.9	acetyl-CoA C-acetyltransferase	uncultured marine group II euryarchaeote
MG-II	Guaymas25	GBIDBA_1000041836	0.00	4.74	EC:4.2.1.17	Enoyl-CoA hydratase / isomerase	uncultured marine group II euryarchaeote
MG-II	Guaymas25	GBIDBA_1000041842	22.02	18.57	EC:1.1.1.157	3-hydroxybutyryl-CoA dehydrogenase / 3-hydroxyacyl-CoA dehydrogenase	uncultured marine group II euryarchaeote
MG-II	Guaymas25	GBIDBA_1000041845	0.00	10.22	EC:5.3.3.18	enoyl CoA hydratase	uncultured archaeon MedDCM-OCT-S05-C205
MG-II	Guaymas25	GBIDBA_1000041856	0.00	13.74	EC:4.2.1.55	enoyl CoA hydratase/isomerase	uncultured archaeon MedDCM-OCT-S05-C205
MG-II	Guaymas25	GBIDBA_1000047347	0.00	0.00	EC:2.3.1.16	putative thiolase, N-terminal domain protein	uncultured marine microorganism HF4000_APKG1C9
MG-II	Guaymas25	GBIDBA_1000059253	24.77	10.44	EC:1.3.8.6	glutaryl-CoA dehydrogenase	uncultured marine group II euryarchaeote
MG-II	Guaymas25	GBIDBA_1000059258	20.80	7.01	EC:1.3.8.7	Acyl-CoA dehydrogenase	uncultured marine group II euryarchaeote
MG-II	Guaymas25	GBIDBA_100605882	0.00	0.00	EC:1.1.1.157	3-hydroxybutyryl-CoA dehydrogenase / 3-hydroxyacyl-CoA dehydrogenase	uncultured marine group II euryarchaeote
MG-II	Guaymas25	GBIDBA_1000061142	39.86	6.72	EC:1.3.8.4	isovaleryl-CoA dehydrogenase	uncultured marine group II euryarchaeote
MG-II	Guaymas25	GBIDBA_1000061153	0.00	9.22	EC:1.1.1.157	3-hydroxybutyryl-CoA dehydrogenase / 3-hydroxyacyl-CoA dehydrogenase	uncultured marine group II euryarchaeote
MG-II	Guaymas25	GBIDBA_100623702	11.16	0.00	EC:1.1.1.157	3-hydroxybutyryl-CoA dehydrogenase / 3-hydroxyacyl-CoA dehydrogenase	uncultured marine group II euryarchaeote
MG-II	Guaymas25	GBIDBA_100625444	0.00	0.00	EC:1.1.1.157	3-hydroxybutyryl-CoA dehydrogenase / 3-hydroxyacyl-CoA dehydrogenase	uncultured marine group II euryarchaeote
MG-II	Guaymas25	GBIDBA_100656932	0.00	0.00	EC:1.3.8.4	isovaleryl-CoA dehydrogenase	uncultured marine group II euryarchaeote
MG-II	Guaymas25	GBIDBA_1000093249	7.93	6.69	EC:2.3.1.9	acetyl-CoA C-acetyltransferase	uncultured marine group II euryarchaeote
MG-II	Guaymas26	GBIDBA_1000058826	0.00	0.00	EC:1.1.1.157	3-hydroxybutyryl-CoA dehydrogenase / 3-hydroxyacyl-CoA dehydrogenase	uncultured marine group II euryarchaeote
MG-II	Guaymas26	GBIDBA_1000033024	0.00	9.16	EC:1.1.1.157	3-hydroxybutyryl-CoA dehydrogenase / 3-hydroxyacyl-CoA dehydrogenase	uncultured marine group II euryarchaeote
MG-II	Guaymas26	GBIDBA_1000072024	6.03	0.00	EC:6.4.1.4	3-methylcrotonyl-CoA carboxylase, carboxyltransferase component	uncultured marine group II euryarchaeote
MG-II	Guaymas26	GBIDBA_1000035837	0.00	0.00	EC:2.3.1.16	acetyl-CoA acetyltransferase	Acidovorax sp. CF316
MG-II	Guaymas26	GBIDBA_1000047631	0.00	0.00	EC:2.3.1.9	acetyl-CoA C-acetyltransferase	uncultured marine group II euryarchaeote
MG-II	Guaymas26	GBIDBA_1000031645	12.68	0.00	EC:1.3.8.7	Acyl-CoA dehydrogenase	uncultured marine group II euryarchaeote
MG-II	Guaymas26	GBIDBA_1000045913	8.30	0.00	EC:1.3.8.7	Acyl-CoA dehydrogenase	uncultured marine group II euryarchaeote
MG-II	Guaymas26	GBIDBA_1000118124	14.07	0.00	EC:1.1.1.35	bifunctional 3-hydroxyacyl-CoA dehydrogenase/enoyl-CoA hydratase/isomerase family protein	uncultured marine group II euryarchaeote
MG-II	Guaymas26	GBIDBA_100018284	0.00	0.00	EC:6.4.1.3	carboxyl transferase	uncultured organism MedDCM-OCT-S04-C1
MG-II	Guaymas26	GBIDBA_1000033026	0.00	0.00	EC:5.3.3.18	enoyl CoA hydratase	uncultured archaeon MedDCM-OCT-S05-

							C205
MG-II	Guaymas26	GBIDBA_1000033037	0.00	0.00	EC:4.2.1.55	enoyl CoA hydratase/isomerase	uncultured archaeon MedDCM-OCT-S05-C205
MG-II	Guaymas26	GBIDBA_1000033041	0.00	0.00	EC:4.2.1.17	Enoyl-CoA hydratase / isomerase	uncultured marine group II euryarchaeote
MG-II	Guaymas26	GBIDBA_1000072023	0.00	0.00	EC:4.2.1.17	enoyl-CoA hydratase / methylglutaconyl-CoA hydratase	uncultured marine group II euryarchaeote
MG-II	Guaymas26	GBIDBA_1000071723	24.83	10.47	EC:1.3.8.6	glutaryl-CoA dehydrogenase	uncultured marine group II euryarchaeote
MG-II	Guaymas26	GBIDBA_1000058820	12.20	0.00	EC:1.3.8.4	isovaleryl-CoA dehydrogenase	uncultured marine group II euryarchaeote
MG-II	Guaymas26	GBIDBA_1000078754	0.00	4.28	EC:6.2.1.3	long-chain acyl-CoA synthetase	uncultured marine group II euryarchaeote
MG-II	Guaymas26	GBIDBA_1000013948	34.73	2.44	EC:6.4.1.3	putative carboxyl transferase	uncultured marine group II euryarchaeote
MG-II	Guaymas26	GBIDBA_10000358103	0.00	7.80	EC:1.1.1.95	putative D-3-phosphoglycerate dehydrogenase	uncultured marine group II euryarchaeote
MG-II	Guaymas27	GBIDBA_100101815	4.21	0.00	EC:1.3.8.7	Acyl-CoA dehydrogenase	uncultured marine group II euryarchaeote
MG-II	Guaymas27	GBIDBA_100104341	24.94	0.00	EC:6.4.1.3	putative carboxyl transferase	uncultured marine group II euryarchaeote
MG-II	Guaymas27	GBIDBA_100123858	11.13	28.14	EC:4.2.1.17	Enoyl-CoA hydratase / isomerase	uncultured marine group II euryarchaeote
MG-II	Guaymas27	GBIDBA_100180231	0.00	0.00	EC:6.4.1.3	putative carboxyl transferase	uncultured marine group II euryarchaeote
MG-II	Guaymas27	GBIDBA_100197447	0.00	12.30	EC:4.2.1.17	enoyl CoA hydratase	uncultured archaeon MedDCM-OCT-S05-C205
MG-II	Guaymas27	GBIDBA_100241622	8.45	0.00	EC:1.3.8.7	Acyl-CoA dehydrogenase	uncultured marine group II euryarchaeote
MG-II	Guaymas27	GBIDBA_100262881	0.00	0.00	EC:4.2.1.55	enoyl-CoA hydratase / 3-hydroxybutyryl-CoA dehydratase	uncultured marine group II euryarchaeote
MG-II	Guaymas27	GBIDBA_100444163	25.44	0.00	EC:2.3.1.9	acetyl-CoA C-acetyltransferase	uncultured marine group II euryarchaeote
MG-II	Guaymas27	GBIDBA_100474145	29.58	18.70	EC:2.3.1.9	acetyl-CoA acetyltransferase	uncultured marine group II euryarchaeote HF70_59C08
MG-II	Guaymas27	GBIDBA_100048827	0.00	0.00		conserved F390 synthetase-related protein	uncultured marine group II euryarchaeote
MG-II	Guaymas27	GBIDBA_100048824	0.00	5.78		putative 3-oxoacyl-	acyl-carrier-protein synthase III
MG-II	Guaymas27	GBIDBA_100503852	31.79	5.36	EC:6.4.1.3	carboxyl transferase	uncultured organism MedDCM-OCT-S04-C1
MG-II	Guaymas27	GBIDBA_1000513911	16.60	7.00	EC:2.3.1.16	acetyl-CoA acetyltransferase	uncultured marine group II euryarchaeote
MG-II	Guaymas27	GBIDBA_1000608311	0.00	10.93	EC:1.3.8.6	glutaryl-CoA dehydrogenase	uncultured marine group II euryarchaeote
MG-II	Guaymas27	GBIDBA_100063557	4.13	3.48	EC:1.3.8.6	glutaryl-CoA dehydrogenase	uncultured marine group II euryarchaeote
MG-II	Guaymas27	GBIDBA_100846005	39.72	12.56	EC:6.2.1.3	long-chain acyl-CoA synthetase	uncultured marine group II euryarchaeote
MG-II	Guaymas27	GBIDBA_100085473	30.88	6.01	EC:1.1.1.35	bifunctional 3-hydroxyacyl-CoA dehydrogenase/enoyl-CoA hydratase/isomerase family protein	uncultured marine group II euryarchaeote
MG-II	Guaymas28	GBIDBA_100079412	11.44	0.00	EC:1.1.1.157	3-hydroxybutyryl-CoA dehydrogenase / 3-hydroxyacyl-CoA dehydrogenase	uncultured marine group II euryarchaeote
MG-II	Guaymas28	GBIDBA_100054532	0.00	0.00	EC:1.1.1.157	3-hydroxybutyryl-CoA dehydrogenase / 3-hydroxyacyl-CoA dehydrogenase	uncultured marine group II euryarchaeote
MG-II	Guaymas28	GBIDBA_100093506	0.00	5.08	EC:6.4.1.4	3-methylcrotonyl-CoA carboxylase, carboxyltransferase component	uncultured marine group II euryarchaeote
MG-II	Guaymas28	GBIDBA_1000466612	0.00	0.00	EC:6.2.1.1	acetate/CoA ligase	Haliangium ochraceum DSM 14365
MG-II	Guaymas28	GBIDBA_1000081716	3.98	0.00	EC:2.3.1.9	acetyl-CoA acetyltransferase	uncultured marine group II euryarchaeote HF70_39H11
MG-II	Guaymas28	GBIDBA_1000126246	0.00	13.72	EC:2.3.1.9	acetyl-CoA acetyltransferase	Deinococcus peraridilitoris DSM 19664
MG-II	Guaymas28	GBIDBA_1000126245	0.00	0.00	EC:2.3.1.9	acetyl-CoA acetyltransferase	Truepera radiovictrix DSM 17093
MG-II	Guaymas28	GBIDBA_100048029	12.58	49.49	EC:2.3.1.9	acetyl-CoA C-acetyltransferase	uncultured marine group II euryarchaeote

MG-II	Guaymas28	GBIDBA_1000143417	8.15	0.00	EC:2.3.1.9	acetyl-CoA C-acetyltransferase	uncultured marine group II euryarchaeote
MG-II	Guaymas28	GBIDBA_1000106350	12.42	0.00	EC:1.3.8.7	Acyl-CoA dehydrogenase	uncultured marine group II euryarchaeote
MG-II	Guaymas28	GBIDBA_1000126250	0.00	0.00	EC:1.3.8.7	Acyl-CoA dehydrogenase	uncultured marine group II euryarchaeote
MG-II	Guaymas28	GBIDBA_1000138536	0.00	3.95	EC:1.1.1.35	bifunctional 3-hydroxyacyl-CoA dehydrogenase/enoyl-CoA hydratase/isomerase family protein	uncultured marine group II euryarchaeote
MG-II	Guaymas28	GBIDBA_100116405	6.04	0.00	EC:6.4.1.3	carboxyl transferase	uncultured organism MedDCM-OCT-S04-C1
MG-II	Guaymas28	GBIDBA_100054534	0.00	0.00	EC:5.3.3.18	enoyl CoA hydratase	uncultured archaeon MedDCM-OCT-S05-C205
MG-II	Guaymas28	GBIDBA_1000033670	11.40	0.00	EC:4.2.1.55	enoyl CoA hydratase/isomerase	uncultured archaeon MedDCM-OCT-S05-C205
MG-II	Guaymas28	GBIDBA_100046156	11.09	0.00	EC:4.2.1.17	Enoyl-CoA hydratase / isomerase	uncultured marine group II euryarchaeote
MG-II	Guaymas28	GBIDBA_100093505	0.00	0.00	EC:4.2.1.17	enoyl-CoA hydratase / methylglutaconyl-CoA hydratase	uncultured marine group II euryarchaeote
MG-II	Guaymas28	GBIDBA_100034288	4.13	6.96	EC:1.3.8.6	glutaryl-CoA dehydrogenase	uncultured marine group II euryarchaeote
MG-II	Guaymas28	GBIDBA_1000103658	10.73	0.00	EC:6.2.1.3	long-chain acyl-CoA synthetase	uncultured marine group II euryarchaeote
MG-II	Guaymas28	GBIDBA_1000145424	0.00	0.00		putative carboxyl transferase	uncultured marine group II euryarchaeote
MG-II	Guaymas29	GBIDBA_1000111490	58.91	89.41	EC:2.3.1.9	acetyl-CoA acetyltransferase	uncultured marine group II euryarchaeote EF100_57A08
MG-II	Guaymas29	GBIDBA_1000130964	89.09	54.25	EC:2.3.1.180	3-oxoacyl-ACP synthase	Thermoanaerobacter tengcongensis MB4
MG-II	Guaymas29	GBIDBA_1000130968	42.60	20.53	EC:1.1.1.100	3-ketoacyl-(acyl-carrier-protein) reductase	uncultured marine group II euryarchaeote
MG-II	Guaymas29	GBIDBA_1000201491	34.23	4.12	EC:6.4.1.4	carboxyltransferase subunit of acetyl-CoA carboxylase	Bacteriovorax marinus SJ
MG-II	Guaymas29	GBIDBA_1000201492	76.75	12.94	EC:6.4.1.4	3-methylcrotonyl-CoA carboxylase, carboxyltransferase component	uncultured marine group II euryarchaeote
MG-II	Guaymas29	GBIDBA_1000201493	72.56	9.41	EC:4.2.1.18	enoyl-CoA hydratase / methylglutaconyl-CoA hydratase	uncultured marine group II euryarchaeote
MG-II	Guaymas29	GBIDBA_100228603	0.00	6.62	EC:2.3.1.9	acetyl-CoA acetyltransferase	uncultured marine group II euryarchaeote EF100_57A08
MG-II	Guaymas29	GBIDBA_100228863	0.00	0.00	EC:2.3.1.9	acetyl-CoA C-acetyltransferase	uncultured marine group II euryarchaeote
MG-II	Guaymas29	GBIDBA_1000231134	31.62	73.31	EC:1.3.99.1	succinate dehydrogenase flavoprotein subunit	uncultured marine group II euryarchaeote
MG-II	Guaymas29	GBIDBA_100261283	0.00	0.00	EC:5.3.3.18	enoyl CoA hydratase	uncultured archaeon MedDCM-OCT-S05-C205
MG-II	Guaymas29	GBIDBA_100261285	0.00	0.00	EC:1.1.1.157	hypothetical protein	uncultured archaeon MedDCM-OCT-S05-C205
MG-II	Guaymas29	GBIDBA_1000264820	40.68	29.02	EC:6.4.1.3	putative carboxyl transferase	uncultured marine group II euryarchaeote
MG-II	Guaymas29	GBIDBA_100270941	0.00	13.72	EC:1.3.8.6	glutaryl-CoA dehydrogenase	uncultured marine group II euryarchaeote
MG-II	Guaymas29	GBIDBA_1000299639	115.87	43.79	EC:1.3.8.4	isovaleryl-CoA dehydrogenase	uncultured marine group II euryarchaeote
MG-II	Guaymas29	GBIDBA_1000299643	74.10	28.83	EC:1.1.1.157	3-hydroxybutyryl-CoA dehydrogenase / 3-hydroxyacyl-CoA dehydrogenase	uncultured marine group II euryarchaeote
MG-II	Guaymas29	GBIDBA_100030542	343.13	118.35	EC:4.2.1.17	Enoyl-CoA hydratase / isomerase	uncultured marine group II euryarchaeote
MG-II	Guaymas29	GBIDBA_100030543	296.98	197.27	EC:4.2.1.17	Enoyl-CoA hydratase / isomerase	uncultured marine group II euryarchaeote
MG-II	Guaymas29	GBIDBA_100035436	229.51	303.33	EC:1.3.8.6	glutaryl-CoA dehydrogenase	uncultured marine group II euryarchaeote
MG-II	Guaymas29	GBIDBA_1000354312	49.67	27.92	EC:1.3.8.7	Acyl-CoA dehydrogenase	uncultured marine group II euryarchaeote
MG-II	Guaymas29	GBIDBA_100362852	0.00	0.00	EC:4.2.1.18	enoyl-CoA hydratase / methylglutaconyl-CoA	uncultured marine group II euryarchaeote

						hydratase	
MG-II	Guaymas29	GBIDBA_100362853	0.00	0.00	EC:6.4.1.4	3-methylcrotonyl-CoA carboxylase, carboxyltransferase component	uncultured marine group II euryarchaeote
MG-II	Guaymas29	GBIDBA_1000378210	90.82	54.05	EC:4.2.1.55	enoyl CoA hydratase/isomerase	uncultured archaeon MedDCM-OCT-S05-C205
MG-II	Guaymas29	GBIDBA_1000378213	38.55	21.67	EC:5.3.3.18	crotonase/enoyl-coenzyme A (CoA) hydratase superfamily protein	uncultured marine group II euryarchaeote
MG-II	Guaymas29	GBIDBA_1000378214	10.44	13.21	EC:1.1.1.157	3-hydroxybutyryl-CoA dehydrogenase / 3-hydroxyacyl-CoA dehydrogenase	uncultured marine group II euryarchaeote
MG-II	Guaymas29	GBIDBA_100406471	8.14	13.72	EC:2.3.1.16	acetyl-CoA acetyltransferase	Thalassospira xiamenensis
MG-II	Guaymas29	GBIDBA_100406472	0.00	9.36	EC:2.3.1.16	3-ketoacyl-CoA thiolase	Tistrella mobilis KA081020-065
MG-II	Guaymas29	GBIDBA_100406473	0.00	22.47	EC:1.1.1.35	3-hydroxyacyl-CoA dehydrogenase	Methylobacter tundripaludum
MG-II	Guaymas29	GBIDBA_100406474	0.00	0.00	EC:1.1.1.35	3-hydroxyacyl-CoA dehydrogenase	Methylomicrobium album
MG-II	Guaymas29	GBIDBA_100406476	0.00	0.00		acyl-CoA dehydrogenase	Reinekea blandensis
MG-II	Guaymas29	GBIDBA_1000413019	38.35	36.37	EC:1.1.1.35	bifunctional 3-hydroxyacyl-CoA dehydrogenase/enoyl-CoA hydratase/isomerase family protein	uncultured marine group II euryarchaeote
MG-II	Guaymas29	GBIDBA_1000066843	21.41	36.10	EC:2.3.1.16	putative thiolase, N-terminal domain protein	uncultured marine microorganism HF4000_APKG1C9
MG-II	Guaymas29	GBIDBA_10000668159	21.61	27.33		hypothetical protein MG2_0872	uncultured marine group II euryarchaeote
MG-II	Guaymas29	GBIDBA_10000668173	68.72	76.37	EC:6.4.1.3	carboxyl transferase	uncultured organism MedDCM-OCT-S04-C1
MG-II	Guaymas29	GBIDBA_1000830017	0.00	14.33	EC:6.2.1.3	putative long-chain-fatty-acid--CoA ligase	Plesiocystis pacifica
MG-II	Guaymas29	GBIDBA_1000087727	115.29	28.80	EC:2.3.1.16	acetyl-CoA acetyltransferase	Thalassospira profundimaris
MG-II	Guaymas29	GBIDBA_1000087728	69.18	41.17	EC:1.1.1.35	Enoyl-CoA hydratase	Caenispirillum salinarum
MG-II	Guaymas29	GBIDBA_1000087729	0.00	0.00		acyl-CoA dehydrogenase	Reinekea blandensis
MG-II	Guaymas29	GBIDBA_1000087730	30.75	42.13	EC:1.3.8.1	Acyl-CoA dehydrogenase	Rhodospirillum photometricum DSM 122
MG-II	Guaymas29	GBIDBA_1000091075	25.35	28.50	EC:1.3.8.7	Acyl-CoA dehydrogenase	uncultured marine group II euryarchaeote
MG-III	Cayman92	shallow_10061517	0.00	0.00	EC:6.2.1.16	acetoacetyl-CoA synthetase	Oceanospirillum maris DSM 6286
MG-III	Cayman92	shallow_10062551	337.23	328.23	EC:2.3.1.9	acetyl-CoA acetyltransferase	Methanomassiliicoccus luminyensis B10
MG-III	Cayman92	shallow_10117573	7.56	53.15	EC:6.2.1.1	acetyl-CoA synthetase	Proteobacteria bacterium JGI 0000113-P07
MG-III	Cayman92	shallow_100187519	18.61	0.00	EC:4.2.1.17	acyl-CoA hydratase	Frankia sp. QA3
MG-III	Cayman92	shallow_10062556	89.32	0.00	EC:6.2.1.3	AMPbinding enzyme domain containing protein	SAR406 cluster bacterium JGI 0000113-D11
MG-III	Cayman92	shallow_10100603	57.15	0.00	EC:4.2.1.55	enoyl-CoA hydratase I 1	Candidatus Chloracidobacterium thermophilum B
MG-III	Cayman92	shallow_10043688	18.94	0.00		methylmalonyl-CoA carboxyltransferase	Roseiflexus sp. RS-1
MG-III	Cayman93	Deep_10172425	0.00	0.00	EC:6.2.1.16	acetoacetyl-CoA synthetase	Oceanospirillum maris DSM 6286
MG-III	Cayman93	Deep_10073381	23.19	0.00	EC:2.3.1.9	acetyl-CoA acetyltransferase	Methanomassiliicoccus luminyensis B10
MG-III	Cayman93	Deep_10121944	136.20	0.00	EC:2.3.1.9	acetyl-CoA acetyltransferase	Methanomassiliicoccus luminyensis B10
MG-III	Cayman93	Deep_10121945	88.32	0.00	EC:2.3.1.9	acetyl-CoA acetyltransferase	Methanosarcina mazei Go1, DSM 3647
MG-III	Cayman93	Deep_10042586	68.69	44.93	EC:6.2.1.3	AMPbinding enzyme domain containing protein	SAR406 cluster bacterium JGI 0000113-D11
MG-III	Cayman93	Deep_10079013	0.00	0.00	EC:4.2.1.18	enoyl-CoA hydratase	Asticcacaulis biprosthecum C19, ATCC 27554
MG-III	Cayman93	Deep_10079016	23.37	0.00	EC:6.4.1.4	Methylcrotonoyl-CoA carboxylase beta chain	Novispirillum itersonii itersonii ATCC 12639
MG-III	Cayman93	Deep_10106521	25.91	0.00	EC:1.3.8.7	putative acyl-CoA dehydrogenase, C-terminal domain protein	uncultured marine group II euryarchaeote

MG-III	Guaymas32	GBIDBA_1000195319	2.71	0.00	EC:6.2.1.3	hypothetical protein	Paramecium tetraurelia strain d4-2
MG-III	Guaymas32	GBIDBA_1000127611	0.00	20.56	EC:6.2.1.3	hypothetical protein	Paramecium tetraurelia strain d4-2
MG-III	Guaymas32	GBIDBA_100072425	6.04	5.09	EC:6.4.1.4	Methylcrotonoyl-CoA carboxylase beta chain	Thermoplasmatales archaeon SCGC AB-539-N05
MG-III	Guaymas32	GBIDBA_1000039153	0.00	0.00	EC:6.4.1.4	Methylcrotonoyl-CoA carboxylase beta chain	Thermoplasmatales archaeon SCGC AB-539-N05
MG-III	Guaymas32	GBIDBA_1000039358	2.06	3.47	EC:1.1.1.35	3-hydroxyacyl-CoA dehydrogenase	Cycloclasticus sp. PY97M
MG-III	Guaymas32	GBIDBA_1000185122	0.00	1.76	EC:1.1.1.35	3-hydroxyacyl-CoA dehydrogenase	Thalassospira xiamenensis
MG-III	Guaymas32	GBIDBA_1000182227	0.00	0.00	EC:1.1.1.157	3-hydroxybutyryl-CoA dehydrogenase	Brevibacillus borstelensis
MG-III	Guaymas32	GBIDBA_1000039359	4.33	10.94	EC:2.3.1.16	acetyl-CoA acetyltransferase	Beijerinckia indica subsp. indica ATCC 9039
MG-III	Guaymas32	GBIDBA_1000185121	0.00	0.00	EC:2.3.1.16	acetyl-CoA acetyltransferase	Thiothrix nivea
MG-III	Guaymas32	GBIDBA_1000025815	8.19	6.91	EC:1.3.8.7	acyl-CoA dehydrogenase domain-containing protein	Salinarchaeum sp. Harcht-Bsk1
MG-III	Guaymas32	GBIDBA_1000077510	0.00	6.93	EC:1.3.8.7	acyl-CoA dehydrogenase domain-containing protein	Salinarchaeum sp. Harcht-Bsk1
MG-III	Guaymas32	GBIDBA_1000020210	0.00	0.00	EC:1.1.1.157	hypothetical protein	Paenisporosarcina sp. HGH0030
MG-III	Guaymas32	GBIDBA_1000195314	8.32	0.00	EC:2.3.1.9	acetyl-CoA acetyltransferase	Methanomassiliicoccus sp. Mx1-Issoire
MG-III	Guaymas32	GBIDBA_100012766	8.32	0.00	EC:2.3.1.9	acetyl-CoA acetyltransferase	Methanomassiliicoccus sp. Mx1-Issoire
MG-III	Guaymas32	GBIDBA_100116412	0.00	0.00	EC:2.3.1.9	acetyl-CoA acetyltransferase	Thermoplasmatales archaeon SCGC AB-539-N05
MG-III	Guaymas32	GBIDBA_100005393	0.00	3.49	EC:2.3.1.9	acetyl-CoA acetyltransferase	Thermoplasmatales archaeon SCGC AB-540-F20
MG-III	Guaymas32	GBIDBA_100123747	28.04	0.00	EC:6.4.1.3	carboxyl transferase	uncultured archaeon MedDCM-OCT-S05-C57
MG-III	Guaymas32	GBIDBA_100123748	0.00	6.93	EC:6.4.1.3	carboxyl transferase	Roseiflexus castenholzii DSM 13941
MG-III	Guaymas32	GBIDBA_1000119623	0.00	5.30	EC:6.4.1.3	carboxyl transferase	Chloroflexus aurantiacus J-10-fl
MG-III	Guaymas32	GBIDBA_1000039150	0.00	5.59	EC:4.2.1.18	enoyl-CoA hydratase	Alcanivorax hongdengensis
MG-III	Guaymas32	GBIDBA_100012767	0.00	7.82	EC:2.3.3.10	hypothetical protein H729_07190	Methanomassiliicoccus sp. Mx1-Issoire
MG-III	Guaymas32	GBIDBA_100056925	8.32	7.01	EC:1.3.8.6	putative acyl CoA dehydrogenase C terminal domain protein	uncultured archaeon MedDCM-OCT-S05-C724
MG-III	Guaymas32	GBIDBA_1000267215	0.00	0.00	EC:1.3.8.6	putative acyl CoA dehydrogenase C terminal domain protein	uncultured archaeon MedDCM-OCT-S05-C724
MG-III	Guaymas32	GBIDBA_1000181141	0.00	3.50	EC:1.3.99.2	putative acyl-CoA dehydrogenase, C-terminal domain protein	uncultured marine microorganism HF4000_APKG7N23
MG-III	Guaymas32	GBIDBA_10000077111	0.00	20.99	EC:1.3.99.2	putative acyl-CoA dehydrogenase, C-terminal domain protein	uncultured marine microorganism HF4000_APKG7N23
MG-III	Guaymas32	GBIDBA_1000181123	0.00	0.00	EC:4.2.1.55	short chain enoyl-CoA hydratase	Candidatus Chloracidobacterium thermophilum B
MG-III	Guaymas32	GBIDBA_1000007793	0.00	5.29	EC:4.2.1.55	short chain enoyl-CoA hydratase	Candidatus Chloracidobacterium thermophilum B
MG-III	Guaymas32	GBIDBA_1000185123	0.00	7.02		Acyl-CoA dehydrogenase	Micavibrio aeruginosavorus EPB
MG-III	Guaymas32	GBIDBA_1000039357	0.00	2.26		acyl-CoA dehydrogenase	Thalassospira xiamenensis
MG-III	Guaymas31	GBIDBA_1000358721	16.64	66.64	EC:2.3.1.9	acetyl-CoA acetyltransferase	Methanomassiliicoccus sp. Mx1-Issoire
MG-III	Guaymas31	GBIDBA_1000358716	5.45	13.78	EC:6.2.1.3	AMPbinding enzyme domain containing protein	Acanthamoeba castellanii str. Neff
MG-III	Guaymas31	GBIDBA_1000052546	12.36	5.21	EC:4.2.1.17	acyl-CoA hydratase	Frankia alni ACN14a

MG-III	Guaymas31	GBIDBA_100060474	16.64	31.57	EC:1.3.8.6	putative acyl-CoA dehydrogenase, C-terminal domain protein	uncultured marine microorganism HF4000_APKG10F13
MG-III	Guaymas31	GBIDBA_100006449	16.15	4.54		Acyl-CoA dehydrogenase	Rhodospirillum photometricum DSM 122
MG-III	Guaymas31	GBIDBA_1000129340	3.02	12.72	EC:6.4.1.4	Methylcrotonoyl-CoA carboxylase beta chain	Thermoplasmatales archaeon SCGC AB-539-N05
MG-III	Guaymas31	GBIDBA_1000233219	0.00	14.42	EC:1.1.1.157	3-hydroxyacyl-CoA dehydrogenase	Candidatus Chloracidobacterium thermophilum B
MG-III	Guaymas31	GBIDBA_1000245120	8.19	13.82	EC:1.3.8.7	acyl-CoA dehydrogenase	Anoxybacillus flavithermus
MG-III	Guaymas31	GBIDBA_100006448	4.19	10.60	EC:1.1.1.35	3-hydroxyacyl-CoA dehydrogenase	Thiothrix nivea
MG-III	Guaymas31	GBIDBA_100006447	4.27	14.40	EC:2.3.1.16	acetyl-CoA acetyltransferase	Sphingomonas wittichii RW1
MG-III	Guaymas31	GBIDBA_1000064491	0.00	13.44	EC:2.3.1.9	acetyl-CoA acetyltransferase	Thermoplasmatales archaeon SCGC AB-539-N05
MG-III	Guaymas31	GBIDBA_1000082861	29.05	24.49	EC:1.3.8.7	putative acyl-CoA dehydrogenase, C-terminal domain protein	uncultured marine microorganism HF4000_APKG7N23
MG-III	Guaymas31	GBIDBA_1000129337	6.45	21.75	EC:4.2.1.18	enoyl-CoA hydratase/isomerase	Asticcacaulis excentricus CB 48
MG-III	Guaymas31	GBIDBA_1000197228	0.00	0.00			
MG-III	Guaymas31	GBIDBA_1000033944	0.00	0.00		carboxyl transferase	Roseiflexus sp. RS-1
MG-III	Guaymas31	GBIDBA_1000358720	4.64	15.64	EC:2.3.3.10	hypothetical protein H729_07190	Methanomassiliicoccus sp. Mx1-Issoire
MG-III	Guaymas31	GBIDBA_100005259	12.65	10.66	EC:4.2.1.55	enoyl-CoA hydratase I 1	uncultured bacterium A1Q1_fos_2037

* indicate the relative abundance of transcripts (number of transcripts mapped was normalized to the length of the gene and total number of transcript reads from plume and background samples in each site).

Supplementary Table 9. The geranylgeranylglyceryl phosphate synthase (GGGPS) coding genes in different deep-sea archaeal groups

Taxonomy	Bin	Gene	Top Nr hit
MG-I	Cayman117	shallow_10008021	putative PcrB family protein
MG-I	Cayman118	shallow_101410110	putative PcrB family protein
MG-I	Lau19	TahiMoana_100376416	putative PcrB family protein
MG-I	Lau19	TuiMalila_10003134	putative PcrB family protein
MG-IIa	CM001443	EHR75768.1	geranylgeranylglyceryl phosphate synthase
MG-IIa	Guaymas25	GBIDBA_1000229914	putative geranylgeranylglyceryl phosphate synthase
MG-IIa	Guaymas26	GBIDBA_1000062036	putative geranylgeranylglyceryl phosphate synthase
MG-IIa	Guaymas28	GBIDBA_1000073519	geranylgeranylglyceryl phosphate synthase
MG-IIa	Lau34	KiloMoana_100019943	geranylgeranylglyceryl phosphate synthase
MG-IIa	Lau34	Mariner_100021221	geranylgeranylglyceryl phosphate synthase
MG-IIa	Lau34	TahiMoana_10024416	geranylgeranylglyceryl phosphate synthase
MG-IIb	Cayman51	Deep_100001912	geranylgeranylglyceryl phosphate synthase
MG-IIb	Cayman59	shallow_100023247	geranylgeranylglyceryl phosphate synthase
MG-IIb	Cayman59	shallow_100158416	geranylgeranylglyceryl phosphate synthase
MG-IIb	Cayman80	Deep_10012741	geranylgeranylglyceryl phosphate synthase
MG-IIb	Guaymas21	GBIDBA_1000861512	geranylgeranylglyceryl phosphate synthase
MG-IIb	Guaymas22	GBIDBA_1000111141	geranylgeranylglyceryl phosphate synthase
MG-IIb	Guaymas23	GBIDBA_1000284018	geranylgeranylglyceryl phosphate synthase
MG-IIb	Lau92	Abe_100004416	geranylgeranylglyceryl phosphate synthase
MG-IIb	Lau92	Abe_1000101710	geranylgeranylglyceryl phosphate synthase
MG-IIb	Lau92	KiloMoana_100032504	geranylgeranylglyceryl phosphate synthase
MG-IIb	Lau92	KiloMoana_100064584	geranylgeranylglyceryl phosphate synthase
MG-IIb	Lau92	Mariner_100029926	geranylgeranylglyceryl phosphate synthase
MG-IIb	Lau92	Mariner_10019471	geranylgeranylglyceryl phosphate synthase
MG-IIb	Lau92	Mariner_10150766	geranylgeranylglyceryl phosphate synthase
MG-IIb	SCGC_AAA288-C18	WP_019994066	geranylgeranylglyceryl phosphate synthase
MG-IIc	Cayman68	shallow_10048865	geranylgeranylglyceryl phosphate synthase

MG-IIc	Cayman69	Deep_10048895	geranylgeranylglyceryl phosphate synthase
MG-IIc	Guaymas24	GBIDBA_100028076	geranylgeranylglyceryl phosphate synthase
MG-IIc	Lau6	KiloMoana_1000018466	geranylgeranylglyceryl phosphate synthase
MG-IIc	Lau6	TahiMoana_100003142	geranylgeranylglyceryl phosphate synthase
MG-IIc	Lau93	KiloMoana_100012168	geranylgeranylglyceryl phosphate synthase
MG-IIc	Lau93	Mariner_10060493	geranylgeranylglyceryl phosphate synthase
MG-IIc	Lau93	TahiMoana_10024924	geranylgeranylglyceryl phosphate synthase
MG-III	Cayman92	shallow_100296610	geranylgeranylglyceryl phosphate synthase
MG-III	Cayman93	Deep_10073299	geranylgeranylglyceryl phosphate synthase
MG-III	Guaymas31	GBIDBA_1000033962	geranylgeranylglyceryl phosphate synthase
MG-III	Guaymas32	GBIDBA_1000119649	geranylgeranylglyceryl phosphate synthase
MG-III	Guaymas32	GBIDBA_1000260417	geranylgeranylglyceryl phosphate synthase

Supplementary Table 10. Genes involved in biosynthesis of lipids in uncultured MG-II archaea.

	**CM001443**	**Cayman51**	**Cayman59**	**Cayman68**	**Cayman69**	**Cayman80**	**Guaymas21**	**Guaymas22**	**Guaymas23**	**Guaymas24**	**Guaymas25**	**Guaymas26**	**Guaymas27**	**Guyamas28**	**Guaymas29**	**Guaymas34**	**Lau6**	**Lau34**	**Lau92**	**Lau93**
**Fatty acid synthesis**																				
acetyl-CoA carboxylase, biotin carboxylase subunit	EHR77240.1	Deep_100006757	shallow_100050941	shallow_10026684	Deep_10035453	Deep_10034379	GBIDBA_1000094039	GBIDBA_1000040856	GBIDBA_10000007173	GBIDBA_1000091440	GBIDBA_1000041816	GBIDBA_1000033014	GBIDBA_100085253	GBIDBA_1000461519	GBIDBA_1000305420	-	TahiMoana_100003736	TahiMoana_10027857	Mariner_100011813	-
3-oxoacyl-[acyl-carrier-protein] synthase III	EHR76531.1	Deep_100006318	shallow_10023752	-	-	Deep_10033527	-	-	-	-	-	-	GBIDBA_100048823	-	-	-	-	-	-	-
3-oxoacyl-[acyl-carrier-protein] synthase III	EHR76533.1	-	-	-	-	-	-	-	-	-	-	-	GBIDBA_100048824	-	-	-	-	-	-	-
3-ketoacyl-(acyl-carrier-protein) reductase	EHR75702.1	Deep_10000581	shallow_10053254	shallow_10111311	Deep_10061274	-	-	-	-	-	-	-	-	-	-	-	TahiMoana_100002629	KiloMoana_1000005186	-	-
3-ketoacyl-(acyl-carrier-protein) reductase	EHR76961.1	Deep_100006314	shallow_10023756	shallow_10074823	Deep_10045513	Deep_100335212	GBIDBA_100078996	GBIDBA_1000035192	GBIDBA_1000000795	GBIDBA_100046333	GBIDBA_1000202322	GBIDBA_1000013968	GBIDBA_100102173	GBIDBA_100014547	GBIDBA_1000130968	-	Mariner_10017714	KiloMoana_1000005186	Mariner_10002006	Mariner_100087011
enoyl-[acyl-carrier protein] reductase I	EHR77228.1	-	shallow_10016292	shallow_10049873	Deep_10045513	-	-	-	-	GBIDBA_1000091430	GBIDBA_100111602	GBIDBA_100003303	-	GBIDBA_1000110410	-	-	TahiMoana_100003726	Mariner_100002930	-	-
bifunctional 3-hydroxyacyl-CoA dehydrogenase/enoyl-CoA hydratase/isomerase family protein	EHR76046.1	Deep_100001960	shallow_10016697	shallow_10077074	Deep_10104666	Deep_10010158	GBIDBA_1000118517	GBIDBA_1000074454	GBIDBA_1000425914	GBIDBA_100011254	GBIDBA_100011725	GBIDBA_1000118124	GBIDBA_100085473	GBIDBA_1000138536	GBIDBA_1000413019	-	KiloMoana_100029385	Mariner_10003009	Mariner_100011833	KiloMoana_100030912
putative acyl-CoA hydrolase	EHR77235.	Deep_100006760	shallow_100050944	shallow_10026682	-	Deep_100317015	GBIDBA_1000094044	GBIDBA_1000040860	GBIDBA_10000007170	GBIDBA_1000091437	GBIDBA_1000041812	GBIDBA_1000033010	-	GBIDBA_1000110417	GBIDBA_1000305424	-	TahiMoana_100003733	TahiMoana_10027853	Mariner_10001188	KiloMoana_100098782

	1																			
**PUFA synthesis**																				
Polyketide synthase, beta-ketoacyl synthase domain	EHR77227.1	-	-	shallow_10049872	Deep_10076261	-	-	-	-	GBIDBA_100190931	GBIDBA_100111601	GBIDBA_1000174217	-	GBIDBA_100011049	-	-	Abe_100009071	Mariner_100002929	-	Mariner_10040334
putative acyl-CoA hydrolase	EHR77235.1	Deep_100006760	shallow_100050944	shallow_10026682	-	Deep_100317015	GBIDBA_1000094044	GBIDBA_1000040860	GBIDBA_10000007170	GBIDBA_1000091437	GBIDBA_1000041812	GBIDBA_1000033010	-	GBIDBA_1000110417	GBIDBA_1000305424	-	TahiMoana_100003733	TahiMoana_10027853	Mariner_10001188	KiloMoana_100098782
putative 4'-phosphopantetheinyl transferase	EHR77226.1	-	-	-	Deep_10076262	-	-	-	-	GBIDBA_1000091428	GBIDBA_100004183	GBIDBA_1000174215	-	GBIDBA_100011048	-	-	Abe_100057785	Mariner_100002928	-	Mariner_10040333
hioesterase superfamily protein	EHR76240.1	Deep_100010318	shallow_10054212	shallow_10073893	-	Deep_100076910	GBIDBA_100016133	GBIDBA_100007443	GBIDBA_10000016107	GBIDBA_1000143613	GBIDBA_1000136914	GBIDBA_1000045937	GBIDBA_100059877	GBIDBA_1000106330	-	-	TahiMoana_100007213	Mariner_10004558	-	-
steroyl-CoA desaturase (delta-9 fatty acid desaturase)	EHR76326.1	-	-	shallow_10110042	Deep_10060645	-	-	-	-	GBIDBA_1000037544	GBIDBA_1000123115	GBIDBA_1000035830	GBIDBA_100051391	GBIDBA_1000442214	-	-	TahiMoana_1000016103	TahiMoana_10040586	-	KiloMoana_100000266
putative lathosterol oxidase, fatty acid hydroxylase superfamily	EHR76623.1	-	-	-	-	-	-	-	-	GBIDBA_1000365812	GBIDBA_1000437211	-	GBIDBA_1000632710	GBIDBA_100030113	-	-	TahiMoana_100018519	-	-	-
**Ether-linked lipids synthesis**																				
putative geranylgeranyl reductase	EHR75748.1	Deep_100001928	shallow_10040242	shallow_10091752	-	Deep_100132714	GBIDBA_100134883	GBIDBA_1000042736	GBIDBA_100062392	GBIDBA_1000710413	GBIDBA_1000029760	GBIDBA_1000062020	GBIDBA_100126881	GBIDBA_100110945	-	-	Mariner_100014626	Mariner_10002122	Abe_1000044123	KiloMoana_100082747
putative geranylgeranyl reductase	EHR76783.1	Deep_1000059102	shallow_100067319	shallow_10047575	Deep_10067953	Deep_10020728	GBIDBA_100002695	GBIDBA_1000060955	GBIDBA_1000032644	GBIDBA_1000111323	GBIDBA_1000041452	GBIDBA_10000358119	GBIDBA_100045748	GBIDBA_100124552	GBIDBA_10000668103	-	TahiMoana_10004588	KiloMoana_1000005189	TahiMoana_10038052	-
4-hydroxybenzoate octaprenyltransferase	EHR76593.1	Deep_100005534	shallow_10009768	-	Deep_101131310	Deep_100110514	GBIDBA_1000073733	GBIDBA_1000068134	GBIDBA_1000063413	GBIDBA_100070294	GBIDBA_100088863	GBIDBA_100001668	-	-	GBIDBA_100029963	-	TahiMoana_100001659	-	Mariner_100046926	-
putative geranylgeranylglyceryl phosphate synthase	EHR75768.1	Deep_100001912	shallow_100023247	shallow_10048865	Deep_10048895	Deep_10012741	GBIDBA_1000861512	GBIDBA_1000111141	GBIDBA_1000284018	GBIDBA_100028076	GBIDBA_1000229914	GBIDBA_1000062036	GBIDBA_100212977	GBIDBA_1000073519	-	-	TahiMoana_100003142	TahiMoana_10024416	KiloMoana_100064584	Mariner_10060493
3-dehydroqu	EHR76	Deep_10000	shallow_100	shallow_100	Deep_10059	Deep_10062	GBIDBA_1000	GBIDBA_1000	GBIDBA_1000	GBIDBA_1000	GBIDBA_1000	GBIDBA_1000	GBIDBA_1000	GBIDBA_1000	GBIDBA_1000	GBIDBA_1002	Mariner_1000817	Mariner_1000199	Mariner_1000528	KiloMoana_100

inate synthase II	168.1	5868	02421	41147	705	906	072628	19454	001664	122516	123335	031626	930914	126229	091094	20596	5	6	31	066822
geranylgeranyl diphosphate synthase	EHR76653.1	Deep_100005937	shallow_10011642	shallow_10134267	Deep_10021796	Deep_10047679	GBIDBA_100013326	GBIDBA_1000074210	GBIDBA_1000029425	GBIDBA_1000037546	GBIDBA_1000047349	GBIDBA_1000035832	GBIDBA_100051395	GBIDBA_1000442211	GBIDBA_1000066839	-	TahiMoana_1000016105	TahiMoana_10040584	KiloMoana_1000015135	KiloMoana_100000268
octaprenyl diphosphate synthase / Geranylgeranylpyrophosphate synthase	EHR76258.1	Deep_100006378	shallow_100177912	shallow_10047574	Deep_10021796	Deep_10046504	GBIDBA_1000098718	GBIDBA_1000054155	GBIDBA_10000007143	GBIDBA_100015726	GBIDBA_1000089215	GBIDBA_100003163	GBIDBA_100151065	GBIDBA_1000106313	GBIDBA_100026484	-	TahiMoana_10001869	TahiMoana_10040584	KiloMoana_1000036521	KiloMoana_100000268
**Glycerophospholipids synthesis**																			
redicted Inorganic polyphosphate/ATP-NAD kinase	EHR77208.1	Deep_100000677	shallow_100187111	shallow_10070512	Deep_10062633	Deep_10010129	GBIDBA_100022489	GBIDBA_100004089	GBIDBA_10000007239	GBIDBA_1000091481	GBIDBA_1000041882	GBIDBA_1000072011	GBIDBA_100107652	GBIDBA_1000033645	GBIDBA_1000231112	-	Mariner_10028255	Mariner_10064753	Mariner_10049203	-
CDP-diglyceride synthetase	EHR76873.1	Deep_100001523	shallow_10033763	shallow_10074823	Deep_10059293	Deep_10019723	GBIDBA_100037733	GBIDBA_100021239	GBIDBA_1000007832	GBIDBA_1000057338	GBIDBA_1000011522	GBIDBA_1000006640	GBIDBA_100054795	GBIDBA_100012999	GBIDBA_1000155934	-	TahiMoana_10000582	TahiMoana_10004643	Mariner_10031671	Mariner_10014722
sphingosine kinase	EHR75625.1	Deep_100014738	shallow_10055661	shallow_10067834	Deep_10090812	Deep_10033892	-	GBIDBA_1000168019	GBIDBA_10000016198	GBIDBA_100038253	-	GBIDBA_100005139	-	-	-	-	-	TahiMoana_100012342	-	-
putative membrane-associated phospholipid phosphatase	EHR77128.1	Deep_100006724	shallow_10005098	shallow_100576811	Deep_10049251	Deep_10020861	GBIDBA_100009404	GBIDBA_1000040829	GBIDBA_10000007225	GBIDBA_1000088868	GBIDBA_1000041862	GBIDBA_100017426	-	GBIDBA_1000033664	GBIDBA_100037825	GBIDBA_100232416	TahiMoana_100002655	TahiMoana_10032102	Mariner_100011848	-
alpha/beta hydrolase fold protein	EHR76713.1	-	-	-	-	Deep_100033114	GBIDBA_100050828	-	GBIDBA_100044475	-	GBIDBA_1000047326	GBIDBA_1000035856	GBIDBA_100185285	GBIDBA_1000095531	GBIDBA_1000087731	GBIDBA_100412061	TahiMoana_100038831	Mariner_10026023	-	Mariner_100087013
putative Lysophospholipid acyltransferase (LPLAT) involved in glycerophospholipid biosynthesis	EHR75692.1	Deep_10036213	shallow_100068411	shallow_10070453	Deep_10106715	Deep_100193015	GBIDBA_1000156322	GBIDBA_1000042718	GBIDBA_1000088225	GBIDBA_1000210612	GBIDBA_1000029743	GBIDBA_100006204	GBIDBA_100241831	GBIDBA_1000177125	GBIDBA_100053055	GBIDBA_100292863	TahiMoana_100003175	Mariner_100015519	TahiMoana_10024423	-
putative Phospholipid/glycerol acyltransfe	EHR76156.1	-	shallow_100068411	shallow_10070453	Deep_10106715	Deep_100193015	-	-	-	GBIDBA_1000027246	GBIDBA_1000059216	-	-	GBIDBA_1000126241	-	-	Mariner_10013545	Mariner_10024565	TahiMoana_10024423	-

rase																				
1-acyl-sn-glycerol-3-phosphate acyltransferase or related phospholipid/glycerol acyltransferase	EHR76517.1	Deep_1000059126	shallow_10015767	-	Deep_10101217	Deep_10020306	GBIDBA_1000026936	-	GBIDBA_100002943	GBIDBA_100004474	GBIDBA_1000023464	GBIDBA_1000016622	GBIDBA_100059881	GBIDBA_1000282023	GBIDBA_10000668130	-	Mariner_10001006	Mariner_10023457	Mariner_10010415	-
NAD(P)H-dependent glycerol-3-phosphate dehydrogenase	EHR76134.1	Deep_100005878	shallow_10064066	shallow_10128042	-	Deep_100261712	GBIDBA_100016704	GBIDBA_100012045	-	GBIDBA_100021909	GBIDBA_1000138922	GBIDBA_100004768	GBIDBA_100181864	GBIDBA_1000126263	GBIDBA_100028572	-	TahiMoana_10004565	-	Mariner_10064935	-
putative integral membrane protein with PLC-likephosphodiesterase, TIM beta/alpha-barrel domain	EHR77263.1	Deep_1000019204	shallow_10059207	-	-	Deep_10076447	GBIDBA_1000032541	GBIDBA_1000031038	GBIDBA_10000007215	GBIDBA_1000111313	GBIDBA_1000136916	GBIDBA_1000078736	-	GBIDBA_1000434611	GBIDBA_100239496	-	TahiMoana_100045818	Mariner_10048265	Mariner_10012046	-
putative Glycerophosphoryl diester phosphodiesterase	EHR76937.1	Deep_100021525	shallow_100231522	shallow_10041755	Deep_10087976	Deep_10035274	GBIDBA_1000043723	GBIDBA_1000081342	GBIDBA_1000108341	GBIDBA_100018449	GBIDBA_1000064834	GBIDBA_1000025131	GBIDBA_100144655	GBIDBA_100106152	GBIDBA_1000091017	-	Mariner_100003626	Mariner_10021996	-	-
putative Membrane-associated phospholipid phosphatase	EHR76938.1	Deep_100006780	shallow_100050965	shallow_100576811	-	Deep_10020861	GBIDBA_100080458	GBIDBA_100047936	GBIDBA_10000007115	GBIDBA_100046338	GBIDBA_1000202329	GBIDBA_1000013963	GBIDBA_100144656	GBIDBA_100080995	GBIDBA_1000830011	-	Mariner_10017719	Mariner_10063641	Abe_100411791	-
Metal-dependent phosphoesterase (PHP family)	EHR76940.1	-	-	-	-	-	-	-	-	-	-	-	GBIDBA_100144658	GBIDBA_100080993	-	-	-	-	-	-

Supplementary Table 11. Transcripts of genes for flagellar proteins in deep-sea archaea genomes

Archaeal group	Genomic bins	Locus_Tag	Plume transcripts	Background transcripts	Annotation
MG-I	Cayman91	Deep_10074808	125.16	0.00	Flagellar biosynthesis/type III secretory pathway protein
MG-I	Guaymas69	GBIDBA_100827821	54.52	173.31	Flagellar FliJ protein
MG-I	Guaymas96	GBIDBA_101368972	0.00	19.66	Flagellar hook-associated protein 2 C-terminus
MG-I	Guaymas96	GBIDBA_100479962	0.00	30.32	Flagellar FliJ protein
MG-I	Guaymas96	GBIDBA_100029824	0.00	131.07	Flagellar capping protein
MG-II	Cayman59	shallow_10145586	0.00	35.93	Flagellar basal body protein FlaE
MG-II	Cayman80	Deep_10060343	17.67	0.00	Archaeal flagella assembly protein J
MG-II	Cayman80	Deep_10060342	99.64	0.00	Archaeal flagella assembly protein J
MG-II	Guaymas23	GBIDBA_1000159631	120.70	0.00	Flagella accessory protein C (FlaC)
MG-II	Guaymas23	GBIDBA_1000020865	220.29	15.47	Archaeal preflagellin peptidase FlaK
MG-II	Guaymas23	GBIDBA_1000020866	323.39	0.00	Archaeal flagella assembly protein J
MG-II	Guaymas23	GBIDBA_1000020867	354.21	0.00	Archaeal flagellar protein I FlaI
MG-II	Guaymas23	GBIDBA_1000020868	276.34	0.00	Archaeal flagellar protein FlaH
MG-II	Guaymas24	GBIDBA_1000079527	81.25	0.00	Archaeal flagella assembly protein J
MG-II	Guaymas24	GBIDBA_1000079528	84.80	0.00	Archaeal flagellar protein I FlaI
MG-II	Guaymas24	GBIDBA_1000079529	0.00	21.99	Archaeal flagellar protein FlaH
MG-II	Guaymas24	GBIDBA_1000079530	377.82	31.84	Archaeal flagella-related protein G, flaG
MG-II	Guaymas24	GBIDBA_1000079531	0.00	0.00	Archaeal flagellar protein FlaF
MG-II	Guaymas24	GBIDBA_1000079533	564.19	937.75	Archaeal flagellin FlaB
MG-II	Guaymas25	GBIDBA_1000110325	0.00	12.84	Archaeal flagella assembly protein J
MG-II	Guaymas25	GBIDBA_1000110326	0.00	0.00	Archaeal flagellar protein I FlaI
MG-II	Guaymas25	GBIDBA_1000110327	103.03	0.00	Archaeal flagellar protein FlaH
MG-II	Guaymas25	GBIDBA_1000110328	0.00	31.26	Archaeal flagella-related protein G, flaG
MG-II	Guaymas25	GBIDBA_100028822	427.77	12.88	Archaeal flagellin FlaB
MG-II	Guaymas25	GBIDBA_100028823	509.39	80.49	Archaeal flagellin FlaB
MG-II	Guaymas26	GBIDBA_100014322	40.63	0.00	Archaeal flagella assembly protein J
MG-II	Guaymas26	GBIDBA_100014323	21.27	4.48	Archaeal flagellar protein I FlaI
MG-II	Guaymas26	GBIDBA_100014324	0.00	0.00	Archaeal flagellar protein FlaH
MG-II	Guaymas26	GBIDBA_100014325	0.00	0.00	Archaeal flagella-related protein G, flaG
MG-II	Guaymas26	GBIDBA_100014328	1772.19	0.00	Archaeal flagellin FlaB
MG-II	Guaymas26	GBIDBA_100014329	949.10	25.00	Archaeal flagellin FlaB

MG-II	Guaymas27	GBIDBA_100106463	0.00	30.76	Archaeal preflagellin peptidase FlaK
MG-II	Guaymas27	GBIDBA_1000450321	121.27	0.00	Archaeal flagella assembly protein J
MG-II	Guaymas27	GBIDBA_1000450322	218.31	7.67	Archaeal flagellar protein I FlaI
MG-II	Guaymas27	GBIDBA_1000450323	118.64	25.00	Archaeal flagellar protein I FlaI
MG-II	Guaymas27	GBIDBA_1000613014	1859.83	81.64	Archaeal flagellin FlaB
MG-II	Guaymas27	GBIDBA_1000613015	2153.63	0.00	Archaeal flagellin FlaB
MG-II	Guaymas28	GBIDBA_1000192432	0.00	26.55	Archaeal flagellins
MG-II	Guaymas28	GBIDBA_1000192433	0.00	11.97	Archaeal flagellins
MG-II	Guaymas28	GBIDBA_1000388016	0.00	0.00	Archaeal flagella assembly protein J
MG-II	Guaymas28	GBIDBA_1000388017	0.00	0.00	Archaeal flagellar protein I FlaI
MG-II	Guaymas28	GBIDBA_1000388018	50.45	0	Archaeal flagellar protein FlaH
MG-II	Guaymas28	GBIDBA_1000388019	221.18	15.53	Archaeal flagella-related protein G, flaG
MG-II	Guaymas28	GBIDBA_100010361	0.00	0.00	Archaeal flagellin FlaB
MG-II	Guaymas28	GBIDBA_100010362	289.52	12.20	Archaeal flagellin FlaB
MG-II	Guaymas34	GBIDBA_100412063	0.00	10.04	Archaeal flagellar protein FliT
MG-III	Cayman92	shallow_10082225	224.89	69.26	Flp pilus assembly protein, ATPase CpaF
MG-III	Cayman92	shallow_10082226	114.48	0	Archaeal flagella assembly protein J
MG-III	Cayman92	shallow_10082227	304.80	0	Flp pilus assembly protein TadC
MG-III	Cayman92	shallow_10082228	49.39	5.786	Flp pilus assembly protein TadC
MG-III	Cayman92	shallow_10031431	859.13	82.89	Archaeal flagellin-like protein
MG-III	Cayman93	Deep_10052604	388.25	35.166	Flp pilus assembly protein, ATPase CpaF
MG-III	Cayman93	Deep_10052605	269.48	0.00	Archaeal flagella assembly protein J
MG-III	Cayman93	Deep_10058032	1854.64	0.00	Archaeal flagellin domain protein
MG-III	Guaymas31	GBIDBA_1000052528	195.39	172.91	Archaeal flagella assembly protein J
MG-III	Guaymas31	GBIDBA_1000052529	522.95	64.81	Flp pilus assembly protein TadC
MG-III	Guaymas31	GBIDBA_1000082848	1449.30	132.76	Archaeal flagellin-like protein
MG-III	Guaymas32	GBIDBA_100018955	1456.86	200.18	Archaeal flagellin-like protein
MG-III	Guaymas32	GBIDBA_100048225	1876.07	95.41	Archaeal flagellin-like protein

Supplementary Table 12. Central metabolism genes identified in each uncultured archaea genomic bins

Annotation	EC No.	MG-I						MG-II																				MG-III			
		Cayman91	Cayman117	Cayman118	Guaymas69	Guaymas96	Lau19	Cayman51	Cayman59	Cayman68	Cayman69	Cayman80	Guaymas21	Guaymas22	Guaymas23	Guaymas24	Guaymas25	Guaymas26	Guaymas27	Guaymas28	Guaymas29	Guaymas34	Lau6	Lau34	Lau92	Lau93	Cayman92	Cayman93	Guaymas31	Guaymas32
Pyruvate kinase	EC:2.7.1.40	0	0	0	0	0	0	0	0	0	0	1	0	0	0	0	2	1	0	1	0	0	0	0	0	0	0	0	0	0
Pyruvate:phosphate dikinase	EC:2.7.9.1	0	2	0	1	3	0	0	0	0	0	0	0	0	0	0	0	0	0	0	0	0	0	0	0	0	1	1	1	2
Fructose-1,6-bisphosphate aldolase	EC:4.1.2.13	0	0	1	0	0	0	2	2	2	1	1	2	2	2	2	1	2	0	0	2	2	7	2	1	0	1	0	1	2
Enolase	EC:4.2.1.11	0	1	1	1	0	0	1	2	0	0	1	1	1	1	2	1	0	0	1	2	0	3	1	1	0	1	2	1	2
Triosephosphate isomerase	EC:5.3.1.1	0	2	1	1	3	0	1	2	0	0	1	1	1	1	0	1	1	1	1	1	0	3	0	1	0	1	1	1	2
phosphomannose isomerase	EC:5.3.1.8	1	2	0	6	0	0	1	2	0	0	1	1	1	1	1	1	1	1	1	2	0	4	3	0	0	0	0	3	2
2,3-Bisphosphoglycerate-dependent phosphoglycerate mutase	EC:5.4.2.1	2	2	0	4	4	0	1	2	0	0	1	1	1	1	1	1	1	0	1	1	1	4	0	2	0	1	0	1	2
PPi-dependent phosphofructokinase	EC:2.7.1.90	0	0	0	0	0	0	0	0	0	0	0	0	0	0	0	0	0	0	0	0	0	0	0	0	0	0	0	0	2
Pyruvate:phosphate dikinase	EC:2.7.9.1	0	2	0	1	3	0	0	0	0	0	0	0	0	0	0	0	0	0	0	0	0	0	0	0	0	1	1	1	2
Phosphoenolpyruvate synthase	EC:2.7.9.2	0	0	1	0	0	0	0	1	1	1	0	0	0	0	1	1	1	2	0	1	0	5	3	0	0	0	0	0	0
Fructose-1,6-bisphosphatase	EC:3.1.3.11	2	2	0	0	2	0	1	2	1	0	0	1	1	1	1	1	1	0	1	1	0	4	0	1	0	1	0	1	0
Phosphoenolpyruvate carboxykinase	EC:4.1.1.49	0	1	0	3	2	0	1	2	2	0	2	1	1	1	1	1	1	1	1	1	0	4	1	3	0	0	0	1	2
Fructose-1,6-bisphosphate aldolase	EC:4.1.2.13	0	0	1	0	0	0	2	2	2	1	1	2	2	2	2	1	2	0	0	2	2	7	2	1	0	1	0	1	2
Enolase	EC:4.2.1.11	0	1	1	1	0	0	1	2	0	0	1	1	1	1	2	1	0	0	1	2	0	3	1	1	0	1	2	1	2
Triosephosphate isomerase	EC:5.3.1.1	0	2	1	1	3	0	1	2	0	0	1	1	1	1	0	1	1	1	1	1	0	3	0	1	0	1	1	1	2
phosphomannose isomerase	EC:5.3.1.8	1	2	0	6	0	0	1	2	0	0	1	1	1	1	1	1	1	1	1	2	0	4	3	0	0	0	0	3	2
Cupin-type phosphoglucose isomerase	EC:5.3.1.9	0	0	0	0	0	0	0	0	0	0	0	0	0	0	0	0	0	0	0	0	0	0	0	0	0	0	0	0	0
2,3-Bisphosphoglycerate-independent phosphoglycerate mutase	EC:5.4.2.1	2	2	0	4	4	0	1	2	0	0	1	1	1	1	1	1	1	0	1	1	1	4	0	2	0	1	0	1	2
Glucose 6-phosphate dehydrogenase	EC:1.1..1.49	0	0	0	0	0	0	0	0	0	0	0	0	0	0	0	0	0	0	0	0	0	0	0	0	0	0	0	0	0
Glucose dehydrogenase	EC:1.1.1.47	0	0	0	1	0	0	0	0	0	0	0	0	0	0	0	0	0	0	0	0	0	0	0	0	0	0	0	0	0
Glyceraldehyde dehydrogenase	EC:1.2.1.3	0	0	0	0	0	0	1	3	0	1	3	2	2	1	3	2	2	1	2	2	0	7	3	4	0	0	0	1	2
2-Keto-3-deoxy-D-gluconate kinase	EC:2.7.1.45	0	0	0	0	0	0	0	0	1	1	0	0	0	0	1	0	0	0	0	0	0	0	0	0	1	0	0	0	0
Transketolase	EC:2.2.1.1	0	4	1	8	2	2	2	5	4	2	2	2	1	2	4	2	2	0	2	2	0	8	4	6	2	0	0	0	2
Transaldolase	EC:2.2.1.2	0	2	1	0	1	0	0	0	0	0	0	0	0	0	1	1	0	1	0	0	0	2	0	0	1	1	1	0
Hexulose phosphate synthase	EC:4.1.2.43	0	0	0	0	0	0	0	0	0	0	0	0	0	0	0	0	0	0	0	0	0	0	0	0	0	0	0	0	0
Ribose 5-phosphate epimerase	EC:5.1.3.1	0	0	1	0	0	0	1	2	0	0	1	1	1	1	1	1	1	1	1	1	1	4	1	3	0	2	0	1	2
Phosphohexulose isomerase	EC:5.3.1.27	0	0	0	0	0	0	0	0	0	0	0	0	0	0	0	0	0	0	0	0	0	0	0	0	0	0	0	0	0
Ribose-5-phosphate isomerase	EC:5.3.1.6	0	1	1	3	1	0	1	2	0	2	1	1	0	1	1	1	1	1	1	1	0	4	3	0	0	0	1	1	2
Transketolase.	EC:2.2.1.1	0	4	1	8	2	2	2	5	4	2	2	2	1	2	4	2	2	0	2	2	0	8	4	6	2	0	0	0	2
Phosphoglycerate kinase.	EC:2.7.2.3	0	0	1	0	1	0	1	2	0	0	1	1	1	1	1	1	1	0	1	1	0	4	1	1	0	0	0	1	2
Fructose-bisphosphatase.	EC:3.1.3.11	2	2	0	0	2	0	1	2	1	0	0	1	1	1	1	1	1	0	1	1	0	4	0	1	0	1	0	1	0

Fructose-bisphosphate aldolase.	EC:4.1.2.13	0	0	1	0	0	0	2	2	2	1	1	2	2	2	2	1	2	0	0	2	2	7	2	1	0	1	0	1	2
Ribulose-phosphate 3-epimerase.	EC:5.1.3.1	0	0	1	0	0	0	1	2	0	0	1	1	1	1	1	1	1	1	1	1	1	4	1	3	0	2	0	1	2
Triose-phosphate isomerase.	EC:5.3.1.1	0	2	1	1	3	0	1	2	0	0	1	1	1	1	0	1	1	1	1	1	0	3	0	1	0	1	1	1	2
Ribose-5-phosphate isomerase.	EC:5.3.1.6	0	1	1	3	1	0	1	2	0	2	1	1	0	1	1	1	1	1	1	1	0	4	3	0	0	0	1	1	2
carbon monoxide dehydrogenase	EC:1.2.99.2	0	0	0	0	0	0	1	2	0	0	1	1	1	1	1	0	1	0	0	1	0	3	2	2	0	0	0	0	0
Acetyl-CoA synthetase	EC:6.2.1.1	1	2	1	2	1	1	0	0	0	0	0	1	0	0	1	1	1	0	2	0	0	4	0	0	1	1	0	1	2
Malate dehydrogenase.	EC:1.1.1.37	1	1	0	0	0	0	1	2	1	0	0	1	1	1	1	1	1	1	1	1	0	3	1	1	0	0	0	1	2
Isocitrate dehydrogenase (NADP(+)).	EC:1.1.1.42	0	1	0	0	1	1	1	1	1	1	1	1	1	1	1	2	0	1	1	2	0	3	4	4	1	1	1	1	2
2-oxoglutarate synthase.	EC:1.2.7.3	2	2	0	0	6	0	5	3	1	3	2	2	2	6	2	2	2	0	2	3	2	6	2	4	0	2	0	0	3
Succinate dehydrogenase.	EC:1.3.99.1	0	3	0	2	1	0	2	3	2	3	1	2	3	2	3	3	4	2	5	3	0	8	1	6	0	1	0	2	6
Pyruvate, water dikinase.	EC:2.7.9.2	0	0	1	0	0	0	0	1	1	1	0	0	0	0	1	1	1	2	0	1	0	5	3	0	0	0	0	0	0
Fumarate hydratase.	EC:4.2.1.2	0	0	0	0	0	0	1	0	1	0	1	1	1	1	1	1	1	1	1	1	1	3	1	0	0	1	0	1	2
Citrate--CoA ligase.	EC:6.2.1.18	0	0	0	0	0	0	0	0	0	0	0	0	0	0	0	0	0	0	0	0	0	0	0	0	0	0	0	0	0
Succinate--CoA ligase (ADP-forming).	EC:6.2.1.5	2	2	2	0	0	0	2	4	0	2	2	2	2	2	2	2	2	2	3	2	0	6	0	2	0	2	2	2	4
Methylmalonyl-CoA epimerase.	EC:5.1.99.1	1	3	0	0	2	1	1	2	1	0	0	1	1	1	1	1	1	1	1	1	0	4	2	8	2	0	0	1	2
Methylmalonyl-CoA mutase.	EC:5.4.99.2	3	5	0	0	3	3	3	5	3	2	3	2	3	3	3	4	3	1	4	6	0	6	5	6	5	2	1	2	6
Propionyl-CoA carboxylase.	EC:6.4.1.3	0	0	0	0	0	0	2	4	2	0	3	2	2	2	2	1	2	2	1	2	0	6	1	4	0	0	0	0	3
Oxidoreductases	EC:1.1.1.-	0	1	0	5	0	0	0	0	1	0	0	0	0	2	1	0	1	1	0	1	0	4	0	0	0	1	1	1	1
3-hydroxypropionate dehydrogenase (NADP(+))	EC:1.1.1.298	0	0	0	0	0	0	0	0	0	0	0	0	0	0	0	0	0	0	0	0	0	0	0	0	0	0	0	0	0
3-hydroxyacyl-CoA dehydrogenase.	EC:1.1.1.35	0	0	0	0	0	1	2	4	1	1	3	2	3	2	1	1	1	1	1	5	0	6	2	4	1	0	0	1	2
Acetyl-CoA C-acetyltransferase.	EC:2.3.1.9	1	5	1	2	1	1	2	4	1	3	3	3	1	2	2	2	2	1	5	1	0	6	1	2	2	1	3	2	4
Lyases. Carbon-oxygen lyases. Hydro-lyases.	EC:4.2.1.-	0	2	0	3	1	0	1	2	0	2	2	1	1	2	1	4	1	0	1	2	0	4	3	1	0	1	1	1	2
3-hydroxypropionyl-CoA dehydratase.	EC:4.2.1.116	0	0	0	0	0	0	0	0	0	0	0	0	0	0	0	0	0	0	0	0	0	0	0	0	0	0	0	0	0
4-hydroxybutanoyl-CoA dehydratase.	EC:4.2.1.120	0	1	1	1	3	0	0	0	0	0	0	0	0	0	0	0	0	0	0	0	0	0	0	0	0	0	0	0	0
Methylmalonyl-CoA epimerase.	EC:5.1.99.1	1	3	0	0	2	1	1	2	1	0	0	1	1	1	1	1	1	1	1	1	0	4	2	8	2	0	0	1	2
Vinylacetyl-CoA Delta-isomerase.	EC:5.3.3.3	0	0	0	0	0	0	0	0	0	0	0	0	0	0	0	0	0	0	0	0	0	0	0	0	0	0	0	0	0
Methylmalonyl-CoA mutase.	EC:5.4.99.2	3	5	0	0	3	3	3	5	3	2	3	2	3	3	3	4	3	1	4	6	0	6	5	6	5	2	1	2	6
Propionyl-CoA carboxylase.	EC:6.4.1.3	0	0	0	0	0	0	2	4	2	0	3	2	2	2	2	1	2	2	1	2	0	6	1	4	0	0	0	0	3
Malate dehydrogenase.	EC:1.1.1.37	1	1	0	0	0	0	1	2	1	0	0	1	1	1	1	1	1	1	1	1	0	3	1	1	0	0	0	1	2
Pyruvate synthase.	EC:1.2.7.1	0	0	0	0	0	0	0	0	0	0	0	0	0	0	0	0	0	0	0	0	0	0	0	0	0	0	0	0	0
Acetyl-CoA C-acetyltransferase.	EC:2.3.1.9	1	5	1	2	1	1	2	4	1	3	3	3	1	2	2	2	2	1	5	1	0	7	1	2	2	1	3	2	4
Pyruvate, water dikinase.	EC:2.7.9.2	0	0	1	0	0	0	0	1	1	1	0	0	0	0	1	1	1	2	0	1	0	5	3	0	0	0	0	0	0
Fumarate hydratase.	EC:4.2.1.2	0	0	0	0	0	0	1	0	1	0	1	1	1	1	1	1	1	1	1	1	1	3	1	0	0	1	0	1	2
Succinate--CoA ligase (ADP-forming).	EC:6.2.1.5	2	2	2	0	0	0	2	4	0	2	2	2	2	2	2	2	2	2	3	2	0	6	0	2	0	2	2	2	4
3-hydroxyacyl-CoA dehydrogenase.	EC:1.1.1.35	0	0	0	0	0	1	2	4	1	1	3	2	3	2	1	1	1	1	1	5	0	6	2	4	1	0	0	1	2
Acetyl-CoA C-acyltransferase.	EC:2.3.1.16	0	0	0	0	0	2	2	4	3	1	2	2	2	2	0	1	1	1	0	4	0	3	3	6	2	0	0	1	2
Enoyl-CoA hydratase.	EC:4.2.1.17	0	0	0	0	0	0	1	2	0	1	2	2	1	1	2	2	2	2	2	2	0	5	1	1	0	1	0	1	0
Long-chain-fatty-acid--CoA ligase.	EC:6.2.1.3	0	1	0	5	0	0	1	2	0	2	2	1	1	1	1	2	1	0	1	1	0	4	1	1	1	1	1	1	2
Pyruvate/2-oxoglutarate dehydrogenase complex, dehydrogenase (E1) component, alpha subunit	EC:1.2.4.1/ EC:1.2.4.4	0	0	0	1	0	0	1	2	1	1	1	2	2	1	2	2	2	2	2	1	1	4	1	2	0	0	0	1	2
Pyruvate/2-oxoglutarate dehydrogenase complex, dehydrogenase (E1) component, beta subunit	EC:1.2.4.1/ EC:1.2.4.4	0	0	0	1	0	0	1	2	1	0	1	2	2	1	1	2	2	2	2	1	1	4	2	2	0	0	0	1	2
Pyruvate/2-oxoglutarate dehydrogenase complex, dihydrolipoamide dehydrogenase (E2)	EC:1.8.1.4	0	0	0	0	0	0	1	2	1	0	1	1	1	0	1	1	1	0	2	1	0	4	1	1	0	0	0	0	2

component, and related enzymes																														
Pyruvate/2-oxoglutarate dehydrogenase complex, dihydrolipoamide dehydrogenase (E3) component, and related enzymes	EC:1.8.1.4	0	0	0	2	1	0	0	0	0	0	0	2	2	1	1	0	0	0	2	0	0	0	0	0	0	0	0	0	0
